# Loss of MFAP5 and Its Effect on Skin Homeostasis and Wound Healing

**DOI:** 10.1096/fj.202501770R

**Published:** 2025-12-05

**Authors:** Chen Han, Heidi Yuan, Timothy J. Koh, Lin Chen, Robert P. Mecham, Luisa A. DiPietro

**Affiliations:** ^1^ Center for Wound Healing and Tissue Regeneration University of Illinois Chicago Chicago Illinois USA; ^2^ Department of Kinesiology and Nutrition University of Illinois Chicago Chicago Illinois USA; ^3^ Department of Cell Biology and Physiology Washington University School of Medicine St. Louis Missouri USA

**Keywords:** fibroblasts, fibrosis, skin, wound healing

## Abstract

MFAP5, also known as microfibrillar‐associated glycoprotein‐2 (MAGP2), may influence parameters of skin wound healing related to scar formation. To further elucidate its role in skin wound healing, we assessed skin wound repair in *Mfap5* ^*−/−*^ mice. Loss of MFAP5 reduced external wound closure, angiogenesis, and enhanced neutrophil and macrophage wound influx. Loss of MFAP5 also reduced the deposition of total and mature collagen in uninjured normal skin (NS), but not in wounds. Furthermore, NS dermis of *Mfap5* ^−/−^ mice was thinner without any reduction in tensile strength. Single‐cell RNA‐sequencing of NS and wounds from *Mfap5* ^*+/+*^ and *Mfap5* ^*−/−*^ mice revealed two fibroblast subclusters that express MFAP5 more highly than other subclusters. Enrichment analysis of the differentially expressed genes (DEGs) in these two subclusters suggests these fibroblasts engage in extracellular matrix (ECM) deposition and angiogenesis. *Mfap5* ^*+/+*^ and *Mfap5* ^*−/−*^ fibroblasts also exhibit transcriptomic differences throughout in vivo wound healing, though as healing progressed, fewer differences were evident. To examine the direct effect of MFAP5 on fibroblasts outside of the wound space, fibroblasts were isolated from *Mfap5* ^*+/+*^ and *Mfap5* ^*−/−*^ mice for in vitro analysis. mRNA‐sequencing of *Mfap5* ^*+/+*^ and *Mfap5* ^*−/−*^ fibroblasts found genes involved in cellular migration and proliferation, ECM synthesis, and angiogenesis to be downregulated in *Mfap5* ^*−/−*^ fibroblasts vs. *Mfap5* ^*+/+*^ fibroblasts. Functionally, *Mfap5* ^*−/−*^ fibroblasts exhibited reduced migration, contractility, proliferation, and ECM deposition. Our findings suggest MFAP5 is a multifunctional glycoprotein in skin wound healing, as it has a role in regulating wound closure, angiogenesis, collagen deposition, inflammatory cell influx, and fibroblast behaviors related to scar formation.

## Graphical abstract

MFAP5's (or MAGP2's) role in skin wound healing was assessed in MFAP5‐deficient (*Mfap5* ^*−/−*^ *)* mice. In vivo, loss of MFAP5 reduced wound closure and angiogenesis, and enhanced wound inflammatory cell influx. *Mfap5* ^*−/−*^ mice also exhibited reduced total and mature collagen deposition in normal skin (NS). Single‐cell RNA‐sequencing analysis demonstrated that loss of MFAP5 significantly altered the fibroblast transcriptome in vivo. In vitro, *Mfap5* ^*−/−*^ fibroblasts exhibited reduced migration, contractility, proliferation, and ECM deposition.

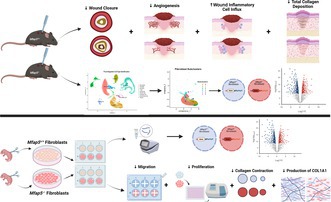

## 1 Introduction

Adult skin wounds generally heal with the formation of a scar. Although the regulation of scar formation is multifactorial, fibroblasts are a critical component of scar formation [1, 2]. Recent studies in our lab have demonstrated that wound fibroblasts develop a pro‐scarring phenotype following the engulfment of apoptotic cells [3]. These fibroblasts significantly express microfibrillar‐associated protein 5 (MFAP5) [4].

MFAP5, also referred to by its protein nomenclature microfibrillar‐associated glycoprotein‐2 (MAGP2), is an extracellular MFAP that associates with fibrillin to influence microfibril function [5]. MFAP5 has many avenues by which it may interact with and influence cell behavior. MFAP5 may regulate TGFβ signaling by binding to its active forms and Notch signaling by binding to Notch receptors or ligands [5, 6, 7, 8, 9]. MFAP5 regulation of Notch signaling is also cell‐type specific [8, 10, 11, 12]. Furthermore, MFAP5 is capable of binding αvβ3 integrin [5, 12, 13]. In the context of human pathology, MFAP5 is highly expressed in stromal fibroblasts of various human cancers, where it is associated with enhanced cancer fibrosis, angiogenesis, and chemoresistance, and in fibroblasts of fibrotic conditions such as idiopathic pulmonary fibrosis and systemic sclerosis [7, 8, 10, 14, 15, 16, 17, 18, 19, 20, 21, 22, 23, 24, 25]. Although MFAP5 has now been well studied in human pathologies, little attention has been paid to its role in skin wound healing [5, 26, 27, 28].

More recently, our laboratory has shown, through the use of loss‐of‐function and gain‐of‐function studies, that MFAP5 enhances wound angiogenesis and collagen deposition in vivo and fibroblast characteristics related to scar formation in vitro, including migration, collagen contractility, and expression of pro‐fibrotic genes [3, 4]. Here, we assess the role of MFAP5 in skin wound healing and fibroblast function using MFAP5‐deficient (*Mfap5* ^*−/−*^) mice. Our results not only validate the concept that MFAP5 is involved in wound angiogenesis and collagen deposition [4] but also show that MFAP5 may influence external wound closure and inflammatory cell influx in vivo. Moreover, we demonstrate that MFAP5 promotes fibroblast transcriptome and functions related to scar formation. Our work provides a deeper understanding of the role of MFAP5 in skin wound healing and suggests that MFAPs may be an important modulator of cell phenotype in skin wounds.

## 2 Materials and Methods

### 2.1 Animal Wound Models

All animal procedures and protocols were approved by the Institutional Animal Care and Use Committee at the University of Illinois, Chicago. This study also complied with the ARRIVE guidelines [29]. The *Mfap5* ^−/−^ mice used in this study were as previously described [26]. Eight‐ to 10‐week‐old male and female control C57BL/6 (*Mfap5* ^*+/+*^) (Jackson Laboratory, Bar Harbor, ME) and *Mfap5* ^*−/−*^ mice were housed in groups of 5 in a temperature‐controlled vivarium (22°C–24°C) on a 14‐h:10‐h light–dark cycle and provided with food and water *ad libitum*. *Mfap5* ^*−/−*^ mice are viable, breed well, and have a C57BL/6J mouse background [26]. Mice were anesthetized by intraperitoneal injection of ketamine (100 mg/kg) and xylazine (5 mg/kg) solution. The dorsal skin was shaved and cleaned with 70% isopropyl alcohol. Four full‐thickness excisional skin wounds were made using a 5‐mm punch‐biopsy instrument (Acu Punch, Acuderm Inc., Fort Lauderdale, FL) on the dorsal surface of each mouse relative to the dorsal midline. Wounds were symmetric on both sides of the midline and left uncovered.

The excised skin removed during wounding was used as the uninjured NS sample (day 0). On days 1, 3, 7, 14, and 21 post‐wounding, mice were euthanized by CO_2_ inhalation and cervical dislocation and wound tissue was quickly harvested. There were 5–9 mice per group and sex at each time point post‐wounding. A total of 152 mice were used for the excisional wound model assays. For immunofluorescent staining, tissues were snap frozen in optimum cutting temperature compound (Fisher Scientific, Hampton, NH), cut into 8 μm thick cryosections, and stored at −80°C. For Masson's trichrome staining, tissues were stored in 10% neutral buffered formalin solution, paraffin‐embedded, cut into 5 μm sections, and stored at room temperature. For proteomic analysis, tissues were snap‐frozen using dry ice. For single‐cell RNA‐sequencing analysis, tissues were processed as described later.

Assessment of wound breaking strength (WBS) was performed on an incisional skin wound model as described previously [30]. In brief, a 2‐cm longitudinal skin incision through the dermis and panniculus carnosus was made along the paraspinal area of each mouse and closed with surgical clips. Clips were removed on day 7 post‐wounding, and on days 14 and 21, the animals were sacrificed, and skin pelts were harvested for WBS analysis by isolating the healed wound. There were 4–7 mice per group and sex at each time point. A total of 57 mice were used for assessing WBS.

### 2.2 Wound Size Measurements

Wounds were photographed every other day during healing, and each wound's size was measured using AxioVision software (Zeiss, Oberkochen, Germany). Individual values from the 4 wounds from each mouse were averaged to produce a unique value per animal. The percentage of the open wound area compared to the original wound area and the percentage of the wound closed were calculated at each time point.

### 2.3 Hematoxylin and Eosin (H&E) Staining

Frozen tissue sections were stained by H&E using a standard protocol. *S*ections were imaged under 5× magnification using the Axioskop 40 fluorescence microscope (Zeiss).

### 2.4 Analysis of Wound Re‐Epithelialization

**FIGURE 1:**
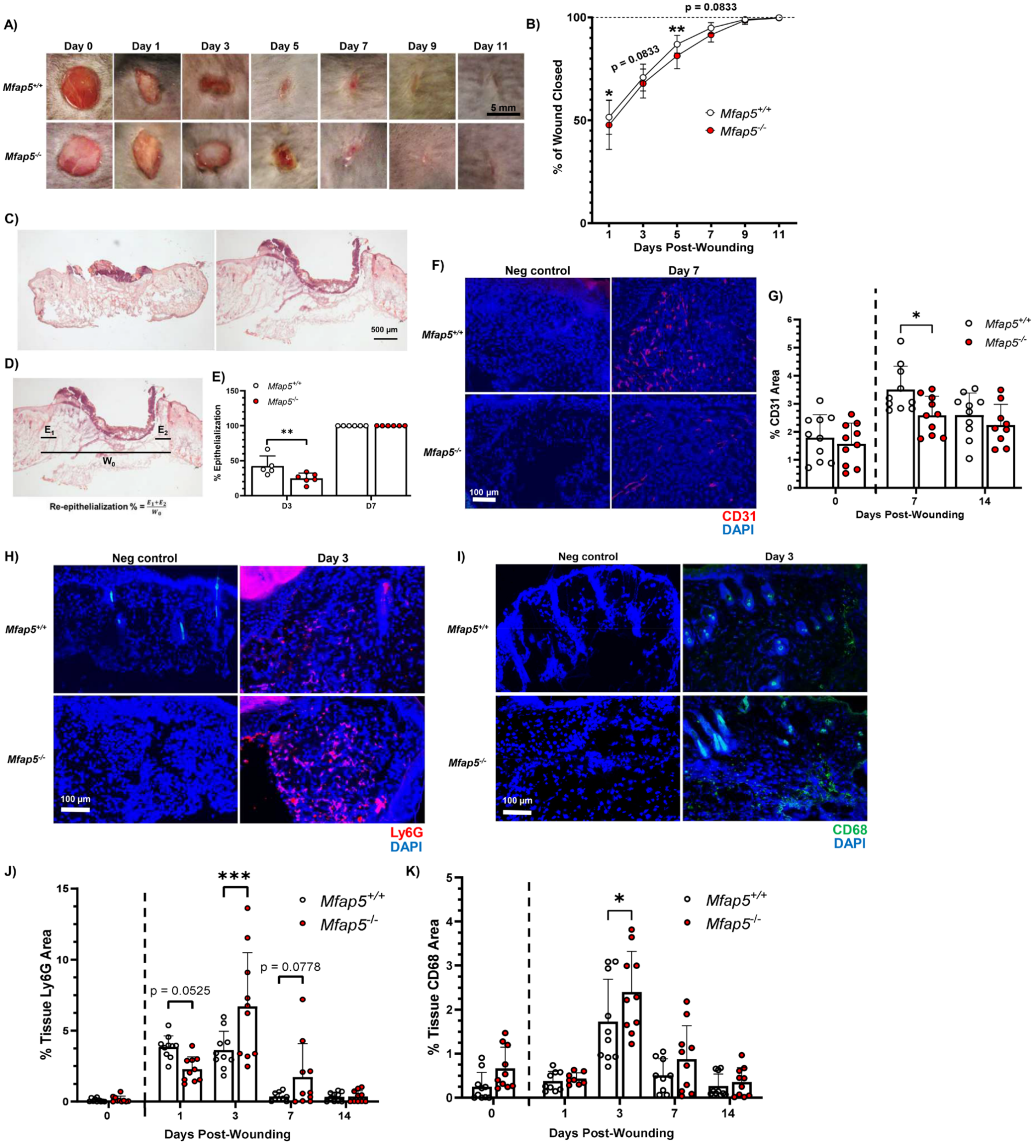
Loss of MFAP5 impairs early wound re‐epithelialization and angiogenesis, while altering wound inflammatory cell influx in vivo. (A) Representative photomicrographs of 5‐mm full‐thickness excisional dorsal skin wounds in *Mfap5* ^*+/+*^ and *Mfap5* ^*−/−*^ mice from days 0 to 11 post‐wounding. (B) Percent of wound closed at each time point post‐wounding in healing = *Mfap5* ^*+/+*^ and *Mfap5* ^*−/−*^ mouse wounds. *N* = 15–31. (C) Representative photomicrographs of hematoxylin and eosin (H&E) stained frozen wound tissues collected from *Mfap5* ^+/+^ or *Mfap5* ^−/−^ mice on Day 3 and 7, taken at 5× magnification. (D) Representation and formula for how wound re‐epithelialization was calculated. Labels: E_1_, E_2_ = distance from where the panniculus carnosus was cut during wounding to the tips of new epithelial cells, W_0_ = distance between the cut panniculus carnosus (the original wound distance). (E) Re‐epithelialization on Days 3 and 7 post‐wounding expressed as the percent of the original wound (*N* = 5–7). (F) Representative photomicrographs of fluorescence microscopy images of CD31 (red) staining in tissue from 7 days post‐wounding, taken at 20× magnification. (G) Vessel density in normal skin (NS) and in tissue from days 7 and 14‐post‐wounding expressed as the percent CD31 positive area in the NS dermis or wound bed. Representative photomicrographs of fluorescence microscopy images of Ly6G (red) (H) and CD68 (green) (I) staining in tissue from 3 days post‐wounding, taken at 20× magnification. Neutrophil or macrophage presence NS and in tissue from days 1‐, 3‐, 7‐, and 14‐post‐wounding expressed as the percent Ly6G positive area (J) or percent CD68 positive area (K) in NS dermis or the wound bed. Nuclei in blue (DAPI) for E, G, and H. *N* = 9–10 for G, J, and K. Bars on all graphs indicate mean ± SD. *p < 0.05, **p < 0.01, ***p < 0.001. Two‐way ANOVA with two‐stage linear step‐up procedure of Benjamini, Krieger, and Yekutieli (vs *Mfap5* ^*+/+*^).

Re‐epithelialization was measured by histomorphometric analysis of 8 μm tissue sections from the central portion of the wound stained with H&E. Using a standard ocular grid, the distance between the muscle edges and the distance that the epithelium had traveled across the wound were measured. In order to determine the percent reepithelialization, the distance from where the panniculus carnosus was cut during wounding was measured to the tips of new epithelial cells on either side of the wound and divided by the distance between the cut panniculus carnosus and the original wound distance (Figure 1D).

### 2.5 Indirect Immunofluorescent Staining

Anti‐CD31 (BD Biosciences, San Diego, CA), Ly6G (BD Biosciences), and CD68 (Invitrogen) antibodies were used to detect endothelial cells, neutrophils, and macrophages, respectively, in cryosections of NS and skin wounds. Cryosections were air‐dried, rehydrated in PBS, and fixed in cold acetone. Sections were washed with PBS and blocked with 10% goat serum (Sigma‐Aldrich, St. Louis, MO, USA) at room temperature before incubation with primary antibodies overnight at 4°C.

To detect COL1A1 and COL3A1 in NS tissue, paraffin‐embedded tissue sections (5 μm thick) were used. Sections were de‐paraffinized and rehydrated using a standard protocol. Antigen retrieval for COL1A1 tissue staining was performed using Epitope Retrieval Solution (IHC world, Ellicott City, MD), while antigen retrieval for COL3A1 tissue staining was performed using 10 mM Sodium Citrate buffer (pH 6.0). Sections were steamed for 40 min and then cooled at room temperature for 20 min using the appropriate antigen retrieval solution. Sections were then blocked with 10% normal goat serum (Sigma Aldrich) in PBS. For COL3A1 tissue staining, sections were incubated with 0.3% Sudan Black B (Fisher Scientific, Catalog # 26150) in 70% ethanol for 1 h, dipped in 70% ethanol, and washed in PBS before blocking. Following blocking, tissue sections were incubated overnight at 4°C with rabbit antibodies against COL1A1 (Cell Signaling Technology, Danvers, MA) or COL3A1 (Novus Biologicals, Centennial, CO).

Rat IgG (BD Biosciences) and rabbit IgG (Jackson ImmunoResearch Laboratories, West Grove, PA) were used as controls for staining in cryosections and paraffin‐embedded sections. Following overnight incubation, slides were incubated with the following secondary antibodies at a 1:1000 dilution for 1 h at room temperature: Alexa Fluor 594 goat anti‐rat IgG (Invitrogen), Alexa Fluor 594 goat anti‐rabbit (Invitrogen), or Alexa Fluor 488 goat anti‐mouse IgG (Invitrogen). After washing with PBS, samples were mounted with 50% glycerol in PBS containing DAPI (Sigma‐Aldrich) for nuclear counterstaining. All images were taken under 20× magnification using the Axioskop 40 fluorescence microscope (Zeiss). To assess vascularity, the percentage of the NS or wound bed that was CD31+ was quantified. To assess neutrophil and macrophage influx, the percentage of the NS or wound bed that was Ly6G+ and CD68+ was quantified, respectively. To assess COL1A1 and COL3A1 content, the integrated density of COL1A1+ and COL3A1+ staining in NS dermis relative to the dermal area was quantified. All quantifications were performed with ImageJ [31]. The concentration, dilution, and incubation conditions for the antibodies used are shown in Table 1.

**TABLE 1:** Antibody list with their dilution factor and incubation conditions.

Antibody	Company	Catalog number	Host	Final concentration	Incubation conditions	Application
Anti‐CD31	BD Bioscience	557 355	Rat	0.3125 μg/mL	4°C Overnight (O/N)	Immunofluorescence (IF)
Anti‐Ly6G	BD Bioscience	551 459	Rat	1.0 μg/mL	4°C O/N	IF
Anti‐CD68	Invitrogen	PA5‐78996	Rabbit	1.0 μg/mL	4°C O/N	IF
Anti‐COL1A1	Cell Signaling Technology	72026S	Rabbit	0.0933 μg/mL	4°C O/N	IF
Anti‐COL3A1	Novus Biologicals	NB600‐594	Rabbit	11.6 μg/mL	4°C O/N	IF
Anti‐COL1A1	Cell Signaling Technology	72026S	Rabbit	0.014 μg/mL	4°C O/N	Western blot (WB)
Anti‐COL3A1	ProteinTech	22 734–1‐AP	Rabbit	18 μg/mL	4°C O/N	WB
Anti‐ß‐Actin	Cell Signaling Technology	4967	Rabbit	0.0933 μg/mL	4°C O/N	WB
Anti‐COL3A1	ProteinTech	22 734–1‐AP	Rabbit	3.6 μg/mL	4°C O/N	Immunocytochemistry (ICC)
Anti‐COL1A1	SouthernBiotech	1310–01	Goat	1.6 μg/mL	4°C O/N	ICC
Alexa fluor 594 goat anti‐rat	Invitrogen	A‐11007	Goat	1.0 μg/mL	Room temperature (RT) °C for 1 h	IF (Secondary antibody)
Alexa fluor 594 goat anti‐rabbit	Invitrogen	A‐11012	Goat	1.0 μg/mL	RT for 1 h	IF (Secondary antibody)
Alexa fluor 488 goat anti‐mouse	Invitrogen	A‐11001	Goat	1.0 μg/mL	RT for 1 h	IF (Secondary antibody)
Alexa fluor 594 donkey anti‐goat	Invitrogen	A‐11058	Donkey	1.0 μg/mL	RT for 1 h	ICC (Secondary antibody)
Alexa fluor 594 donkey anti‐rabbit	Invitrogen	A‐21206	Donkey	1.0 μg/mL	RT for 1 h	ICC (Secondary antibody)

### 2.6 Masson's Trichrome Staining

Masson's trichrome stain (StatLab Medical Products, Mckinney, TX) was done as described by the manufacturer's protocol and our previous publications [4, 32, 33, 34, 35]. Images were taken at 20× and 10× magnification, and quantification was performed using ImageJ as described in our previous publications [31, 32, 33].

### 2.7 Picrosirius Red Staining

Picrosirius Red staining (Electron Microscopy Sciences, Hartfield, PA) was done as described by the manufacturer's protocol and our previous publications [33, 34, 35]. Images were taken with and without a polarized lens at 20× magnification, and quantification was performed using ImageJ as described in our previous publications [31, 34, 35]. Assessment of the ECM pattern and organization was performed using TWOMBLI [31, 36].

### 2.8 Measurement of Skin Dermis Thickness

Using Masson's Trichrome stained tissue and ImageJ, we measured the distance between the epithelial layer and the dermal white adipocyte tissue in NS and wounds to assess skin dermis thickness [31]. Six measurements spanning the tissue were averaged to produce 1 unique value per animal.

### 2.9 Assessment of Normal Skin and Wound Tissue Breaking Strength

An incisional skin wound model was used as described above. Assessment of WBS was performed as described previously [30, 37]. In summary, harvested skin pelts, consisting of healed wounds cut at right angles to the long axis of the wound, were placed in the jaws of a motorized tensiometer (Mark‐10, Copiague, NY). Skin pelts were then stretched at a constant rate until disruption occurred. Individual values were averaged to generate 1 unique value per animal. WBS was assessed in tissue from NS and wounds on days 14 and 21. WBS is expressed as pounds of force.

### 2.10 Total Collagen Assay

Weighted NS or collagen standard was hydrolyzed in 6 N HCl or 12 N HCl, incubated for 20 h at 95°C, cooled to room temperature, and centrifuged to collect supernatant. Then, a total collagen assay (QuickZyme Biosciences, Leidin, Netherlands) was performed as described by the manufacturer's protocol and our previous publications [38]. Results are expressed as μg collagen/mg tissue.

### 2.11 Single‐Cell RNA‐Sequencing of Normal Skin and Wound Tissue

#### 2.11.1 Cell Preparation

Wound tissues were collected from male and female *Mfap5* ^*+/+*^ or *Mfap5* ^*−/−*^ mice (*n* = 4–5 animals for each sex and each experimental group) on days 0 (D0), 3 (D3), and 7 (D7) post‐wounding. A total of 48–60 mice were used for single‐cell RNA‐sequencing. Collected wound tissues were pooled separately based on sex, experimental group, and time post‐wounding (Figure 3A). Tissue collected from D0 was considered NS. After collection and pooling, tissues were micro‐dissected, minced, and incubated at 37°C in a dispase (Worthington Biochemical Corporation, Lakewood, NJ, Catalog # 9001‐92‐7)/liberase (Roche, Basel, Switzerland, Catalog # 5401135001) solution supplemented with DNAse I (Sigma‐Aldrich, Catalog # D5025) and penicillin–streptomycin (Gibco, Waltham, MA, Catalog # 15–140‐122) for 60 min with constant rotation. The final concentrations for each solution component were 3.6 mg/mL dispase, 30 μg/mL liberase, 100 μg/mL DNAse I, and 100 Units/mL–100 μg/mL penicillin–streptomycin. Post‐incubation, cell suspensions were passed through a 40 μM cell strainer (ThermoFisher Scientific, Waltham, MA). To remove red blood cells and debris, single‐cell suspensions were treated with 1× RBC lysis buffer (Biolegend, San Diego, California, Catalog # 420301), washed, and resuspended in cold PBS before debris removal using the Debris Removal Solution (Miltenyi Biotec, North Rhine‐Westphalia, Germany, Catalog # 130‐109‐398) according to the manufacturer's protocol. All cells were then frozen in a mixture of 70% DMEM (Corning Inc., Corning, NY), 30% Fetal Bovine Serum (FBS) (GeminiBio, Sacramento, CA), and 10% DMSO (Sigma‐Aldrich) before being sent to Novogene (Novogene America, Sacramento, CA). Thawed cell suspensions underwent dead cell removal, and only samples with > 100 000 cells, > 70% viability, and minimal to no cell clumps were used for single‐cell RNA sequencing. GEM generation, barcoding, post‐GEM‐RT cleanup, cDNA amplification, and cDNA library construction were performed using Single‐cell 3′ v3 chemistry (10× Genomics, Pleasanton, California). Libraries were then sequenced on an Illumina NovaSeq 6000 (Illumina, San Diego, CA). Dead cell removal, cell counting and viability testing, GEM generation, barcoding, post‐GEM‐RT cleanup, cDNA amplification, library preparation, quality control, and sequencing were all performed by Novogene (Novogene America). Transcripts were mapped to the mouse reference genome (GRCm38/mm10) using Cell Ranger Version 7.0.0. The summary of Cell Ranger statistics on cells, sequencing, and mapping metrics pre‐quality control assessment for downstream bioinformatics analyses for cell, sequencing, and mapping metrics is summarized in Tables S1–S3.

#### 2.11.2 Data Quality Control Metrics for 3′‐End Transcripts

Before downstream analyses, low‐quality cells were removed and data integration was performed using the RPCA method [39]. Quality control metrics included keeping genes present in at least 10 cells and cells with at least 200 genes, while removing cells with either a mitochondrial gene expression greater than 10% or a multiplet likelihood score greater than 0.25. Post‐quality control, 72 876 cells remained for downstream bioinformatic analyses. All data quality control and downstream analyses were done by us using R version 4.4.1 with Bioconductor v3.20.

#### 2.11.3 Cell Type Identification and Fibroblast Subcluster Analysis

Clustering of cells was performed using the Seurat R package [40]. In brief, digital gene expression matrices were column‐normalized and log‐transformed. Principal component analysis (PCA) was first performed on the list of highly variable genes to identify cell clusters. Genes were selected for inclusion in PCA with an average expression > 0.01 and dispersion > 1.0. We used the Jackstraw method in Seurat to identify significant principal components (PCs). The first 10 PCs were used for clustering with the resolution set at 0.2. Cell cluster marker genes were identified using the FindAllMarkers() function according to the following criteria: adjusted *p*‐value (p.adj) < 0.05 and log(fold‐change) (Log2FC) > 0.25. To present high‐dimensional data in a two‐dimensional space, we performed UMAP analysis using the results of PCA with significant PCs as input. Differentially expressed marker genes used for cell type identification were selected using the following criteria: p.adj < 0.05, Log2FC > 2, pct.1 > 0.40 (percentage of cells where the gene is detected within the cluster), and pct.2 < 0.20 (percentage of cells where the gene is detected in other clusters). Differentially expressed marker genes meeting these cut‐offs were input into EnrichR, and likely cell types were identified using the top 5 enrichments from the following cell marker curated databases: (1) CellMarker 2024, Tabula Muris, and PanglaoDB [41, 42, 43, 44]. Known marker genes used for skin cell types in previously published single‐cell RNA‐sequencing studies were also used [2, 45, 46, 47].

We followed the same procedures as above for the sub‐clustering analysis of fibroblasts. The top 15 PCs were used for clustering, and 6 subclusters (subclusters 0–5) were obtained with a resolution = 0.1. Fibroblast subcluster marker genes were identified using FindAllMarkers() function according to the following criteria: p.adj < 0.05 and Log2FC > 0.25 [40]. To present high‐dimensional data in two‐dimensional space, we performed UMAP analysis using the results of PCA with significant PCs as input. DEG signatures for each fibroblast subcluster were analyzed to determine if a unique fibroblast subcluster differentially expressed MFAP5 as a marker gene in NS and during wound healing in vivo. Gene ontology (GO) enrichment for biological processes (BP) terms and Reactome pathway enrichment analysis was performed on DEGs with a p.adj < 0.05 and Log2FC > 1 for the fibroblasts subclusters that differentially expressed MFAP5 relative to other fibroblast subclusters via the EnrichR package in R [44, 48, 49]. GO BP terms with a p.adj < 0.05 following Benjamini and Hochberg's approach for controlling the false discovery rate (FDR) were considered statistically significantly enriched terms, and only the top 25 significantly enriched terms based on p.adj were included in our analyses.

#### 2.11.4 Analysis of DEGs in Mfap5^+/+^ and Mfap5^−/−^ Mice

We used the FindAllMarkers() function to identify DEGs between *Mfap5* ^*+/+*^ or *Mfap5* ^*−/−*^ fibroblasts at each time point based on the following criteria: p.adj < 0.01, average Log2FC > 0.25, and the percentage of cells where the gene is detected in either *Mfap5* ^*+/+*^ or *Mfap5* ^*−/−*^ fibroblasts (pct.1) is more than 25% (pct.1 > 0.25) [40]. GO BP and Reactome pathway term enrichment analysis was performed on the list of differentially upregulated and downregulated genes in *Mfap5* ^*−/−*^ fibroblasts relative to *Mfap5* ^*+/+*^ fibroblasts via the EnrichR package in R version 4.4.1 [44, 48, 49]. GO terms with a p.adj < 0.05 following Benjamini and Hochberg's approach for controlling the FDR were considered statistically significantly enriched, and only the top 25 significantly enriched terms based on p.adj were included in our analysis.

### 2.12 Isolation of Mfap5^+/+^ or Mfap5^−/−^ Neonatal Fibroblasts

At 2–4 days post‐birth, both male and female *Mfap5* ^*+/+*^ and *Mfap5* ^*−/−*^ neonatal mice were euthanized by ketamine and xylazine followed by cervical dislocation. Skin pelts were dissected from the torso and placed dermis side down in dispase (Worthington Biochemical Corp., final concentration 3.6 mg/mL) diluted in DMEM for 1 h at 37°C. Afterward, the epidermis was removed, and the dermis was minced into small pieces and digested with 0.5% (w/v) Collagenase type I (Sigma‐Aldrich) diluted in DMEM for 1 h at 37°C. The suspension was pipetted up and down gently to break up cell clumps, passed through a 40 μM cell strainer (ThermoFisher), and then rinsed with DMEM. The filtered suspension was then centrifuged for 5 min at 200 × g and the supernatant was removed. The cells were then resuspended in culture media, which consists of DMEM supplemented with 10% FBS and 100 Units/mL–100 μg/mL penicillin–streptomycin and cultured at 37°C in a humidified atmosphere of 5% CO_2_ and 95% air.

### 2.13 RNA Extraction, Library Preparation, and Data Processing for mRNA‐Sequencing of Mfap5^+/+^ and Mfap5^−/−^ Fibroblasts

We performed mRNA‐sequencing analysis to molecularly characterize the differences between *Mfap5* ^*+/+*^ and *Mfap5* ^*−/−*^ fibroblasts (Figure 5A). Isolated *Mfap5* ^*+/+*^ and *Mfap5* ^*−/−*^ neonatal fibroblasts were grown to confluency in multiple 6‐well plates. There were 3 biological replicates for each cell type and each sex at each time point, resulting in 12 samples. Total RNA was isolated using TRIzol (Invitrogen), purified with an RNA Clean & Concentrator‐25 kit (Zymo, Tustin, CA, USA), and treated with DNAse (ThermoFisher Scientific). RNA integrity numbers (RIN) were checked with Agilent Technologies 2100 Bioanalyzer (Agilent Technologies, Santa Clara, CA). All samples had an RIN between 8.9 and 9.4. RNA‐seq generated libraries were checked with Qubit and real‐time (RT) PCR for quantification and bioanalyzer for size distribution detection. Quantified libraries were pooled and sequenced on Illumina sequencing platforms (Illumina) and clean data was obtained by using in‐house perl scripts from Novogene (Novogene). Hisat2 (v2.0.5) was used to index and map reads to the mouse reference genome (GRCm39/mm39), and the mapped reads were assembled by StringTie (v1.3.3b) [50]. Raw counts of read numbers mapped to each gene were generated in R using the FeatureCounts package (v1.5.0‐p3) [51]. Library preparation, genome mapping, and raw gene counts for mRNA‐sequencing analysis were performed by Novogene (Novogene America).

All analyses described below were done by us using R version 4.4.1 with Bioconductor v3.20. Differential expression analysis of the raw count data generated by Novogene (Novogene America) was performed with the DESeq2 package (v1.20.0) in R [52]. Only genes with an average raw count of at least 10 across all samples were analyzed. *p*‐values were adjusted using the Benjamini and Hochberg's approach for controlling the false discovery rate (FDR) [52, 53]. Genes with an adjusted *p*‐value (p.adj) < 0.05 were assigned as being differentially expressed after DESeq2 analysis. Normalized count values for the DEGs in each sample were generated for each comparison. For data visualization and PCA, count data underwent variance‐stabilized transformation using the vst() function in DESeq2 and saved. PCA was then plotted using each sample's top 500 highly variable genes. In this study, we assessed differentially regulated genes in *Mfap5* ^*+/+*^ and *Mfap5* ^*−/−*^ fibroblasts at baseline using the following parameters: p.adj < 0.01 and log (fold change) > 0.25 or log(foldchange) < −0.25. Genes with a negative log(foldchange) were considered upregulated in *Mfap5* ^*+/+*^ fibroblasts, while genes with a positive log(fold change) were considered upregulated in *Mfap5* ^*−/−*^ fibroblasts. Z‐scores for genes corresponding to collagens were generated using their normalized count values in each sample and then plotted via the pheatmap() function in R [54]. GO BP and Reactome pathway term enrichment analyses for DEGs were performed as described previously for our single‐cell RNA‐sequencing analysis.

### 2.14 Analysis of Fibroblast Migration, Proliferation, and Gel Contraction

Fibroblasts were grown in culture media incubated at 37°C in an atmosphere of 5% CO_2_ and used once they reached 80%–90% confluence. All experiments were repeated 3–6 times and utilized male and female *Mfap5* ^*+/+*^ and *Mfap5* ^*−/−*^ fibroblasts.

Cell migration was assessed using an in vitro wounding model in which *Mfap5* ^*+/+*^ and *Mfap5* ^*−/−*^ fibroblasts were grown in a 12‐well plate until confluent and then treated with mitomycin‐C (1 μg/mL) (Sigma‐Aldrich) for 1 h to inhibit proliferation. In vitro wounds were created by performing a one‐by‐one cross‐scratch, which consisted of scraping the plate once horizontally and once vertically across with a 200 μL pipette tip. After wounding, cells were washed with PBS, and fresh media was added. Open areas for vertical and horizontal wounds were photographed at 0, 24, and 48 h after wounding and measured using ImageJ [31]. The migration rate is expressed as the percentage of the wound area closed at each time point. Representative images of wounds consisted only of vertical wounds.

Cell proliferation was assessed by seeding 3000 *Mfap5* ^*+/+*^ or *Mfap5* ^*−/−*^ fibroblasts into each well of a 96‐well plate. Proliferation was assessed at 24, 48, and 96 h post‐seeding using an MTS Cell Proliferation Assay Kit (Abcam, Waltham, MA, USA, Catalog # ab197010). Optical density (O.D.) values were read at an absorbance of 490 nm and recorded using a spectrophotometer (Molecular Devices, San Jose, CA, USA).

Cell migration and proliferation across different substrates were assessed by seeding *Mfap5* ^*+/+*^ or *Mfap5* ^*−/−*^ fibroblasts into 12‐ and 96‐well plates coated with rat tail collagen 1 (BD Biosciences) or mouse fibronectin (Innovative Research Inc., Novi, MI), at 5 μg/cm^2^. Migration and proliferation were assessed as described above.

Collagen contractility was assessed by embedding *Mfap5* ^*+/+*^ or *Mfap5* ^*−/−*^ fibroblasts in a collagen type I solution (BD Biosciences) containing fresh media and transferred to a 24‐well plate as described previously [3, 55]. Once solidified, the gel was released to form a free‐floating lattice structure. The gel was photographed at 0, 12, 24, 36, and 48 h. The gel area was measured using ImageJ [31]. Collagen contractility is expressed as the original collagen gel surface area's percent area for each time point.

### 2.15 In Vitro Preparation and Assessment of ECM Produced by Fibroblasts

Preparation of ECM produced by cultured *Mfap5* ^*+/+*^ or *Mfap5* ^*−/−*^ fibroblasts was performed using a previously published protocol [56]. In brief, fibroblasts were cultured until 100% confluency in either a 24‐well tissue culture‐treated glass bottom plate (Cellvis, Mountainview, CA, Catalog # P24‐1.5P) or a 24‐well tissue culture‐treated plastic plate and then switched to media supplemented with 100 μg/mL ascorbic acid (LabChem, Zelienople, PA). Half the growth media was replaced with an equivalent volume supplemented with 200 μg/mL ascorbic acid (LabChem) every other day. Fibroblasts and their deposited ECM were used after 7 days of ascorbic acid treatment.

For immunocytochemistry analysis of deposited COL1A1 and COL3A1, fibroblasts were washed with PBS, fixed by incubation with 4% paraformaldehyde (PFA) (Sigma‐Aldrich) in PBS at room temperature for 20 min, and then washed with PBS before being stored at 4°C until staining. Fixed fibroblasts were blocked and permeabilized with 5% normal donkey serum (Jackson ImmunoResearch Labs, West Grove, PA) and 1% Tween‐20 for 2–3 h at room temperature. Afterward, cells were incubated with goat anti‐type 1 collagen (SouthernBiotech, Birmingham, AL) and rabbit anti‐type 3 collagen (Proteintech, Rosemont, IL) in blocking/permeabilization buffer at a 1:250 dilution overnight at 4°C. Samples were then washed with PBS and incubated with Alexa Fluor 594 donkey anti‐goat IgG (Thermofisher) and Alexa Fluor 488 donkey anti‐rabbit IgG (Thermofisher) in blocking/permeabilization buffer at a 1:1000 dilution for 1 h at room temperature. Samples were washed with PBS and incubated with DAPI (Sigma‐Aldrich) for 2 min for nuclear counterstaining. DAPI was removed, and fresh PBS was added. Z‐section images were taken at 20× magnification using a Zeiss LSM 710 Confocal Microscope (Zeiss). Images were then processed with Fiji ImageJ, and an assessment of ECM pattern and organization was performed using TWOMBLI [31, 36].

For relative quantification of total cell extracts, which includes deposited and cellular collagens in the ECM, 2× laemmli sample buffer supplemented with 1× Dithiothreitol (DTT) (Bio‐Rad Laboratories, Hercules, CA) was added to in vitro ECM preparations. Wells were scraped, and suspensions were heated at 95°C using a heat block for 10 min. Afterward, samples were centrifuged for 3 min at 800 × g and sheared using a 1 mL syringe with a 26G needle until viscosity was reduced. Samples were then heated at 95°C for 10 min and centrifuged again. Samples were stored at −80°C until use in western blot analysis. Total cell extract samples were loaded and separated in 4%–20% Mini‐PROTEAN TGX Precast gels (Bio‐Rad Laboratories), transferred to PVDF membranes using the Trans‐Blot Turbo Transfer System (Bio‐Rad Laboratories) and blocked with 5% non‐fat skim milk in TBST (0.1% Tween). Subsequently, membranes were incubated with rabbit anti‐ß‐actin antibodies (Cell Signaling Technology) at 1:1000 dilution and rabbit anti‐COL1A1 (Cell Signaling Technology) at 1:500 dilution or rabbit anti‐type 3 collagen (ProteinTech) at 1:100 dilution overnight at 4°C. Blots were then incubated with anti‐rabbit Horseradish Peroxidase‐linked IgG secondary antibody (Bio‐Rad Laboratories) at 1:2000 dilution for 1.5 h at room temperature. Immunoreactive bands were visualized with Clarity Western ECL Substrate (Bio‐Rad Laboratories). Protein bands were detected using the ChemiDoc XRS Imaging System (Bio‐Rad Laboratories). The Quantity One Analysis Software (Bio‐Rad Laboratories) was used to quantify band intensities of western blot images. COL1A1 and COL3A1 protein band intensities were normalized to the housekeeping protein ß‐actin. Relative protein expression is presented with respect to *Mfap5* ^*+/+*^ fibroblasts. The concentration, dilution, and incubation conditions for the antibodies used are shown in Table 1.

### 2.16 Statistical Analysis

Data were expressed as mean ± standard deviation (SD), and their normality was evaluated using the Kolmogorov–Smirnov or Shapiro–Wilk tests. The number of mice for each skin wound model or samples needed for our in vitro assays was identified by power analysis based on standard deviations from previous studies. Statistical comparisons were performed using a two‐tailed unpaired t‐test with Welch's correction or a two‐way ANOVA followed by a two‐stage linear step‐up procedure of Benjamini, Krieger, and Yekutieli posthoc testing using GraphPad Prism version 8.0 (GraphPad, San Diego, CA). Outliers were removed by Grub's test with an alpha significance level of 0.05. *p*‐Values less than 0.05 were considered statistically significant.

## 3 Results

### 3.1 Loss of MFAP5 Reduces Wound Re‐Epithelialization and Angiogenesis While Enhancing Wound Inflammatory Cell Influx

Using an excisional skin wound model, we found that external wounds were larger in *Mfap5* ^*−/−*^ mice on days 1 and 5 post‐wounding (Figure 1A,B) and that there was reduced re‐epithelialization in *Mfap5* ^*−/−*^ mice on day 3 post‐wounding (Figure 1C–E). Notably, wounds in *Mfap5* ^*−/−*^ and *Mfap5* ^*+/+*^ mice closed and re‐epithelialized at the same time by Day 7. *Mfap5* ^*−/−*^ mice also had reduced angiogenesis at day 7 post‐wounding compared to *Mfap5* ^*+/+*^ mice (Figure 1F,G), though this difference was resolved by day 14 post‐wounding (Figure 1F,G). There was no significant difference in the vascular density of normal skin (NS) between *Mfap5* ^*−/−*^ and *Mfap5* ^*+/+*^ mice.

Since MFAP5 is an ECM protein that could affect wound cell migration, we next examined whether the loss of MFAP5 could alter wound inflammatory cell influx [5, 28, 57, 58]. At Day 3 post‐wounding, *Mfap5* ^*−/−*^ mice had significantly increased neutrophil and macrophage presence in the wound compared to control *Mfap5* ^*+/+*^ mice (Figure 1H–K). Altogether, our results suggest that MFAP5 may have a role in regulating wound closure, angiogenesis, and inflammatory cell influx.

### 3.2 Loss of MFAP5 Reduces Collagen Deposition and Composition in NS but Not in Wounds

**FIGURE 2:**
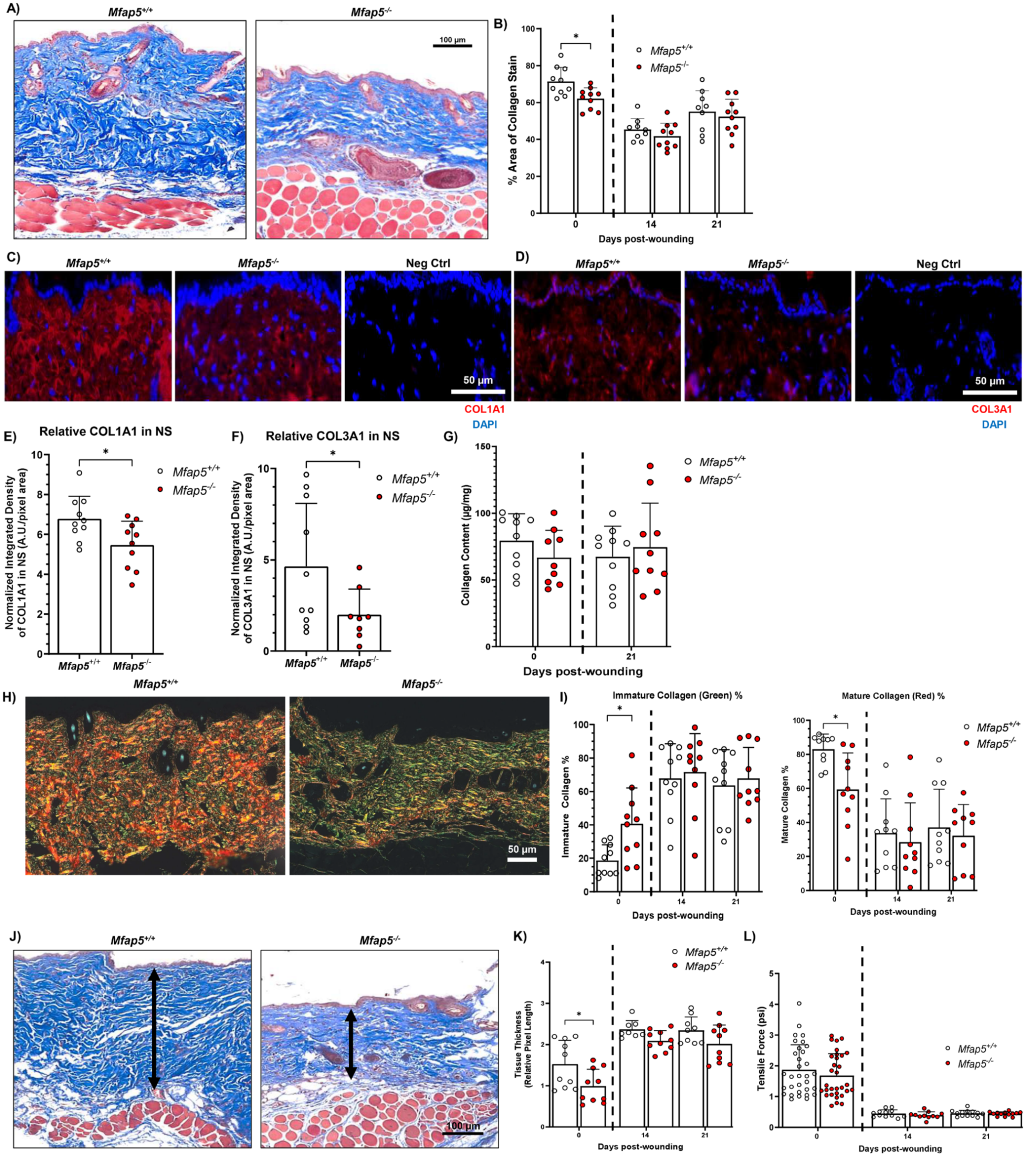
Loss of MFAP5 reduces collagen deposition and alters skin dermis collagen maturity in vivo. (A) Cropped representative photomicrographs of Masson's trichrome staining in normal skin (NS) from *Mfap5* ^*+/+*^ and *Mfap5* ^*−/−*^ mouse taken at 10× magnification. (B) Quantitative collagen deposition in NS and in tissue from Days 14‐, and 21‐post‐wounding expressed as percent area‐stained blue in the dermis or the wound bed. Cropped representative photomicrographs of fluorescence microscopy images of COL1A1 (red) (C) and COL3A1 (red) (D) staining in NS tissue of *Mfap5* ^*+/+*^ and *Mfap5* ^*−/−*^ mice taken at 20× magnification. Nuclei in blue (DAPI). COL1A1 or COL3A1 content in normal skin dermis of *Mfap5* ^*+/+*^ and *Mfap5* ^*−/−*^ mice expressed as arbitrary units of COL1A1 (E) or COL3A1 (F) fluorescence relative to dermis area. (G) Hydroxyproline assay quantification of collagen content in NS and in tissue from Day 21‐post wounding expressed as μg collagen per mg total tissue weight. (H) Representative photomicrographs of Picrosirius red staining of normal *Mfap5* ^*+/+*^ and *Mfap5* ^*−/−*^ mouse skin taken at 20× magnification. (I) Quantitative collagen maturity of NS and tissue from Days 14‐ and 21‐post wounding expressed as the percent of immature (green) and mature (red) collagen content in the dermis or wound bed. (J) Cropped representative photomicrographs of dermis width in NS of *Mfap5* ^*+/+*^ and *Mfap5* ^*−/−*^ mice were taken at 10× magnification. Quantification of dermis thickness (K) and tensile strength (L) in NS and tissue from Days 14‐ and 21‐post wounding. *N* = 9–10 for B, C, E, G, and I. *N* = 11–32 for J. Bars on all graphs indicate mean ± SD. *p < 0.05. Two‐way ANOVA with two‐stage linear step‐up procedure of Benjamini, Krieger, and Yekutieli (vs *Mfap5* ^*+/+*^) was used for B, G, I, K, and L. Two‐tailed unpaired *t*‐test with Welch's correction was used for E and F.

Masson's Trichrome and Picrosirius Red staining analyses were performed to evaluate how loss of MFAP5 in *Mfap5* ^*−/−*^ mice affects collagen deposition and composition in NS and healing skin wounds. NS of *Mfap5* ^*−/−*^ mice exhibited a significant decrease in collagen content compared to the NS of *Mfap5* ^*+/+*^ mice, but no differences were observed in wounds between *Mfap5* ^*−/−*^ and *Mfap5* ^*+/+*^ mice (Figure 2A,B). To validate the findings from Masson's Trichrome staining, we performed indirect immunofluorescence staining for COL1A1 and COL3A1 in NS of *Mfap5* ^*+/+*^ and *Mfap5* ^*−/−*^ mice and a hydroxyproline assay on NS and wound tissue from 21 days post‐wounding. Immunofluorescence staining found significantly less COL1A1 and COL3A1 in the NS of *Mfap5* ^*−/−*^ mice as compared to *Mfap5* ^*+/+*^ mice (Figure 2C–F), while measurement of hydroxyproline content showed a similar trend to our Masson's Trichrome and immunofluorescent staining—decreased collagen content in the NS of *Mfap5* ^*−/−*^ mice but not in wounds (Figure 2A–G). *Mfap5* ^*−/−*^ mice NS also had significantly more immature and less mature collagen than *Mfap5* ^*+/+*^ mice NS (Figure 2H,I). Since NS of *Mfap5* ^*−/−*^ mice exhibited the most differences as compared to *Mfap5* ^*+/+*^ mice, we performed TWOMBLI analysis to assess whether collagen organization in the NS of *Mfap5* ^*−/−*^ mice was altered. There were no significant differences in any of the TWOMBLI parameters assessed (Figure S1).

The dermis thickness and tensile strength of NS and wounds from *Mfap5* ^*+/+*^ and *Mfap5* ^−/−^ mice were measured as a functional correlate. The dermis of *Mfap5* ^*−/−*^ mice NS was significantly thinner than that of *Mfap5* ^+/+^ mice NS, while wound dermis width was not significantly different (Figure 2J,K). No statistically significant differences were observed in the wound breaking strength of either the NS or wounds of *Mfap5* ^*−/−*^ and *Mfap5* ^*+/+*^ mice (Figure 2L). Our data suggest that MFAP5 is important for collagen deposition, but not organization, in NS.

### 3.3 Single‐Cell RNA‐Sequencing Reveals Distinct Fibroblast Populations

To determine the impact that loss of MFAP5 has on fibroblast subsets, we performed single‐cell RNA‐sequencing on unsorted cells from NS (D0) and wounds at D3 and D7 post‐wounding from male and female *Mfap5* ^*+/+*^ and *Mfap5* ^*−/−*^ mice. Data quality control was performed for each sample independently, and 72 876 sequenced cells met our quality control metrics. Each cell had ≥ 200 genes, mitochondrial gene expression < 10%, and a multiplet likelihood score < 0.25. Genes also had to be present in ≥ 10 cells. Post‐quality control single‐cell RNA‐sequencing data for each sample was then integrated into a single dataset. Unsupervised clustering of the integrated dataset using the Seurat package identified 18 cell clusters. Input of the differentially expressed marker genes (p.adj < 0.05, Log2FC > 2, pct.1 > 0.40, and pct.2 < 0.20) found in each cell cluster into EnrichR was done to identify putative identities of each cluster (Figure S2A,B) [40]. Figure S2C shows the feature plot for select differentially expressed markers for each cell identity. Cell type proportions over time are shown in Figure S2D and Table S4. Fibroblasts were highly enriched for collagens, including Col1a1, Col3a1, Col5a1, and ECM proteins, including *Dcn*, *Mfap5*, and *Fbln2*. Fibroblasts were also the only cell population that highly expressed *Mfap5*, suggesting they are the primary producers of MFAP5.

**FIGURE 3:**
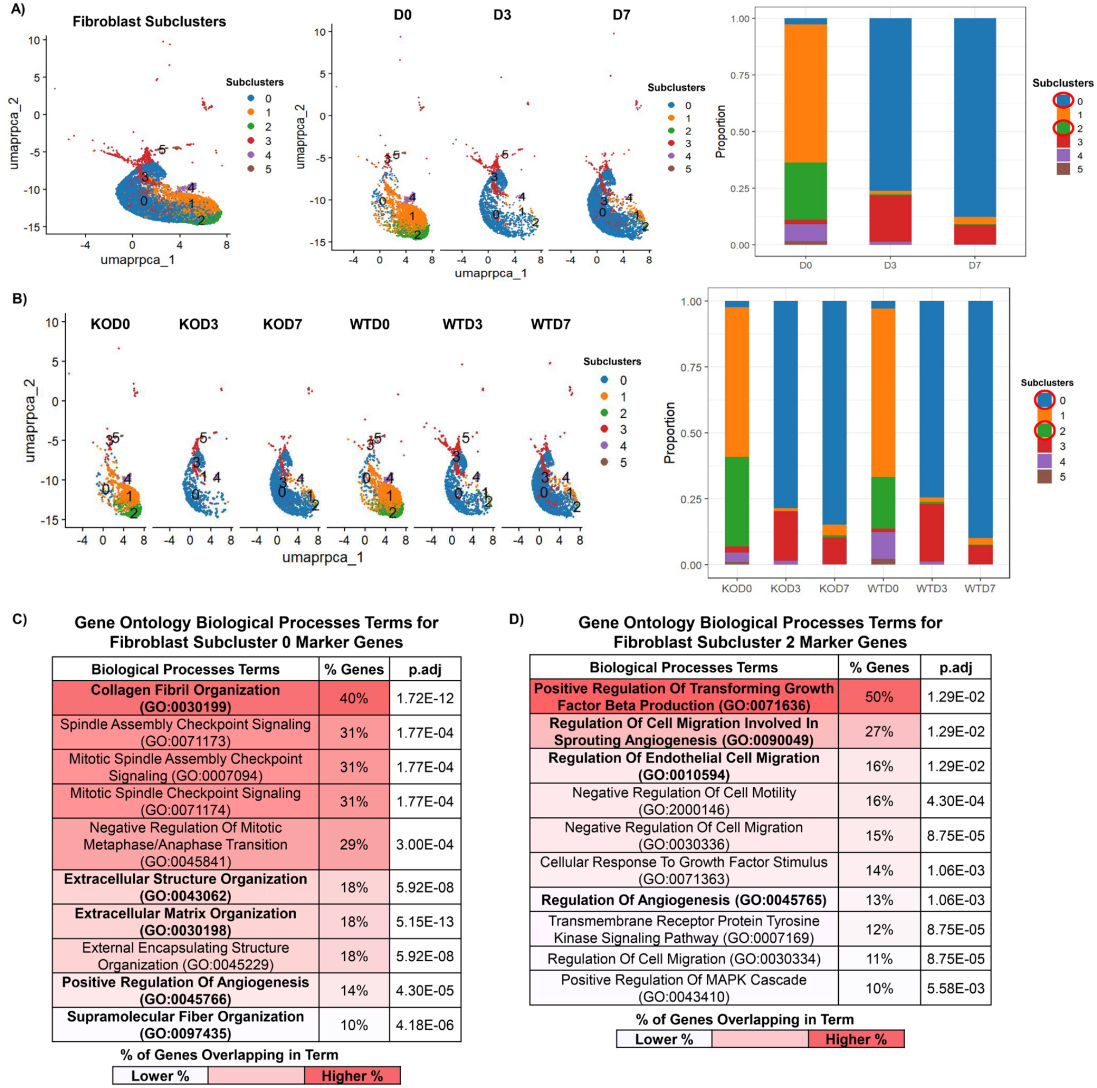
Fibroblasts differentially expressing *Mfap5* in vivo have biological functions related to ECM synthesis and organization as well as angiogenesis. Relative fibroblast subcluster composition arranged by time post‐wounding (A) and experimental group (B). Top 10 significantly enriched gene ontology biological processes (GO BP) terms for top differentially expressed marker genes in fibroblast subcluster 0 (C) and fibroblast subcluster 2 (D). Terms of interest are bolded for emphasis. Adjusted *p*‐values for GO BP and Reactome pathway terms were determined by the Benjamini‐Hochberg method.

Next, we performed unsupervised subclustering of the fibroblast cell population, which comprises fibroblasts from both *Mfap5* ^*+/+*^ and *Mfap5* ^*−/−*^ mice. Our analysis yielded 6 unique clusters, C0–C5 (Figure 3A). These fibroblast clusters were separated by time post‐wounding and experimental group (Figure 3B,C). Fibroblast cluster proportions over time are summarized in Table S5. We found that *Mfap5* ^*+/+*^ and *Mfap5* ^*−/−*^ mouse fibroblasts cluster similarly, as each fibroblast cluster identified in our analyses was comprised of fibroblasts from both *Mfap5* ^*+/+*^ and *Mfap5* ^*−/−*^ mice. We also observed that fibroblast cluster proportions for *Mfap5* ^*+/+*^ and *Mfap5* ^*−/−*^ mice exhibit similar changes during wound healing (Figure 3C). This suggests that the loss of MFAP5 did not significantly alter fibroblast subpopulations or their marker genes.

### 3.4 Fibroblast Subclusters That More Highly Express MFAP5 Are Likely Involved in ECM Deposition and Organization

We performed differential marker gene analysis to determine which subcluster(s) differentially expressed MFAP5. For *Mfap5* ^*+/+*^ and *Mfap5* ^*−/−*^ mice, Cluster 0 and Cluster 2 fibroblasts had MFAP5 as a DEG. Cluster 2 was more prevalent in NS than Cluster 0 (Figure 3B,C). However, the proportion of Cluster 0 fibroblasts increases as wound healing progresses, while Cluster 2 decreases (Figure 3B,C). Eventually, Cluster 0 fibroblasts become the dominant wound fibroblast cluster (Figure 3B,C).

We performed GO BP and Reactome pathway enrichment analysis using DEGs to infer the biological functions of these fibroblast subclusters. The top 25 significantly enriched BP GO and Reactome pathway terms for Cluster 0 and 2 fibroblasts, based on their p.adj value and each term's annotated genes, are listed in Tables S6–S9. The top 10 significant GO BP terms enriched for Cluster 0 and 2 fibroblasts, ranked by the percentage of genes annotated to the term's gene set, are shown in Figure 3D,E. Cluster 0 and 2 fibroblasts had terms relating to ECM organization, ECM synthesis, and angiogenesis (Figure 3D and Tables S6 and S7). Cluster 2 fibroblasts also had terms relating to epithelial migration (Figure 3E and Tables S8 and S9). Our analysis validates previous work identifying fibroblasts as a major source of MFAP5 in healing wounds and suggests that fibroblasts that more highly express *Mfap5* are likely to be important contributors to ECM deposition during wound healing, as well as angiogenesis [4].

### 3.5 Loss of MFAP5 Significantly Alters Fibroblast Transcriptome

**FIGURE 4:**
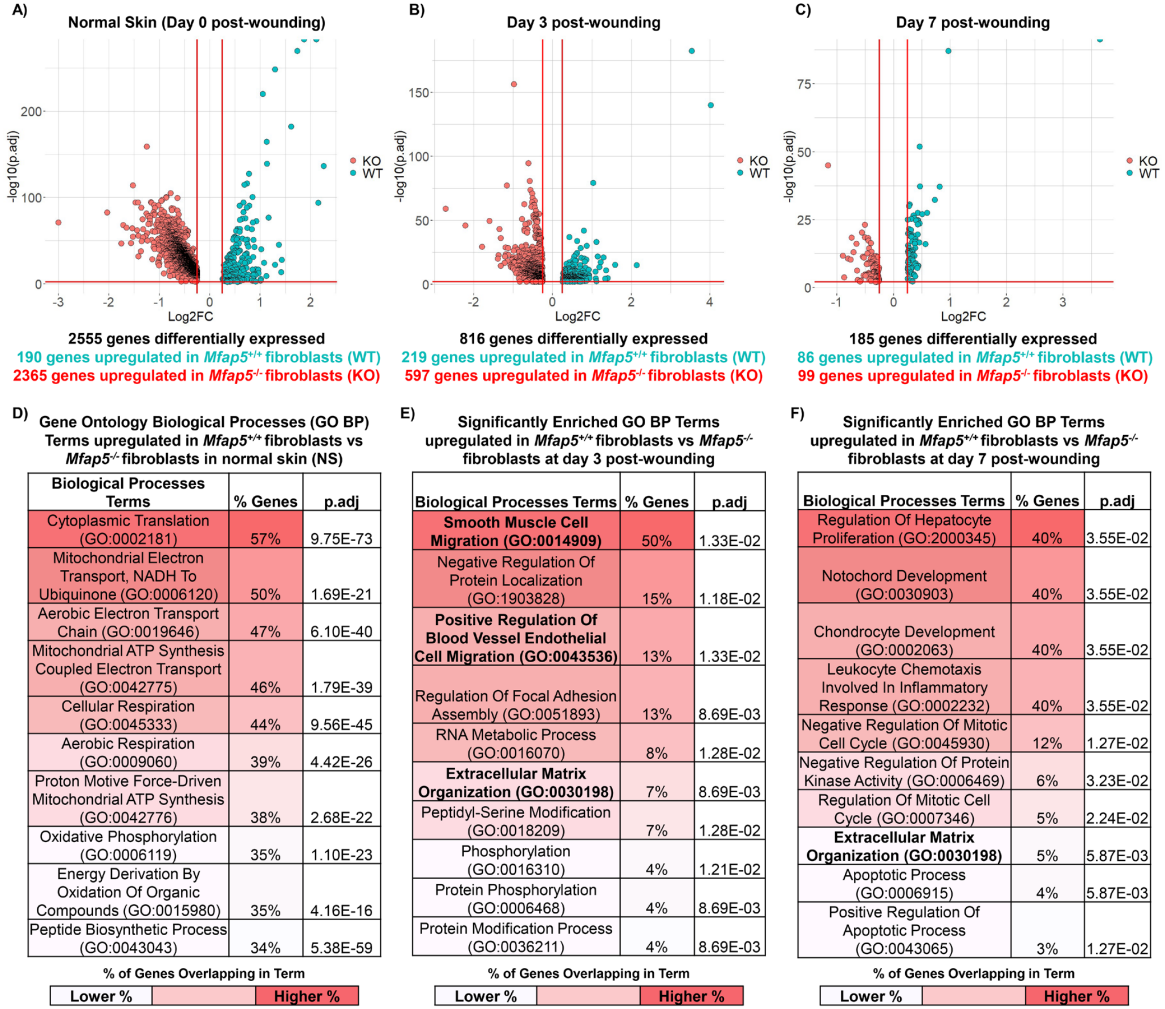
Loss of MFAP5 in vivo alters fibroblast transcriptome and inhibits functions related to ECM synthesis and angiogenesis. Differential gene expression analysis was performed between single‐cell RNA‐sequencing identified *Mfap5* ^*+/+*^ and *Mfap5* ^*−/−*^ mouse fibroblasts at each time point using the FindAllMarkers() function in R. Volcano plot showing the number of differentially expressed genes (DEGs) between *Mfap5* ^*+/+*^ and *Mfap5* ^*−/−*^ mouse fibroblasts in normal skin (NS) (A), at Day 3 post‐wounding (B), and at day 7 post‐wounding (C). The top 10 significantly enriched gene ontology biological processes (GO BP) terms for *Mfap5* ^*+/+*^ fibroblasts vs. *Mfap5* ^*−/−*^ fibroblasts in NS (D), at Day 3 post‐wounding (E), and at day 7 post‐wounding (F). Terms of interest are bolded for emphasis. Adjusted *p*‐values for GO BP terms were determined by the Benjamini‐Hochberg method.

To determine the effects of loss of MFAP5 on the fibroblast transcriptome during wound healing, we performed DEG analysis between *Mfap5* ^*+/+*^ and *Mfap5* ^*−/−*^ mice fibroblasts at each time point using the FindAllMarkers() function in R. Genes with a positive Log2FC are upregulated in *Mfap5* ^*+/+*^ fibroblasts, and genes with a negative Log2FC are downregulated in *Mfap5* ^*−/−*^ fibroblasts. The total number of DEGs and their directionality are shown in Figure 4A–C and Table 2. We performed GO BP and Reactome pathway term enrichment to infer the biological significance of these DEGs. The top 25 significantly enriched GO BP and Reactome pathway terms for *Mfap5* ^*+/+*^ or *Mfap5* ^*−/−*^ fibroblasts in NS (D0), D3, and D7, and each term's annotated genes are listed in Tables S10–S21. The top 10 significantly enriched GO BP terms for DEGs in *Mfap5* ^*+/+*^ fibroblasts in NS (D0), D3, and D7 are shown in Figure 4D–F. Angiogenesis, VEGF signaling, and ECM synthesis and organization terms were enriched for *Mfap5* ^*+/+*^ fibroblasts at D3 and D7, suggesting these processes may be downregulated in *Mfap5* ^*−/−*^ fibroblasts during healing (Figure 4E,F, and Tables S14, S15, S18, and S19). There were also terms relating to epithelial cell function, activation, and smooth muscle migration (Tables S14 and S18). Interestingly, the DEGs upregulated in *Mfap5* ^*−/−*^ fibroblasts vs. *Mfap5* ^*+/+*^ fibroblasts from NS and wounds related to inflammation (Table S13).

### 3.6 MFAP5 Regulates Fibroblast Transcriptome and Processes Related to Wound Healing

The effects that the loss of MFAP5 has on the fibroblast transcriptome may be obscured during in vivo healing, as a myriad of cell–cell, cell‐ECM, and cell‐cytokine interactions may compensate for the loss of MFAP5. Thus, to assess intrinsic changes to the fibroblast transcriptome and infer mechanisms for the functional deficits observed following the loss of MFAP5, we performed mRNA‐sequencing on *Mfap5* ^*+/+*^ and *Mfap5* ^*−/−*^ fibroblasts cultured in vitro.

Visualization of the normalized data by PCA showed that the first two PCs account for 26.3% and 64.3% of the observed variance in the dataset, respectively, and that these components distinguish the samples by cell type (Figure S3). We performed differential expression analysis to assess how MFAP5 directly influences the basal fibroblast transcriptome. Genes with a positive Log2FC are upregulated in *Mfap5* ^*−/−*^ fibroblasts, while genes with a negative Log2FC are downregulated in *Mfap5* ^*−/−*^ fibroblasts. GO BP and Reactome pathway term enrichment analysis was performed to confer the biological significance of these DEGs.

**FIGURE 5:**
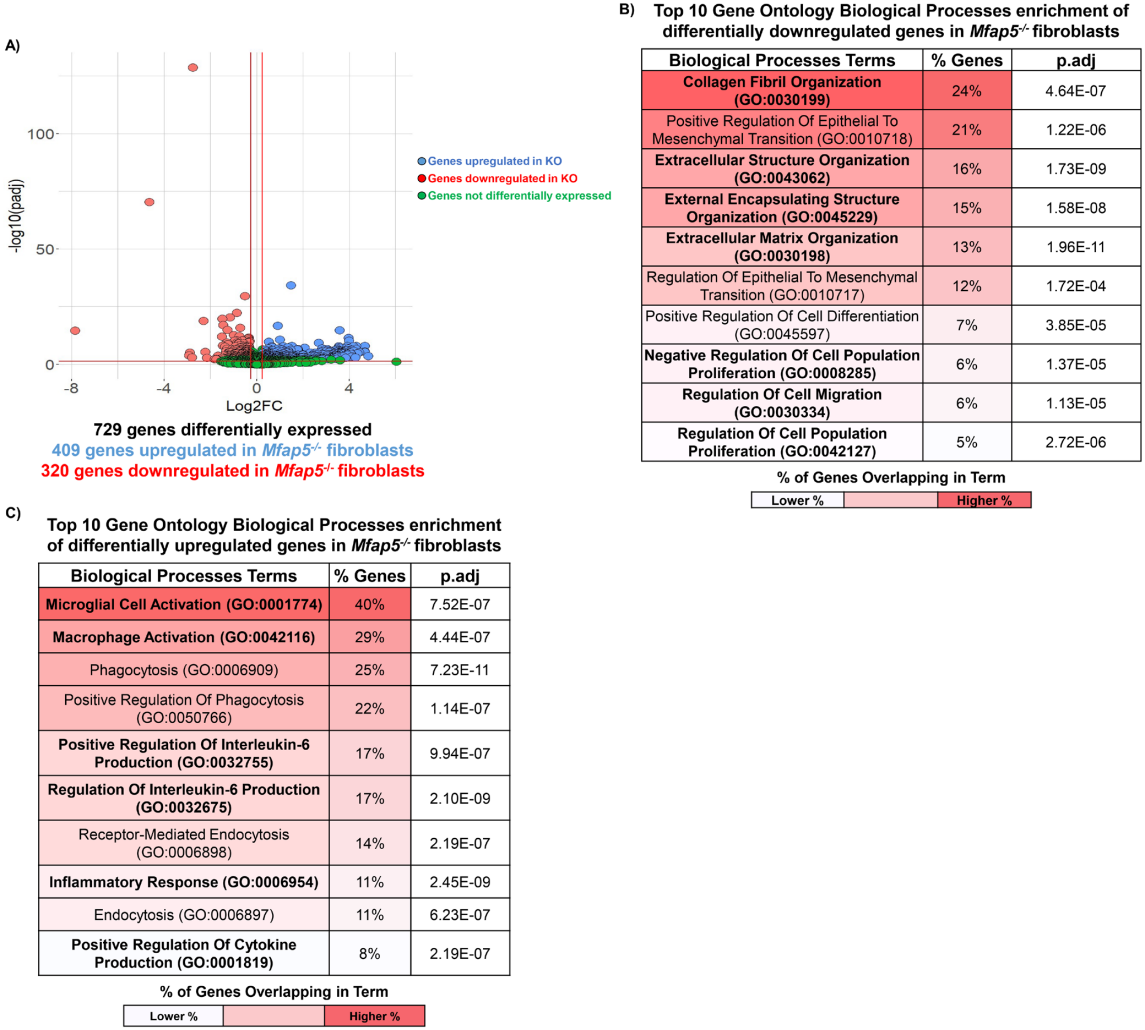
Loss of MFAP5 in vitro inhibits fibroblast functions related to ECM synthesis and organization, immune regulation, and angiogenesis. (A) Volcano plot for the differentially expressed genes in *Mfap5* ^*−/−*^ fibroblasts relative to *Mfap5* ^*+/+*^ fibroblasts in vitro. Top 10 significantly enriched gene ontology biological processes (GO BP) for differentially downregulated genes in *Mfap5* ^*−/−*^ fibroblasts (B) and differentially upregulated genes in *Mfap5* ^*−/−*^ fibroblasts (C) relative to *Mfap5* ^*+/+*^ fibroblasts. Terms of interest are bolded for emphasis. Adjusted *p*‐values for GO BP terms were determined by the Benjamini‐Hochberg method.

The top 25 significantly enriched GO BP and Reactome pathway terms based on their p.adj value for *Mfap5* ^*+/+*^ or *Mfap5* ^*−/−*^ fibroblasts and each term's gene annotations are listed in Tables S22–S25. The top 10 significantly enriched BP terms for DEGs in *Mfap5* ^*+/+*^ or *Mfap5* ^*−/−*^ fibroblasts, ranked by the percentage of genes annotated to each term's gene set, are shown in Figure 5C,D. In *Mfap5* ^*+/+*^ fibroblasts, terms related to ECM synthesis and organization, cell migration and proliferation, and to TGFß and Notch signaling (Figure 5C and Tables S22 and S23). *Mfap5* ^*−/−*^ fibroblasts had terms related to inflammation (Figure 5D and Tables S24 and S25). Our enrichment analyses indicate that *Mfap5* ^*−/−*^ fibroblasts have functional inhibitions related to scar formation, with upregulation of genes relating to inflammation.

**TABLE 2:** Differentially expressed genes (DEGs) between *Mfap5* ^+/+^ and *Mfap5* ^−/−^ fibroblasts at each time point following FindAllMarker analysis.

Day post‐wounding	Upregulated in *Mfap5* ^+/+^ mouse fibroblasts	Upregulated in *Mfap5* ^−/−^ mouse fibroblasts	Total number of DEGs
0	271	1904	2175
3	306	664	970
7	147	121	26

### 3.7 MFAP5 Regulates In Vitro Fibroblast Functions Related to Scar Formation

**FIGURE 6:**
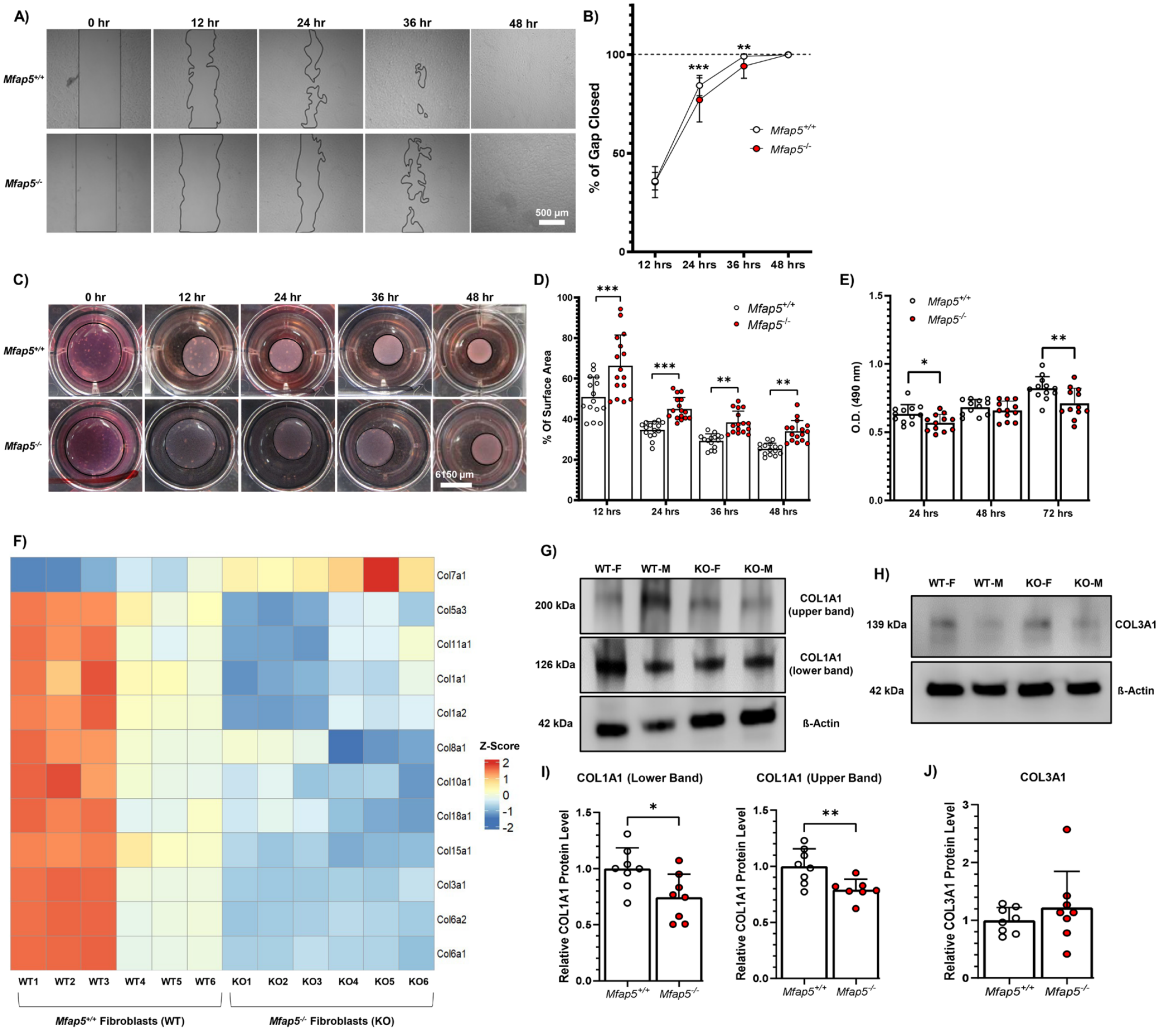
Loss of MFAP5 inhibits fibroblast phenotype related to scar formation in vitro. *Mfap5* ^*+/+*^ and *Mfap5* ^*−/−*^ fibroblasts underwent functional assays assessing features related to scar formation in vitro. (A) Representative photos of in vitro vertical wounds after one‐by‐one cross‐scratching for the *Mfap5* ^*+/+*^ and *Mfap5* ^*−/−*^ fibroblast migration assay. Areas not covered by cells are outlined by a black line. Rate of cell migration, expressed as a percent of wound closed (B) at each time point. *N* = 19–20. (C) Representative photos of the collagen gel contraction assay. Gel area is depicted by a black line. Scale bar = 6150 μm. (D) Rate of gel contraction, expressed as a percentage of the original gel surface area. *N* = 15–16. (E) MTS proliferation assay was performed on *Mfap5* ^*+/+*^ and *Mfap5* ^*−/−*^ fibroblasts at 24‐, 48‐, and 72‐h post‐seeding. *N* = 12, each dot representing a biological replicate consisting of 4–6 technical replicates. (F) Heatmap of differentially expressed collagens between *Mfap5* ^*+/+*^ (WT) and *Mfap5* ^*−/−*^ (KO) fibroblasts. Cropped representative immunoblots for COL1A1 and ß‐Actin (G) or COL3A1 and ß‐Actin (H) in total cell extract (TCE) from *Mfap5* ^*+/+*^ and *Mfap5* ^*−/−*^ fibroblasts. Relative band density of COL1A1 (I) and COL3A1 (J) to ß‐Actin from western blot analysis performed on TCE from *Mfap5* ^*+/+*^ and *Mfap5* ^*−/−*^ fibroblasts. *N* = 7–8. Bars on all graphs indicate mean ± SD. *p < 0.05, **p < 0.01, ***p < 0.0001. Two‐way ANOVA with two‐stage linear step‐up procedure of Benjamini, Krieger and Yekutieli post hoc testing (vs *Mfap5* ^*+/+*^) was used for C, E, and F. Two‐tailed unpaired t‐test with Welch's correction was used for I and J.

To examine the direct effects of MFAP5 on fibroblast function, we isolated and cultured skin fibroblasts from neonatal *Mfap5* ^*+/+*^ and *Mfap5* ^*−/−*^ mice for in vitro assays assessing fibroblast functions linked to scar formation, including cell migration and proliferation, contractility, and ECM deposition (Figure 6A). *Mfap5* ^*−/−*^ fibroblasts had significantly decreased cell migration rates at 24‐ and 36‐h post‐wounding compared to *Mfap5* ^*+/+*^ fibroblasts (Figure 6A,B). *Mfap5* ^*−/−*^ fibroblasts also had significantly reduced rates of collagen contractility and cell proliferation compared to *Mfap5* ^*+/+*^ fibroblasts for all time points assessed (Figure 6C–E). To determine if *Mfap5* ^*−/−*^ fibroblasts produced less ECM components, specifically collagens, we identified differentially expressed collagens between *Mfap5* ^*+/+*^ and *Mfap5* ^*−/−*^ fibroblasts (Figure 6F). Collagen types 1 and 3 were downregulated in *Mfap5* ^*−/−*^ fibroblasts compared to *Mfap5* ^*+/+*^ fibroblasts. Western blot analysis found that *Mfap5* ^*−/−*^ fibroblasts produced less COL1A1, while COL3A1 levels were similar between *Mfap5* ^*+/+*^ and *Mfap5* ^*−/−*^ fibroblasts (Figure 6G–J). TWOMBLI analysis shows that the organization of deposited COL1A1 and COL3A1 by *Mfap5* ^*−/−*^ and *Mfap5* ^*+/+*^ fibroblasts differs significantly (Figure S4). Our data validate and expand upon the notion that MFAP5 enhances fibroblast functions related to scar formation.

### 3.8 Functional Deficits of Mfap5^−/−^ Fibroblasts Persists Across Varying ECM Proteins

**FIGURE 7:**
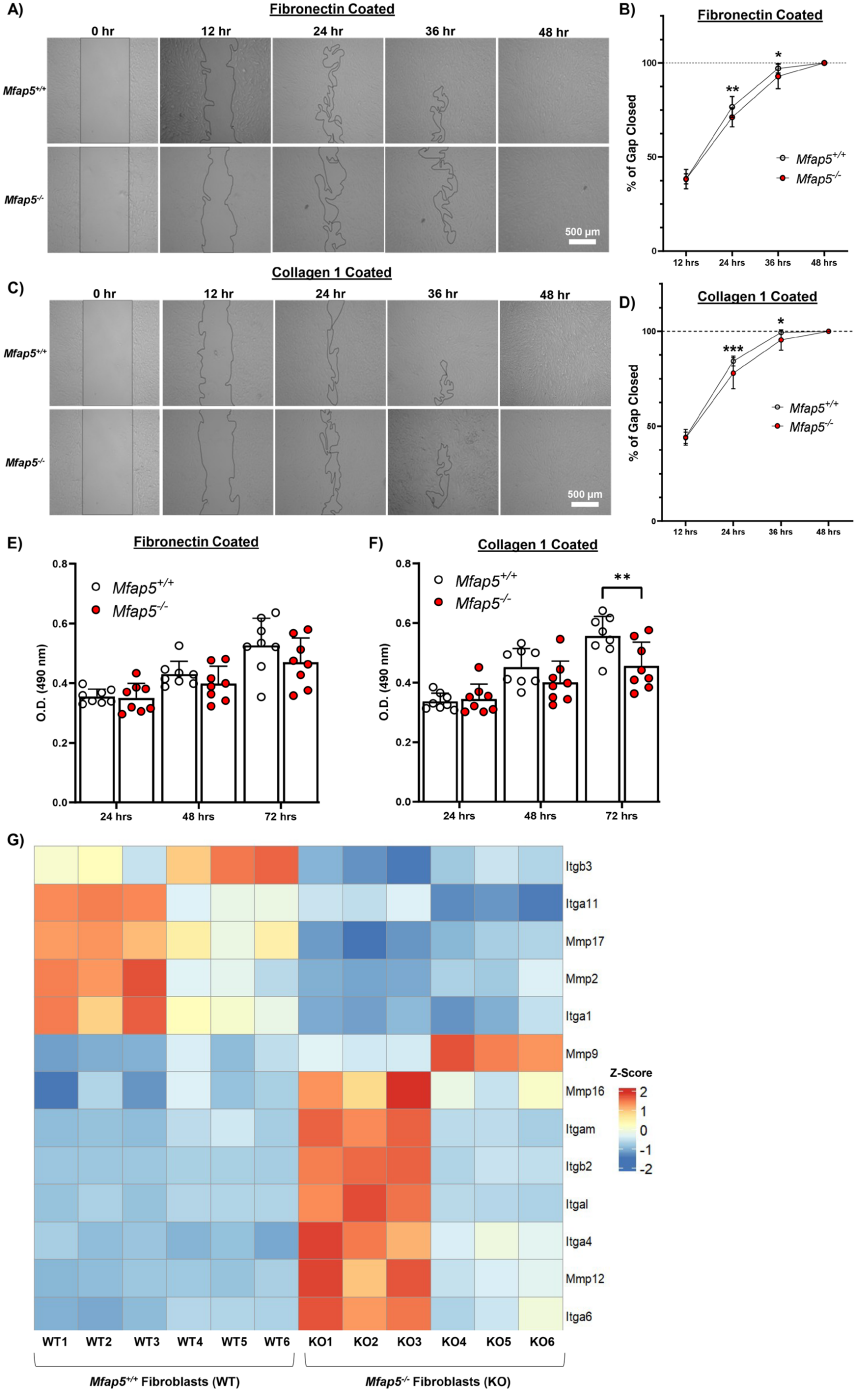
*Mfap5* ^*−/−*^ fibroblasts retain their inhibited migratory and proliferative capacity across different ECM substrates. In vitro vertical wounds after one‐by‐one cross‐scratching for *Mfap5* ^*+/+*^ and *Mfap5* ^*−/−*^ fibroblast migration assay on fibronectin (A) coated plates expressed as a percent of the wound closed (B) at each time point. *Mfap5* ^*+/+*^ and *Mfap5* ^*−/−*^ fibroblast migration assay on collagen I (C) coated plates expressed as a percent of the wound closed (D). *N* = 12 for B and D. Areas not covered by cells are outlined by a black line. MTS proliferation assay was performed on *Mfap5* ^*+/+*^ and *Mfap5* ^*−/−*^ fibroblasts at 24‐, 48‐, and 72‐h post‐seeding onto a fibronectin (E) or collagen I (F) coated plate. *N* = 8 for E and F, with each dot representing a biological replicate that consists of 4–6 technical replicates. (G) Heatmap of differentially expressed matrix metalloproteinases (*Mmp*) and integrins (*Itg*) between *Mfap5* ^*+/+*^ (WT) and *Mfap5* ^*−/−*^ (KO) fibroblasts. Bars on all graphs indicate mean ± SD. *p < 0.05, **p < 0.01, ***p < 0.001. Two‐way ANOVA with two‐stage linear step‐up procedure of Benjamini, Krieger, and Yekutieli post hoc testing (vs *Mfap5* ^*+/+*^).

To better recapitulate the ECM interactions that skin fibroblasts exhibit in tissue wound healing, *Mfap5* ^*−/−*^ and *Mfap5* ^*+/+*^ fibroblasts were cultured on plates coated with fibronectin and collagen I and examined in a migration or proliferation assay. Consistent with results in non‐coated plates, *Mfap5* ^*−/−*^ fibroblasts exhibited delayed cell migration and proliferation over varying substrates compared to *Mfap5* ^*+/+*^ fibroblasts (Figure 7A–D). Given the importance of integrins and MMPs in facilitating cell migration across ECM components, we investigated whether *Mfap5* ^*+/+*^ and *Mfap5* ^*−/−*^ fibroblasts exhibit differential expression of MMPs and integrins [59, 60, 61]. *Mfap5* ^*−/−*^ fibroblasts showed a downregulation of integrins‐α_1_ (*Itga1*), −α_11_ (*Itga11*), −ß_3_ (*Itgb3*), *Mmp2*, and *Mmp17* (Figure 7G) compared to *Mfap5* ^*+/+*^ fibroblasts. *Mfap5* ^*−/−*^ fibroblasts showed upregulation of integrins‐α_4_ (*Itga4*), −α_6_ (*Itga6*), −α_L_ (*Itgal*), −α_M_ (*Itgam*), −ß_2_ (*Itgb2*), *Mmp9, Mmp12*, and *Mmp16* (Figure 7I) compared to *Mfap5* ^*+/+*^ fibroblasts. Thus, *Mfap5* ^*−/−*^ fibroblasts may have deficits in cell adhesion and ECM remodeling, which may underlie the retained inhibition of *Mfap5* ^*−/−*^ fibroblast migration and proliferation across ECM components. Our results might also suggest that MFAP5 supports specific integrin‐mediated signaling pathways.

## 4 Discussion

A key feature of scar formation in wound healing is fibroblast activation. Once activated, fibroblasts generate ECM and contractile forces, which ultimately lead to the formation of a scar [62, 63, 64]. Numerous factors and mechanisms contribute to fibroblast activation and scar formation during wound healing. Here, we dissect how the loss of MFAP5 affects skin wound healing in vivo and fibroblast function in vitro to infer its functions.

External wound closure was delayed in MFAP5‐deficient mice only during early wound healing. Given the early time frame, this finding is most likely a result of altered wound contraction. Regarding deficits in contraction, *Mfap5* ^−/−^ fibroblasts exhibit reduced contractility in an in vitro assay. *Acta2* was significantly downregulated in *Mfap5* ^*−/−*^ fibroblasts at day 3 post‐wounding compared to *Mfap5* ^*+/+*^ fibroblasts, as were biological processes relating to smooth muscle function. Together, these results suggest that *Mfap5* ^*−/−*^ fibroblasts generate less contractile force during wound healing than wildtype. In addition to changes in contraction, our H&E staining suggests a role for MFAP5 in re‐epithelialization, as *Mfap5* ^−/−^ mice exhibited delayed early re‐epithelialization as compared to *Mfap5* ^*+/+*^ mice. While single‐cell RNA‐sequencing analysis did not find keratinocytes to be a major expresser of MFAP5, our gene enrichment analysis found that *Mfap5* ^*−/−*^ fibroblasts have decreased expression of genes involved in regulating epithelial cell migration. This may contribute to the initial delay in external wound closure, as interactions amongst keratinocytes, dermal fibroblasts, and ECM are vital for wound re‐epithelialization [65]. Interestingly, our previous studies have shown that the application of MFAP5‐neutralizing antibodies does not delay wound closure in vivo [4]. This discrepancy could be due to incomplete neutralization of MFAP5 in our previous work. While early wounds may be larger in *Mfap5* ^*−/−*^ mice as compared to *Mfap5* ^*+/+*^ mice, the wounds re‐epithelialized and closed at the same time. This suggests that the overall physiologic significance of MFAP5 in wound closure may be minimal or limited to the early phase of wound healing. Still, our current findings suggest that MFAP5 may play, albeit a small, role in wound closure through either fibroblast‐mediated wound contraction, keratinocyte functions related to re‐epithelialization, or both.

Wound angiogenesis was decreased in *Mfap5* ^*−/−*^ mice relative to *Mfap5* ^*+/+*^ mice only at day 7 post‐wounding. This agrees with our prior finding that antibody neutralization of MFAP5 could reduce early wound angiogenesis [4]. However, given that by day 14 post‐wounding, CD31 staining was equal between *Mfap5* ^*−/−*^ and *Mfap5* ^*+/+*^ mice, the overall effect that MFAP5 has on wound angiogenesis may be less significant. It may be that stronger modulators of angiogenesis are released during healing and easily compensate for the loss of MFAP5, rendering MFAP5 physiologically dispensable for skin wound healing. At the very least, our results demonstrate that MFAP5 may promote angiogenesis, a function previously described in cancer models and during the early stages of healing [9, 15, 66]. One mechanism by which MFAP5 influences wound angiogenesis is through direct regulation of endothelial cell phenotype, which may occur through MFAP5‐mediated inhibition of notch signaling in endothelial cells [9]. Further analysis of our single‐cell RNA‐sequencing data may help determine if notch signaling is dysregulated in endothelial cells and if fibroblast‐endothelial cell interactions are affected by the loss of MFAP5. Our single‐cell RNA‐sequencing analysis found genes relating to VEGF signaling to be downregulated in *Mfap5* ^*−/−*^ fibroblasts vs. *Mfap5* ^*+/+*^ fibroblasts in vivo, signifying MFAP5 may also promote angiogenesis through fibroblast‐mediated VEGF regulation. In cancer, enhanced tumor angiogenesis and micro‐vessel leakiness were attributed to activation of MFAP5 downstream of YAP or MFAP5‐induced expression of LPP [15, 66]. Whether these mechanisms observed in human cancers are seen in healing skin wounds is unknown and requires additional studies. MFAP5's contribution to wound angiogenesis also suggests another avenue by which MFAP5 may promote scarring, as studies have established an association between excessive angiogenesis during skin wound repair and scar formation and fibrosis [34, 67, 68, 69, 70, 71, 72, 73, 74].

Surprisingly, in vivo histological and proteomic assays found that *Mfap5* ^*−/−*^ mice only had reduced collagen content in NS but not in wound tissue. This coincided with our in vitro findings, where uninjured *Mfap5* ^*−/−*^ fibroblasts exhibited decreased COL1A1 deposition. *Mfap5* ^*−/−*^ mice also had a thinner dermis in NS with altered collagen composition, as there were more immature collagens than mature collagens. However, whether the dermis is truly thinner requires analysis of skin from other body sites, as in this current study, only dorsal skin was analyzed. TWOMBLI analysis of NS did not find any differences in collagen organization. However, in vitro TWOMBLI analysis found that *Mfap5* ^*−/−*^ fibroblasts had an altered ability to organize COL1A1 and COL3A1 fibers. This discrepancy may be due to the complexity with which collagens are organized in vivo versus in a controlled in vitro environment, where only two collagen types are assessed. Overall, our results still suggest that MFAP5 is important for the synthesis and deposition of collagens by fibroblasts.

Fibroblast Clusters 0 and 2 were the major expressers of MFAP5 in NS and during wound healing. The number of Cluster 2 fibroblasts decreased as healing progressed, while the number of Cluster 0 fibroblasts increased, eventually becoming the dominant population of wound fibroblasts. Inflammatory cytokines released during wound healing, such as TGF‐ß and TNF‐α, induce MFAP5 expression, as can fibroblast engulfment of apoptotic cells [3, 4, 75]. In wounds, then, fibroblast expression of MFAP5 may transform other fibroblast clusters into being more similar to Cluster 0 fibroblasts. This idea might explain the net increase in MFAP5‐expressing fibroblasts during the later proliferative and remodeling phases of wound healing [4]. Cluster 0 fibroblasts also more highly express engrailed homeobox 1 (*En1*), which has been described as a marker for fibroblasts involved in skin scar formation during healing [1, 76]. Gene enrichment analysis for Cluster 0 and 2 fibroblasts further suggests these fibroblasts are involved in ECM deposition and angiogenesis. Cluster 0 fibroblasts likely encompass wound‐activated fibroblasts engaged in these processes, while Cluster 2 fibroblasts encompass fibroblasts that are functionally similar in unwounded NS.

Our in vitro studies, occurring outside the wound environment, provide insight into the direct effects of MFAP5 loss on fibroblast function. Gene expression of many prominent collagens in NS and skin wounds, including *Col1a1, Col1a2*, and *Col3a1*, was downregulated in *Mfap5* ^−/−^ fibroblasts in vitro. ECM synthesis and organization processes were also downregulated in *Mfap5* ^−/−^ fibroblasts, suggesting that MFAP5 is important for regulating these fibroblast functions. In congruence with these findings, *Mfap5* ^−/−^ fibroblasts also exhibited decreased deposition of COL1A1 and altered COL1A1 and COL3A1 organization in vitro. These findings may derive from MFAP5's ability to bind fibroblast αvß_3_ integrin, which promotes fibroblast contraction and collagen expression [5, 14, 58, 77, 78, 79]. In support of this idea, the expression of *Itgb3*, a subunit of αvß_3_ integrin, was significantly downregulated in *Mfap5* ^−/−^ fibroblasts. Another important pathway that mediates MFAP5 activity is likely to be TGFß and Notch signaling, as the expression of this pathway is inhibited in *Mfap5* ^−/−^ fibroblasts vs. *Mfap5* ^+/+^ fibroblasts in vitro [80, 81, 82].

An unexpected in vivo result was the elevated presence of neutrophils and macrophages during the early inflammatory phase of wound healing in *Mfap5* ^*−/−*^ mice compared to *Mfap5* ^*+/+*^ mice. Whether this is due to enhanced influx or local proliferation of infiltrating immune cells remains to be determined. Single‐cell and mRNA sequencing analyses found that *Mfap5* ^−/−^ fibroblasts have increased expression of genes related to immune cell processes. However, whether MFAP5 promotes or inhibits inflammation is unknown. Previous documentation of a role for MFAP5 as a modulator of immune cell function is also limited [83, 84, 85, 86, 87, 88, 89]. Therefore, further studies exploring the exact influence that MFAP5 has on inflammatory cell influx are warranted.

Neonatal fibroblasts were used due to their higher proliferative capacity and lower susceptibility to senescence compared to adult fibroblasts, thereby avoiding common pitfalls associated with using adult fibroblasts in vitro [90, 91, 92, 93, 94]. However, neonatal and adult fibroblasts have differential responses to injury due to underlying transcriptomic differences [90, 91, 92, 93, 94, 95]. Our studies, therefore, may not wholly infer the function of MFAP5 in adult fibroblasts in vitro. Though this limits the scope of our work, the use of neonatal fibroblasts still provides a valuable starting point for understanding the role of MFAP5 on fibroblast behavior. Future studies using adult fibroblasts may be performed to validate our findings or reveal a different role for MFAP5. Additionally, our in vivo and in vitro mouse studies used both male and female mice, as sex hormones have been shown to influence parameters of wound healing [27, 96, 97, 98, 99, 100]. Although our work was not intended to assess sex as a biological variable, we observed potential sex‐based differences in wound healing parameters and fibroblast function, including external wound closure, in vitro fibroblast migration, and expression of ECM genes. Evaluating the impact of sex on wound healing and on the regulation or function of MFAP5 will thus be a topic of future studies.

While MFAP5 may be dispensable for wound resolution, our studies still demonstrate novel roles for MFAP5 in wound healing. Loss of MFAP5 reduced collagen deposition and inhibited fibroblast behaviors related to scarring. These findings support the idea that a primary mechanism by which MFAP5 promotes scar formation in wound healing is via the regulation of fibroblast function. Additional studies examining the effect that MFAP5 has on other skin wound cells, including keratinocytes, endothelial cells, and inflammatory cells, would also be informative. Though we do not directly demonstrate a role for MFAP5 in fibrosis, studies have shown that MFAP5 expression is also in fibroblasts of fibrotic conditions such as idiopathic pulmonary fibrosis and systemic sclerosis‐associated skin and lung fibrosis [21, 22, 23, 24, 25]. Our prior work has also shown that MFAP5 is heavily expressed in the dermis and epidermis of keloids [4], which coincides with single‐cell RNA‐sequencing and partial transcriptomics studies demonstrating that keloid fibroblasts significantly overexpress MFAP5 as compared to NS fibroblasts [101, 102]. Collectively, these studies indicate that MFAP5 could play a general role in promoting scar formation and possibly fibrosis. Overall, our current work provides a novel genomic dataset that can be used to further understand wound healing and demonstrates that MFAP5 is a multifunctional glycoprotein in wound healing.

## Author Contributions

C.H., L.C., T.J.K., R.P.M., and L.A.D.: conceptualization. C.H., H.Y., and L.C.: data curation and investigation. L.C., and L.A.D.: contributed reagents/materials/analysis tools. C.H., H.Y., L.C., T.J.K., R.P.M., and L.A.D.: writing, review, and editing. All authors have read and agreed to the published version of the manuscript.

## Funding

This work was partially supported by NIH grants: R35 GM139603 (L.A.D.), F31 AR083830 (H.Y.), and F31 AR082287 (C.H.).

## Conflicts of Interest

The authors declare no conflicts of interest.

## Supporting information


**Figure S1:** fsb271273‐sup‐0001‐Figures.pdf.

**Table S1:** fsb271273‐sup‐0002‐Tables.pdf.

## Data Availability

All mRNA‐sequencing and single‐cell RNA‐sequencing‐related data are available in NCBI GEO with the accession numbers GSE297070 and GSE297449, respectively. The results of the analyses performed on our mRNA‐sequencing and single‐cell RNA‐sequencing data stated in this manuscript may be found in our Figures S1–S4 and Tables S1–S26 and in our NCBI GEO submissions referenced above. Primary datasets associated with each figure have been uploaded and may be found using the following link: https://doi.org/10.25417/uic.30717386. The R code used for our mRNA‐sequencing and single‐cell RNA‐sequencing analysis in this manuscript is available on GitHub with the following links: https://github.com/ChenHanMDPhD/mRNA‐Sequencing‐for‐MFAP5‐Paper‐2/blob/main/Bulk%20mRNA‐sequencing%20Code%20for%20Manuscript.R and https://github.com/ChenHanMDPhD/single‐cell‐RNA‐sequencing‐analysis‐for‐MFAP5‐KO‐mice/blob/main/Single‐cell%20RNA‐sequencing%20code%20for%20manuscript.R.
