# Supplementary material for: Loss of MFAP5 and Its Effect on Skin Homeostasis and Wound Healing
Source: FASEB J. 2025 Dec 5;39(23):e71273. doi: 10.1096/fj.202501770R (PMC12680048; doi:10.1096/fj.202501770R)
Supplement: Supplementary file 1 — Figure S1: fsb271273‐sup‐0001‐Figures.pdf. [file FSB2-39-e71273-s001.pdf]

Supplementary Figure 1:

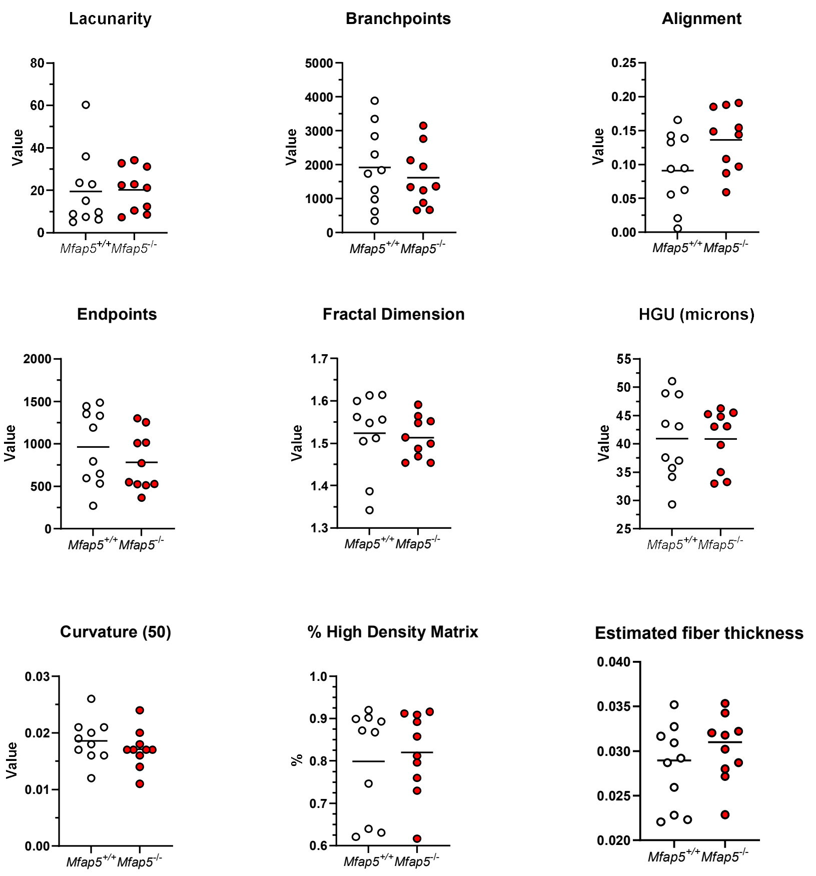

Supplementary Figure 1: TWOMBLI analysis of non-polarized images of picrosirius-stained *Mfap5*$^{+/+}$ and *Mfap5*$^{-/-}$ mice NS suggests the loss of MFAP5 does not affect collagen organization *in vivo*. *Mfap5*$^{+/+}$ and *Mfap5*$^{-/-}$ mice NS underwent picrosirius red staining, imaged with a non-polarized lens, and underwent TWOMBLI analysis to assess differences between the organization of *Mfap5*$^{+/+}$ and *Mfap5*$^{-/-}$ mice ECM in NS.

Supplementary Figure 2:

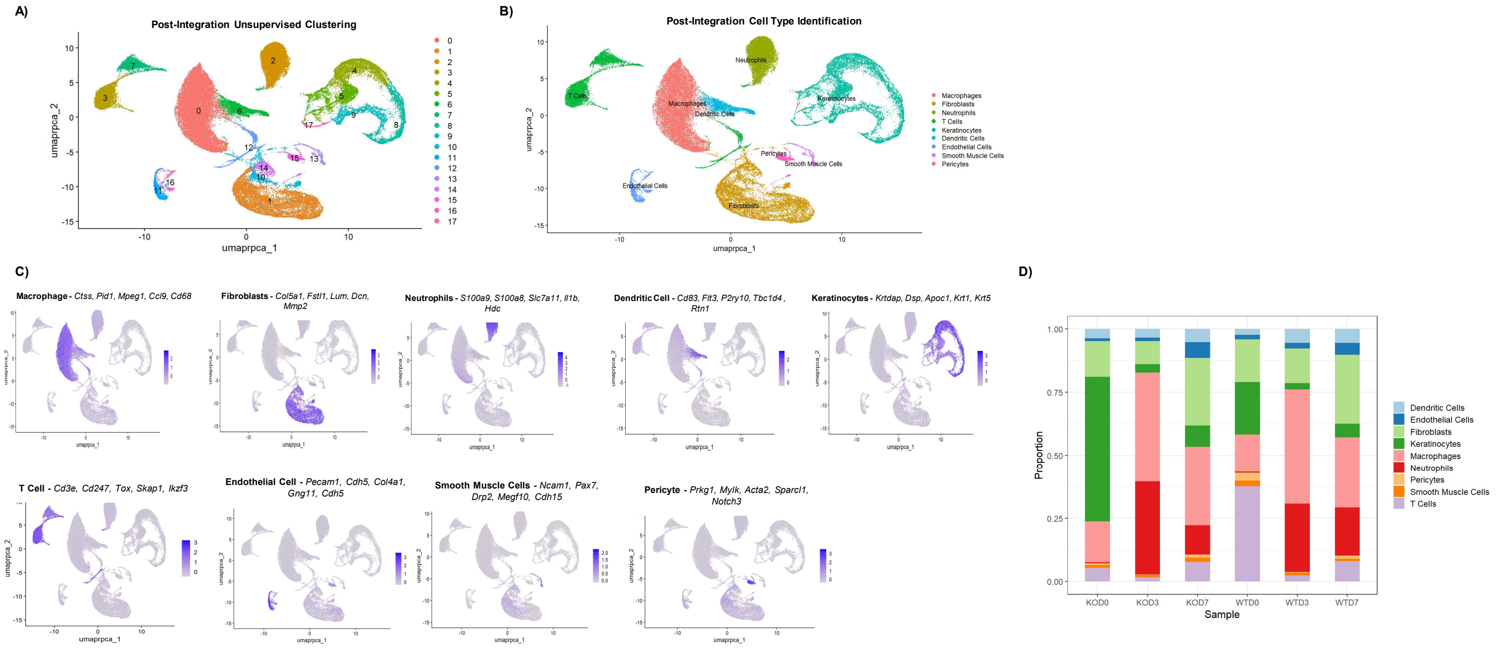

Supplementary Figure 2: Integrated analysis of single-cell RNA-sequencing datasets from mouse unwounded normal skin and wound tissue identifies unique cell populations across a biological timescale of healing. Single-cell RNA-sequencing was performed on *Mfap5*$^{+/+}$ and *Mfap5*$^{-/-}$ mouse normal skin (NS) and on tissue from days 3- and 7-post-wounding to generate tissue specific datasets that were then integrated to form a singular dataset. UMAP plot of the integrated single-cell RNA-sequencing dataset with unsupervised Seurat clustering (A) and with cell type identification (B) based on differential marker gene expression. Seurat clusters and cell type are color coded with annotations shown on the right. C) Feature plot of marker genes used to distinguish each cell type. D) Relative cell type composition arranged according to study timeline.

Supplementary Figure 3:

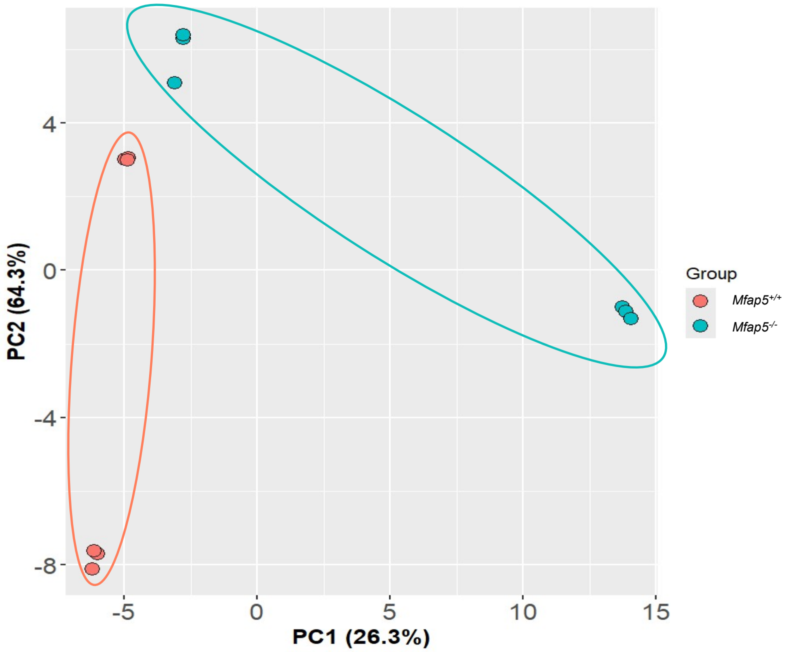

Supplementary Figure 3: Principal component analysis of mouse fibroblasts distinguishes the samples by genotype. Principal component analysis of mRNA-sequencing expression data was performed and plotted for *Mfap5*$^{+/+}$ and *Mfap5*$^{-/-}$ mouse fibroblasts. Each sample is represented by a colored point on the graph. Ellipses are drawn around common groups. The x-axis and y-axis are the first and second principal components, respectively.

Supplementary Figure 4:

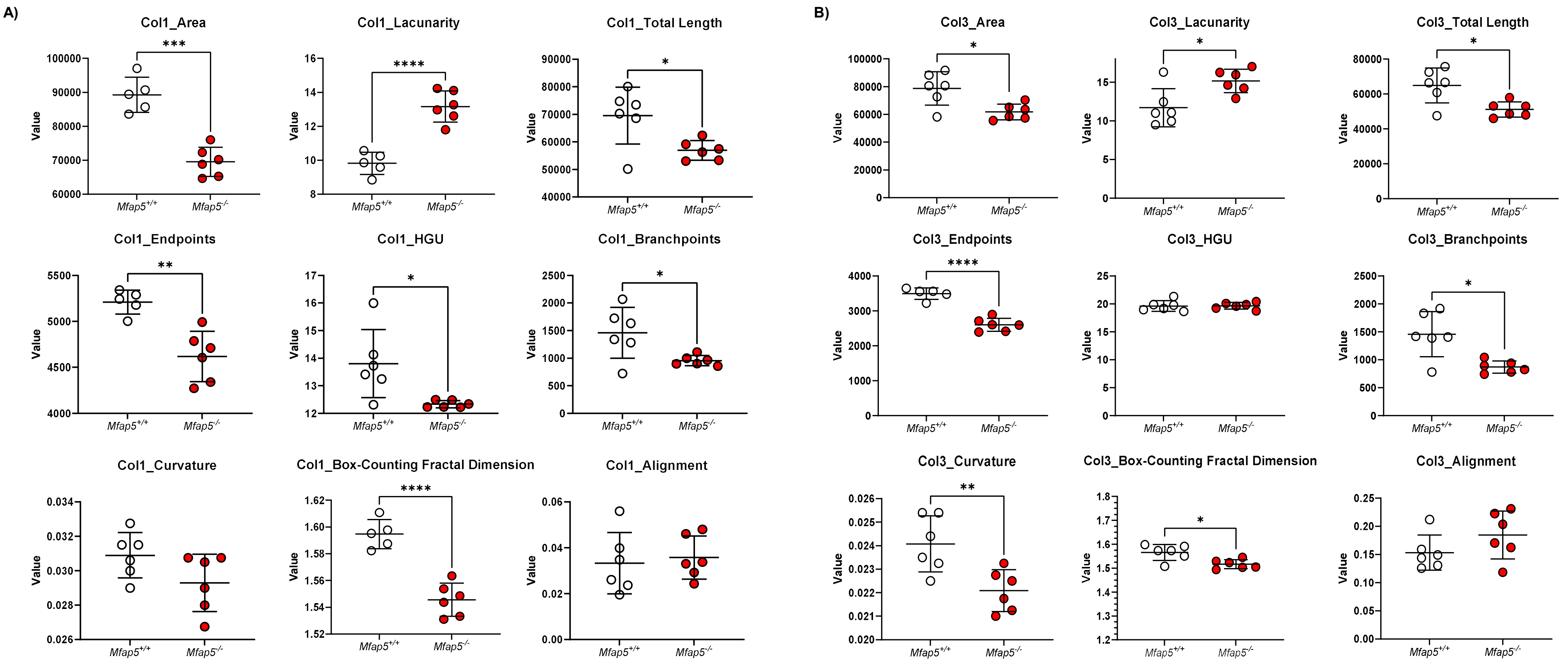

Supplementary Figure 4: TWOMBLI analysis of COL1A1 and COL3A1 in *Mfap5*$^{+/+}$ and *Mfap5*$^{-/-}$ fibroblast deposited ECM *in vitro* suggests the loss of MFAP5 affects collagen organization. Confluent *Mfap5*$^{+/+}$ and *Mfap5*$^{-/-}$ fibroblasts were treated with 100 μg/mL ascorbic acid for 7 days and then fixed with 4% paraformaldehyde. Immunofluorescent staining for COL1A1 and COL3A1 was performed, followed by TWOMBLI analysis to assess differences between the organization of *Mfap5*$^{+/+}$ and *Mfap5*$^{-/-}$ fibroblast generated COL1A1 and COL3A1. TWOMBLI analysis output for COL1A1 (A) and COL3A1 (B) for *Mfap5*$^{+/+}$ and *Mfap5*$^{-/-}$ fibroblasts. * = $p < 0.05$, ** = $p < 0.01$, *** = $p < 0.001$, and **** = $p < 0.0001$.
