# Supplementary material for: Loss of MFAP5 and Its Effect on Skin Homeostasis and Wound Healing
Source: FASEB J. 2025 Dec 5;39(23):e71273. doi: 10.1096/fj.202501770R (PMC12680048; doi:10.1096/fj.202501770R)
Supplement: Supplementary file 2 — Table S1: fsb271273‐sup‐0002‐Tables.pdf. [file FSB2-39-e71273-s002.pdf]

Supplementary Table 1: Cell metrics

	KOFD0	KOFD3	KOFD7	KOMD0	KOMD3	KOMD7	WTFD0	WTFD3	WTFD7	WTMD0	WTMD3	WTMD7
Estimated Number of Cells	6,707	8,348	5,895	8,935	6,960	7,146	9,720	5,947	8,527	10,869	7,784	7,103
Fraction Reads in Cells	93.80%	92.50%	85.30%	95.70%	91.00%	91.80%	90.00%	92.50%	92.90%	94.00%	91.40%	88.00%
Mean Reads per Cell	72,166	87,222	120,547	37,594	61,121	73,778	48,607	130,770	83,673	40,780	50,105	56,087
Total Genes Detected	24,131	25,230	21,815	24,496	24,587	25,283	24,345	24,903	23,520	24,915	24,536	24,873
Median Genes per Cell	3,561	2,551	834	3,795	2,288	2,812	2,438	2,851	1,426	2,726	1,830	1,463
Median UMI Counts per Cell	13,183	7,550	1,365	13,319	6,475	9,028	6,380	9,257	2,795	7,532	4,604	3,837

Supplementary Table 2: Sequencing metrics

	KOFD0	KOFD3	KOFD7	KOMD0	KOMD3	KOMD7	WTFD0	WTFD3	WTFD7	WTMD0	WTMD3	WTMD7
Number of Reads	484,020,157	728,125,591	710,626,218	335,902,632	425,405,084	527,218,925	472,461,381	777,689,705	713,476,738	443,240,975	390,017,489	398,386,383
Base of Reads(G)	145	218	213	100	127	158	141	233	214	132	117	119
Valid Barcodes	96.20%	94.90%	95.70%	96.20%	95.40%	95.20%	94.90%	94.70%	96.20%	95.70%	95.30%	94.70%
Sequencing Saturation	72.20%	76.40%	97.40%	48.10%	71.30%	69.30%	72.90%	80.00%	93.80%	63.70%	70.50%	70.80%
Q30 Bases in Barcode	96.40%	96.70%	96.80%	96.20%	95.50%	95.90%	95.80%	96.80%	96.80%	95.90%	95.20%	95.40%
Q30 Bases in RNA Read	95.10%	95.60%	96.00%	95.40%	95.50%	95.20%	95.50%	95.80%	95.70%	95.90%	94.30%	95.20%
Q30 Bases in UMI	96.80%	97.50%	97.50%	96.30%	96.30%	96.10%	96.00%	97.50%	97.50%	96.10%	95.50%	96.00%

Supplementary Table 3: Mapping metrics

	KOFD0	KOFD3	KOFD7	KOMD0	KOMD3	KOMD7	WTFD0	WTFD3	WTFD7	WTMD0	WTMD3	WTMD7
Reads Mapped to Genome	95.20%	95.40%	95.70%	95.70%	95.00%	95.20%	93.60%	95.30%	95.90%	95.40%	94.70%	93.00%
Reads Mapped Confidently to Genome	93.30%	93.60%	93.80%	94.10%	93.10%	93.30%	91.50%	93.40%	94.10%	93.40%	92.90%	91.20%
Reads Mapped Confidently to Intergenic Regions	3.60%	4.70%	3.40%	3.20%	5.20%	4.90%	4.80%	5.00%	3.20%	4.50%	5.20%	5.70%
Reads Mapped Confidently to Intronic Regions	20.70%	23.80%	17.50%	26.20%	21.10%	23.40%	22.60%	23.60%	18.60%	24.10%	22.80%	22.90%
Reads Mapped Confidently to Exonic Regions	68.90%	65.10%	72.90%	64.80%	66.80%	65.10%	64.10%	64.80%	72.20%	64.90%	64.90%	62.60%
Reads Mapped Confidently to Transcriptome	83.90%	84.10%	84.00%	84.60%	82.20%	82.80%	79.10%	84.00%	84.90%	81.80%	81.90%	79.00%
Reads Mapped Antisense to Gene	5.40%	4.50%	6.00%	6.00%	5.30%	5.40%	7.40%	4.10%	5.60%	6.80%	5.50%	6.20%

Supplementary Table 4: Proportion of each cell type relative to total number of cells at each time point for *Mfap5*$^{-/-}$ and *Mfap5*$^{+/+}$ mice

	Macrophages	Fibroblasts	Neutrophils	T Cells	Keratinocytes	Dendritic Cells	Endothelial Cells	Smooth Muscle Cells	Pericytes
WTD0	14.70	16.80	0.50	37.73	20.69	2.44	1.79	2.25	3.10
KOD0	16.18	14.11	0.40	5.38	57.20	3.71	1.14	1.16	0.72
WTD3	45.23	13.66	27.09	2.38	2.51	5.52	2.21	1.13	0.27
KOD3	43.04	9.14	36.78	1.72	3.30	3.56	1.33	0.96	0.17
WTD7	27.91	27.11	19.02	8.09	5.40	5.59	4.68	0.94	1.27
KOD7	31.10	26.67	11.67	7.85	8.43	5.28	6.23	1.63	1.14

Supplementary Table 5: Proportion of each fibroblast subcluster relative to the total number of fibroblasts at each time point for *Mfap5* $^{-/-}$ and *Mfap5* $^{+/+}$ mice

	0	1	2	3	4	5
WTD0	2.89	63.81	19.59	1.35	10.17	2.18
KOD0	2.31	56.75	33.94	2.42	3.64	0.94
WTD3	74.50	1.72	0.79	21.77	1.08	0.14
KOD3	78.51	1.20	0.00	18.67	1.41	0.20
WTD7	89.81	2.61	0.09	7.32	0.17	0.00
KOD7	84.73	4.18	0.73	10.09	0.26	0.00

Supplementary Table 6: Significant biological processes gene ontology terms for marker genes in fibroblast subcluster 0

Term	Overlap	P.value	Adjusted.P.value	Odds.Ratio	Combined.Score	Genes
Extracellular Matrix Organization (GO:0030198)	32/176	1.96355E-16	5.1543E-13	7.53364632	272.466435	COL16A1;COL12A1;LOXL3;PLOD3;NID2;LOXL2;ADAMTS16;ADAMTS15;ADAMTS14;QSOX1;COLGALT1;ADAMTS7;POSTN;CRTAP;COL27A1;COL24A1;APLP1;P3H4;MMP9;COL1A1;MMP11;MMP14;COL3A1;BMP1;COL1A2;LOX;COL5A1;COL5A3;COL7A1;COL5A2;PXDN;MMP19
Collagen Fibril Organization (GO:0030199)	17/42	1.3097E-15	1.719E-12	22.5986278	774.43185	CRTAP;COL27A1;COL12A1;LOXL3;PLOD3;P3H4;LOXL2;COL1A1;COL3A1;BMP1;COL1A2;COL5A1;LOX;COL5A3;COL5A2;PXDN;COLGALT1
Extracellular Structure Organization (GO:0043062)	20/109	7.59475E-11	5.9236E-08	7.48198373	174.337549	POSTN;COL16A1;COL24A1;APLP1;MMP9;ADAMTS16;COL1A1;ADAMTS15;MMP11;ADAMTS14;MMP14;COL3A1;COL1A2;COL5A1;COL5A3;COL7A1;COL5A2;PXDN;MMP19;ADAMTS7
External Encapsulating Structure Organization (GO:0045229)	20/110	9.02644E-11	5.9236E-08	7.39846743	171.11381	POSTN;COL16A1;COL24A1;APLP1;MMP9;ADAMTS16;COL1A1;ADAMTS15;MMP11;ADAMTS14;MMP14;COL3A1;COL1A2;COL5A1;COL5A3;COL7A1;COL5A2;PXDN;MMP19;ADAMTS7
Supramolecular Fiber Organization (GO:0097435)	31/316	7.95596E-09	4.1769E-06	3.65408689	68.1463245	COL12A1;LOXL3;PLOD3;LTBP2;LOXL2;RND1;CNN2;ADAMTS14;EFEMP2;RAC3;EMILIN1;COLGALT1;CRTAP;MARCKSL1;COL27A1;RIPK3;TPM4;ACTN1;P3H4;MYO7A;COL1A1;COL3A1;MYO1B;COL1A2;BMP1;LOX;COL5A1;COL5A3;PXDN;COL5A2;MYL9
Positive Regulation Of Angiogenesis (GO:0045766)	17/119	9.82318E-08	4.2976E-05	5.51686678	89.0198078	PTGIS;SERPINE1;VASH2;HMGA2;SIRT6;BRCA1;HIF1A;PGF;AQP1;RUNX1;HIPK2;SFRP2;PKM;TNN;MDK;EMILIN1;ANGPTL4
Spindle Assembly Checkpoint Signaling (GO:0071173)	8/26	6.06681E-07	0.00017695	14.5510511	208.302126	CENPF;ZWILCH;NUF2;PLK1;TTK;KNTC1;TRIP13;BUB1
Mitotic Spindle Assembly Checkpoint Signaling (GO:0007094)	8/26	6.06681E-07	0.00017695	14.5510511	208.302126	CENPF;ZWILCH;NUF2;PLK1;TTK;KNTC1;TRIP13;BUB1
Mitotic Spindle Checkpoint Signaling (GO:0071174)	8/26	6.06681E-07	0.00017695	14.5510511	208.302126	CENPF;ZWILCH;NUF2;PLK1;TTK;KNTC1;TRIP13;BUB1
Negative Regulation Of Mitotic Metaphase/Anaphase Transition (GO:0045841)	8/28	1.14463E-06	0.00030047	13.0945946	179.139638	CENPF;ZWILCH;NUF2;PLK1;TTK;KNTC1;TRIP13;BUB1

Positive Regulation Of Cell Differentiation (GO:0045597)	24/283	5.18385E-06	0.0011488	3.07931145	37.4751033	FBN2;MMD;HGF;SOX11;SIRT6;PTN;TMEM64;ACTB;RUNX2;LOXL2;COL1A1;SFRP2;GDNF;MDK;GPC1;NOCT;SNAI1;TMEM119;CCN4;ECT2;BMPR1B;BRINP3;SOX4;BEND6
Skin Development (GO:0043588)	11/68	5.25167E-06	0.0011488	6.33762249	77.0462491	COL1A1;UGCG;COL3A1;COL1A2;COL5A1;CASP3;COL5A3;COL5A2;PDGFA;WNT16;EREG
Protein Localization To Kinetochore (GO:0034501)	5/11	9.50304E-06	0.00191888	27.162465	314.103997	ZWILCH;CDK1;KNTC1;TTK;SPDL1
Positive Regulation Of Vasculature Development (GO:1904018)	13/104	1.4055E-05	0.00263531	4.69919688	52.5019407	PTGIS;SERPINE1;VASH2;HMGA2;BRCA1;HIF1A;PGF;AQP1;RUNX1;HIPK2;SFRP2;EMILIN1;ANGPTL4
Generation Of Neurons (GO:0048699)	17/172	1.73741E-05	0.00292668	3.62048359	39.6824145	FZD1;FZD3;PCSK9;SOX11;WNT16;RUNX2;RUNX1;CIT;TUBB2B;SIX4;CASP3;DDIT4;TRIM46;MDGA1;MARK1;LHX8;ADGRL3
Positive Regulation Of Multicellular Organismal Process (GO:0051240)	28/387	1.78388E-05	0.00292668	2.59631455	28.3884502	EPAS1;SERPINE1;HILPDA;ENO1;PTN;TSKU;ACTB;LOXL2;C1QTNF3;SIX4;MDK;SPP1;TNFSF11;F2R;SOX11;SIRT6;TMEM64;INHBA;EREG;COL1A1;BMP1;FABP5;GDNF;SNAI1;LCN2;TMEM119;F2RL1;BMPR1B
Nervous System Development (GO:0007399)	30/433	2.06832E-05	0.00319373	2.48099778	26.7605101	CNTFR;GSTP1;PCSK9;MSI1;PTN;KALRN;CDH2;MDK;ADGRA2;ECT2;BRINP3;SOX4;GDF11;MARCKSL1;STIL;EIF2B2;MAMDC2;APLP1;SOX11;NRG1;INHBA;ECRG4;GDNF;TYRO3;HOXB2;DCHS1;PAFAH1B3;MDGA1;SDK2;ADGRL3
Endodermal Cell Differentiation (GO:0035987)	7/31	2.96543E-05	0.00432459	9.53007307	99.3596085	MMP14;COL7A1;COL12A1;COL6A1;HMGA2;INHBA;MMP9
Regulation Of Angiogenesis (GO:0045765)	18/205	4.88473E-05	0.00674864	3.17762831	31.5437192	PTGIS;CEMIP2;SERPINE1;VASH2;ECSCR;HMGA2;NRG1;BRCA1;THBS2;HIF1A;PGF;AQP1;RUNX1;HIPK2;SFRP2;ADGRA2;EMILIN1;ANGPTL4
Endoderm Formation (GO:0001706)	7/35	6.83384E-05	0.00896941	8.16694772	78.3295152	MMP14;COL7A1;COL12A1;COL6A1;HMGA2;INHBA;MMP9
Protein Localization To Condensed Chromosome (GO:1903083)	4/10	0.000145793	0.01689127	21.6935123	191.625819	ZWILCH;CDK1;TTK;SPDL1
Positive Regulation Of Extracellular Matrix Organization (GO:1903055)	5/18	0.000148	0.01689127	12.5319974	110.510913	EFEMP2;FSCN1;EMILIN1;COLGALT1;RUNX1
Protein Localization To Chromosome, Centromeric Region (GO:0071459)	5/18	0.000148	0.01689127	12.5319974	110.510913	ZWILCH;CDK1;TTK;SPDL1;CENPA
Regulation Of Osteoblast Differentiation (GO:0045667)	10/85	0.000226929	0.02455305	4.36723164	36.6448919	FBN2;TNN;HGF;NOCT;TMEM119;CCN4;SOX11;TMEM64;BMPR1B;RUNX2

Neuron Differentiation (GO:0030182)	15/173	0.000233839	0.02455305	3.12268744	26.108414	FZD1;FZD3;PCSK9;SOX11;MYO7A;INHBA;WNT16;RUNX2;RUNX1;UGCG;CASP3;DDIT4;BRINP3;SOX4;LHX8
Positive Regulation Of Osteoblast Differentiation (GO:0045669)	7/44	0.000308342	0.02965041	6.17752154	49.9409429	FBN2;HGF;TMEM119;CCN4;SOX11;BMPR1B;RUNX2
Glycosaminoglycan Biosynthetic Process (GO:0006024)	8/58	0.00032	0.02965041	5.22972973	42.0846329	CSGALNACT1;CEMIP;GALNT5;CHST11;CHSY1;B3GNT3;PXYLP1;CHST2
Cell-Cell Junction Maintenance (GO:0045217)	4/12	0.000327566	0.02965041	16.2684564	130.535161	F2R;MYADM;F2RL1;INAVA
Spinal Cord Development (GO:0021510)	4/12	0.000327566	0.02965041	16.2684564	130.535161	LOXL3;SOX11;SOX12;SOX4
Epithelial To Mesenchymal Transition (GO:0001837)	7/47	0.000468259	0.04097263	5.71332209	43.8011255	HGF;LOXL3;SNAI1;HMGA2;HIF1A;TBX3;LOXL2
Regulation Of Collagen Fibril Organization (GO:1904026)	3/6	0.000502125	0.04251869	32.4907873	246.82148	EFEMP2;EMILIN1;COLGALT1

Supplementary Table 7: Significant reactome pathway terms for marker genes in fibroblast subcluster 0

Term	Overlap	P.value	Adjusted.P.value	Odds.Ratio	Combined.Score	Genes
Extracellular Matrix Organization R-HSA-1474244	51/291	9.76183E-25	1.0045E-21	7.4162113	410.0137488	PTPRS;COL16A1;COL12A1;SERPINE1;LOXL3;PLOD3;LOXL2;EFEMP2;CAPN6;TNN;CASP3;TIMP1;COLGALT1;COL27A1;ACTN1;BGN;P3H1;MMP9;ASPN;MMP11;MMP14;LOX;COL6A2;PXDN;ADAM12;COL6A1;MMP19;COL6A3;CD44;FBN2;PDGFA;FBLN2;NID2;ADAMTS16;ADAMTS14;FZR1;NCAM1;CRTAP;COL24A1;COL1A1;COL3A1;COL1A2;BMP1;COL5A1;P4HA2;COL5A3;P4HA3;COL7A1;COL5A2;SDC1;P4HB
Collagen Formation R-HSA-1474290	28/90	3.34027E-21	1.7186E-18	15.26799	719.8587692	COL16A1;COL12A1;LOXL3;PLOD3;LOXL2;ADAMTS14;COLGALT1;CRTAP;COL27A1;COL24A1;P3H1;MMP9;COL1A1;COL3A1;BMP1;COL1A2;LOX;COL5A1;P4HA2;COL6A2;COL5A3;COL7A1;P4HA3;COL6A1;PXDN;COL5A2;COL6A3;P4HB
Collagen Biosynthesis And Modifying Enzymes R-HSA-1650814	23/67	9.59326E-19	3.2905E-16	17.535371	727.5084467	CRTAP;COL16A1;COL27A1;COL24A1;COL12A1;PLOD3;P3H1;COL1A1;ADAMTS14;COL3A1;BMP1;COL1A2;COL5A1;P4HA2;COL6A2;COL5A3;COL7A1;P4HA3;COL6A1;COL5A2;COL6A3;P4HB;COLGALT1
Assembly Of Collagen Fibrils And Other Multimeric Structures R-HSA-2022090	18/57	3.14532E-14	8.0913E-12	15.353688	477.3503921	COL27A1;COL24A1;LOXL3;MMP9;LOXL2;COL1A1;COL3A1;BMP1;COL1A2;LOX;COL5A1;COL6A2;COL5A3;COL7A1;COL6A1;PXDN;COL5A2;COL6A3
Collagen Chain Trimerization R-HSA-8948216	14/44	2.06365E-11	4.247E-09	15.425484	379.5280076	COL16A1;COL27A1;COL24A1;COL12A1;COL1A1;COL3A1;COL1A2;COL5A1;COL5A3;COL6A2;COL7A1;COL5A2;COL6A1;COL6A3
Resolution Of Sister Chromatid Cohesion R-HSA-2500257	14/106	3.43142E-06	0.00058849	5.0139487	63.08818696	ZWILCH;PLK1;CENPA;CCNB2;CENPF;CENPI;NUF2;CDK1;PMEPA1;CENPM;KNTC1;CENPN;SPDL1;BUB1
Crosslinking Of Collagen Fibrils R-HSA-2243919	5/10	5.31478E-06	0.00078127	32.596639	395.8867778	BMP1;LOX;PXDN;LOXL3;LOXL2
RHO GTPases Activate Formins R-HSA-5663220	14/119	1.34311E-05	0.00172758	4.3902162	49.24915577	ZWILCH;PLK1;RHOD;CENPA;FMNL3;CENPF;DIAPH3;CENPI;NUF2;CENPM;KNTC1;CENPN;SPDL1;BUB1
Post-translational Protein Phosphorylation R-HSA-8957275	13/106	1.73106E-05	0.00197918	4.5976626	50.40965006	PRKCSH;FUCA2;TMEM132A;PCSK9;CKAP4;RCN1;LGALS1;CDH2;MXRA8;QSOX1;TIMP1;P4HB;MFGE8
Degradation Of Extracellular Matrix R-HSA-1474228	13/109	2.34329E-05	0.00241124	4.4532936	47.47821781	COL16A1;COL12A1;MMP9;ADAMTS16;MMP11;MMP14;FZR1;BMP1;CAPN6;CASP3;MMP19;TIMP1;CD44
Regulation Of IGF Transport And Uptake By IGFBPs R-HSA-381426	13/123	8.40037E-05	0.00785817	3.8836921	36.44708802	PRKCSH;FUCA2;TMEM132A;PCSK9;CKAP4;RCN1;LGALS1;CDH2;MXRA8;QSOX1;TIMP1;P4HB;MFGE8

NCAM1 Interactions R-HSA-419037	7/37	9.93299E-05	0.00840743	7.6216976	70.2496762	CACNB3;GDNF;COL6A2;COL6A1;COL6A3;NCAM1;CACNA1C
Unattached Kinetochores Signal Amplification Via A MAD2 Inhibitory Signal R-HSA-141444	11/93	0.000106216	0.00840743	4.3997267	40.25764561	CENPF;ZWILCH;CENPI;NUF2;PLK1;CENPM;KNTC1;CENPN;SPDL1;CENPA;BUB1
Signaling By Non-Receptor Tyrosine Kinases R-HSA-9006927	8/52	0.00014707	0.01067346	5.9447174	52.45977233	DOK1;CCNE1;EPAS1;PXDN;NRG1;UBA52;HIF1A;EREG
EML4 And NUDC In Mitotic Spindle Formation R-HSA-9648025	11/97	0.00015559	0.01067346	4.1942196	36.77612308	CENPF;ZWILCH;CENPI;PLK1;NUF2;CENPM;KNTC1;CENPN;SPDL1;CENPA;BUB1
Signal Transduction R-HSA-162582	102/2465	0.000429885	0.02764699	1.4767271	11.44757668	PRAG1;ZWILCH;SERPINE1;ARHGDIG;PRDM1;GPR176;EDNRA;RGS3;PDK3;KNTC1;SOX4;IER3;TPM4;ACTN1;F2R;PGF;RUNX1;EREG;DOK1;SFRP2;CCNE1;SPATA13;ADAM12;UBA52;MYL9;BEX3;CRABP2;MAGED1;EPAS1;RRAD;CRABP1;PDGFA;HIF1A;KALRN;ECT2;FZD1;PLK1;TNFRSF10B;INHBA;GNG11;ANLN;COL1A2;DIAPH3;GDNF;SNAI1;CDK1;BMPR1B;GABRB3;ARHGAP11A;CXCL9;PTPRS;PRKCSH;HTR2A;CXCL2;CXCL5;RND1;MECOM;CASP3;NUF2;PMEPA1;BDKRB1;SPDL1;GPR39;PLEKHG4;SIRT6;NRG1;WNT16;RHOD;MMP9;CKAP4;ARL4C;ACTA2;MRAS;COL6A2;PXDN;COL6A1;COL6A3;PDE5A;SGK1;USP13;RGS16;PTN;THBS2;CENPA;LMAN1;IL1RL1;PROKR2;FZR1;S1PR1;NCAM1;S1PR3;BUB1;POLR2L;APLN;FMNL3;CENPF;CENPI;TACC3;CENPM;F2RL1;CENPN;P4HB
Mitotic Spindle Checkpoint R-HSA-69618	11/110	0.000470069	0.028453	3.6410111	27.89972532	CENPF;ZWILCH;CENPI;PLK1;NUF2;CENPM;KNTC1;CENPN;SPDL1;CENPA;BUB1
Phosphorylation Of Emi1 R-HSA-176417	3/6	0.000502125	0.02870484	32.490787	246.8214804	FZR1;PLK1;CDK1
Cell Cycle Checkpoints R-HSA-69620	19/271	0.00057024	0.03088297	2.484851	18.56048006	RRAD;ZWILCH;PLK1;BRCA1;CENPA;CCNB2;CENPF;DBF4;CCNE2;CENPI;CCNE1;NUF2;CDK1;CENPM;CENPN;KNTC1;SPDL1;UBA52;BUB1
Mitotic Anaphase R-HSA-68882	17/232	0.000668126	0.03435778	2.6019785	19.02315295	NDC1;ZWILCH;PLK1;CENPA;CCNB2;TUBB2B;CENPF;CENPI;NUF2;CDK1;CENPM;PMEPA1;CENPN;KNTC1;SPDL1;UBA52;BUB1
Mitotic Metaphase And Anaphase R-HSA-2555396	17/233	0.000701179	0.03435778	2.5897973	18.80904298	NDC1;ZWILCH;PLK1;CENPA;CCNB2;TUBB2B;CENPF;CENPI;NUF2;CDK1;CENPM;PMEPA1;CENPN;KNTC1;SPDL1;UBA52;BUB1
Activation Of NIMA Kinases NEK9, NEK6, NEK7 R-HSA-2980767	3/7	0.000859136	0.03875735	24.366834	172.0197062	CCNB2;PLK1;CDK1
O-glycosylation Of TSR Domain-Containing Proteins R-HSA-5173214	6/38	0.000866297	0.03875735	6.1136364	43.10898172	ADAMTS16;ADAMTS15;ADAMTS14;POFUT2;THBS2;ADAMTS7

Glycosaminoglycan Metabolism R-HSA-1630316	11/120	0.000978602	0.0410855	3.30526	22.90341965	CSGALNACT1;CEMIP;CHST11;CHSY1;GPC1;BGN;B3GNT3;SDC1;HS6ST2;CD44;CHST2
Elastic Fibre Formation R-HSA-1566948	6/39	0.00099819	0.0410855	5.9280686	40.96038639	FBN2;EFEMP2;LOX;LOXL3;FBLN2;LOXL2
Metabolism Of Carbohydrates R-HSA-71387	19/285	0.001042422	0.04125585	2.3523482	16.1517133	NDC1;CSGALNACT1;CEMIP;BGN;ENO1;HS6ST2;GALE;PFKL;G6PC3;PKM;CHST11;CHSY1;GPC1;B3GNT3;SDC1;UBA52;CD44;CHST2;GALK1
Polo-like Kinase Mediated Events R-HSA-156711	4/16	0.001094581	0.04171569	10.8434	73.92362174	CCNB2;CENPF;PLK1;FOXM1
Collagen Degradation R-HSA-1442490	6/40	0.001144978	0.04207793	5.7534165	38.96426668	MMP11;MMP14;COL16A1;COL12A1;MMP19;MMP9
Diseases Of Glycosylation R-HSA-3781865	12/143	0.00127544	0.04525614	3.0018695	20.0058507	ADAMTS16;ADAMTS15;ADAMTS14;GALE;CHSY1;GPC1;BGN;SDC1;MOGS;THBS2;GALK1;ADAMTS7

Supplementary Table 8: Significant biological processes gene ontology terms for marker genes in fibroblast subcluster 2

Term	Overlap	P.value	Adjusted.P.value	Odds.Ratio	Combined.Score	Genes
Regulation Of Cell Migration (GO:0030334)	47/434	2.77362E-08	8.74964E-05	2.654525586	46.19014645	KANK1;CCL11;TNXB;CSF1;SEMA3C;ROCK2;SEMA3D;CITED2;SEMA3E;PTPRJ;LAMC2;ADARB1;FGF2;EGFR;DOCK10;WNT11;PLAU;PDGFD;KDR;HAS2;EMILIN2;FYN;RFFL;SRGAP1;NGFR;NTNG2;EDN1;ACE;ANXA1;IGFBP5;LIMCH1;MAGI2;PRKCA;MITF;TBX5;DUSP22;BST2;TMEFF2;DAB2;BMP2;FER;KIF2A;NAV3;FGF18;DPEP1;EPHA1;RECK
Transmembrane Receptor Protein Tyrosine Kinase Signaling Pathway (GO:0007169)	35/284	7.32733E-08	8.74964E-05	3.052208835	50.14495047	CSF1;ARHGEF28;PXN;PTPRJ;MST1R;EFNA5;SOGA1;FGF2;EGFR;SOCS2;GHR;FLRT2;FLRT3;PDGFD;NAMPT;KDR;PDK4;GRB10;MAPK1;FYN;NTRK2;SYK;IL31RA;VEGFD;SORBS1;NGF;FER;NR4A3;AXL;FGF18;RAPGEF1;TEK;EPHA1;MET;DDR2
Negative Regulation Of Cell Migration (GO:0030336)	25/163	8.23625E-08	8.74964E-05	3.911846753	63.81057476	KANK1;CITED2;NEXMIF;PTPRJ;ADARB1;FGF2;WNT11;HAS1;EMILIN2;SRGAP1;CLASP2;NGFR;IGFBP5;LIMCH1;MAGI2;MITF;MCC;TBX5;BST2;DUSP22;TMEFF2;NAV3;DPEP1;EPHA1;RECK
Negative Regulation Of Cell Motility (GO:2000146)	21/133	5.39299E-07	0.000429687	4.035926871	58.25051165	NGFR;KANK1;IGFBP5;LIMCH1;CITED2;MAGI2;MITF;PTPRJ;ADARB1;TBX5;BST2;DUSP22;TMEFF2;WNT11;NAV3;SPOCK3;DPEP1;EMILIN2;EPHA1;SRGAP1;RECK
Cellular Response To Growth Factor Stimulus (GO:0071363)	22/155	1.86983E-06	0.001062172	3.5606155	46.96331328	WWOX;RAMP2;ANXA1;MAGI2;KLF4;BMP7;BMP6;EGFR;ZFP36L1;BMP4;TMEM100;DUSP22;BMP2;CLEC3B;HAS1;KDR;PDE3A;RAPGEF1;HAS2;PDE8A;WNT2;SOX5
Regulation Of Angiogenesis (GO:0045765)	26/205	1.9997E-06	0.001062172	3.133256467	41.11620674	RAMP2;SP100;NPR1;ROCK2;GATA6;SEMA3E;ISM1;CXCL13;FGF2;KDR;EMILIN2;GLUL;CD34;ANXA3;KRT1;PRKCA;VEGFD;VASH1;KLF4;PML;CXCL10;STIM1;RHOJ;FGF18;TEK;EPHA1
Positive Regulation Of MAPK Cascade (GO:0043410)	32/310	1.22462E-05	0.005575514	2.487044578	28.1292103	NOTCH2;CCL11;ROCK2;PTPRJ;ETFA;MST1R;NOD2;ADRA1B;FGF2;EGFR;GHR;PDGFD;KDR;PLCE1;LGALS9;IGFBP6;PDE8A;NTRK2;EDN1;KSR1;PRKCA;MTURN;BMP4;BMP2;TAOK3;FGF18;ACKR3;TEK;TLR4;JCAD;GSDME;DDR2
Regulation Of Endothelial Cell Migration (GO:0010594)	14/88	3.78067E-05	0.012858395	4.048308151	41.22401596	EDN1;SP100;ANXA3;ROCK2;ITGB3;PRKCA;FGF2;BMP4;RHOJ;ATOH8;KDR;TEK;PLPP3;GLUL
Transforming Growth Factor Beta Production (GO:0071636)	5/10	3.8674E-05	0.012858395	21.26057906	216.0147667	TNXB;GATA6;LGALS9;PTGS2;CD200
Involved In Sprouting Angiogenesis (GO:0090049)	8/30	4.03464E-05	0.012858395	7.750126968	78.41585149	CARD10;ANXA1;RHOJ;KDR;PTGS2;KLF4;FGF2;JCAD
Migration Involved In Sprouting Angiogenesis (GO:0090050)	6/16	4.51726E-05	0.013015254	12.76722408	127.7363365	ANXA1;RHOJ;KDR;PTGS2;FGF2;JCAD
(GO:0030041)	8/31	5.22404E-05	0.013015254	7.412776293	73.08740603	DIAPH1;GSN;COBL;ANG;WASL;ARHGAP6;WASF3;GAS7
Regulation Of MAP Kinase Activity (GO:0043405)	16/114	5.30901E-05	0.013015254	3.497043462	34.4232143	EDN1;KSR1;PTPRJ;IRAK3;MST1R;NOD2;FGF2;BMP7;EGFR;BMP4;GHR;BMP2;UCHL1;PDGFD;FGF18;TLR4
Positive Regulation Of Epithelial Cell Migration (GO:0010634)	14/95	8.96857E-05	0.019298625	3.697093419	34.45394828	EDN1;ANXA3;ROCK2;IQSEC1;ITGB3;PRKCE;PRKCA;FGF2;BMP4;ATOH8;KDR;TEK;PLPP3;CLASP2

Sprouting Angiogenesis (GO:0002040)	10/52	9.422E-05	0.019298625	5.08052045	47.09580292	BMP4;RAMP2;RNF213;KDR;SEMA3E;VEGFD;TEK;FGF2;RECK;E2F7
Regulation Of Epidermal Cell Differentiation (GO:0045604)	6/18	9.68867E-05	0.019298625	10.63823857	98.31826089	BMP4;ERRFI1;SFRP4;ROCK2;MAFF;ZFP36L1
Positive Regulation Of Endothelial Cell Migration (GO:0010595)	13/85	0.000107758	0.020201424	3.859628589	35.26011624	EDN1;ANXA3;ROCK2;ITGB3;PRKCA;FGF2;BMP4;ATOH8;FGF18;KDR;TEK;PLPP3;MET
Regulation Of Focal Adhesion Assembly (GO:0051893)	10/54	0.000131353	0.021326737	4.849078693	43.33924752	DUSP22;LIMCH1;ROCK2;KDR;PTPRJ;FYN;TEK;EFNA5;ARHGAP6;CLASP2
Positive Regulation Of Epithelial Cell Proliferation (GO:0050679)	16/123	0.000133498	0.021326737	3.201382377	28.5608817	NOTCH2;OSR2;CCL11;ITGB3;PRKCA;VEGFD;NOD2;FGF2;BMP6;EGFR;BMP4;BMP2;NR4A3;KDR;ANG;TEK
Positive Regulation Of Cytokine Production (GO:0001819)	30/320	0.000133836	0.021326737	2.228581585	19.8764891	CD274;ROCK2;CD80;NOD2;PTGS2;TANK;C3;IFIH1;MAPK9;PNP;DHX58;LGALS9;GBP5;AFAP1L2;ANXA1;TSLP;SYK;IL15;IL18;NR1H4;ISG15;IL17RA;LACC1;IRF4;IRF7;HPSE;RAB7B;PLA2R1;TLR4;CD200
Regulation Of MAPK Cascade (GO:0043408)	22/204	0.00014404	0.021859743	2.595265121	22.95621375	NTRK2;PHLPP1;SYK;KSR1;ROCK2;STYX;FN1;PTPRJ;ETFA;ADRA1B;FGF2;EGFR;BMP4;BMP2;TAOK3;KDR;PLCE1;MAPK1;IGFBP6;TEK;JCAD;GSDME
Substrate Adhesion-Dependent Cell Spreading (GO:0034446)	8/36	0.000164009	0.023758869	6.087470072	53.05590706	FER;ITGB3;PXN;FN1;ITGB7;RADIL;TEK;EPHA1
Synthase Biosynthetic Process (GO:0051769)	5/13	0.000176294	0.024194246	13.28577394	114.8337122	EDN1;NAMPT;KDR;NOD2;TLR4
Peptidyl-Tyrosine Phosphorylation (GO:0018108)	11/67	0.000189311	0.024194246	4.193045324	35.94329411	FER;EFEMP1;SYK;IL15;KDR;FYN;TEK;EPHA1;EGFR;JAK1;DDR2
Positive Regulation Of Signal Transduction (GO:0009967)	26/266	0.000189789	0.024194246	2.329351957	19.9616144	KANK1;TNXB;LAMA2;CITED2;CD80;ITGB3;NOD2;RBPJ;NID1;C3;NETO1;GRB10;TSPAN5;CRADD;TSLP;LAMB2;PRKCA;SORBS1;BMP6;BMP2;AXL;BMP2K;TRIM16;TEK;LIMS2;JCAD
Positive Regulation Of Protein Phosphorylation (GO:0001934)	33/377	0.000230686	0.028276802	2.067792702	17.31663138	TNXB;CSF1;ROCK2;CD80;ITGB3;PRKAG2;PTPRJ;CREBL2;EFNA5;FGF2;EGFR;C3;GHR;PDGFD;KDR;MAPK1;NTRK2;RBPMS;LIMCH1;IL15;BMP8A;VEGFD;NGF;BMP7;BMP6;BMP4;BMP3;BMP2;DAB2;SLCO3A1;FGF18;TEK;DDR2
Glycosaminoglycan Biosynthetic Process (GO:0006024)	10/58	0.000243315	0.0286308	4.444055618	36.97967621	HS3ST3B1;UGDH;GLCE;HAS1;UST;CHST1;HAS2;ST3GAL6;ST3GAL1;HS3ST1
Prostanoid Biosynthetic Process (GO:0046457)	5/14	0.000264062	0.0286308	11.80895818	97.29784896	EDN1;PLA2G4A;PTGS2;DAGLB;PTGS1
Regulation Of Endothelial Cell Proliferation (GO:0001936)	13/93	0.000271644	0.0286308	3.472205056	28.51033246	CCL11;ITGB3;PRKCA;VEGFD;VASH1;FGF2;BMP4;BMP2;ATOH8;FGF18;KDR;ANG;TEK
Cellular Response To Organic Substance (GO:0071310)	12/82	0.000300156	0.0286308	3.660798461	29.69349562	GHR;SOCS2;NCOA2;EDN1;RAMP2;CLEC3B;PID1;IGFBP5;SYK;TIPARP;IL18;NR1H4
Restricted SMAD Protein Phosphorylation (GO:0010862)	9/49	0.000302183	0.0286308	4.796224832	38.87089155	BMP4;BMP3;BMP2;DAB2;TNXB;RBPMS;BMP8A;BMP7;BMP6
Regulation Of Cell Communication (GO:0010646)	9/49	0.000302183	0.0286308	4.796224832	38.87089155	SOCS2;ANXA1;LURAP1L;GRK5;VEPH1;LTBP1;MGLL;TRDN;CDC42SE2

Positive Regulation Of Angiogenesis (GO:0045766)	15/119	0.000303557	0.0286308	3.084881757	24.98735944	RAMP2;ANXA3;GATA6;PRKCA;VEGFD;KLF4;FGF2;STIM1;FGF18;KDR;EMILIN2;TEK;EPHA1;CD34;JAK1
Positive Regulation Of Interleukin-10 Production (GO:0032733)	7/30	0.000305443	0.0286308	6.478940217	52.43890307	SYK;TSLP;IRF4;ISG15;NOD2;LGALS9;TLR4
Positive Regulation Of Fat Cell Differentiation (GO:0045600)	9/50	0.000353944	0.032229101	4.678998199	37.18106363	BMP2;ZBTB16;SOX13;SIX1;CREBL2;METRNL;PTGS2;BMP7;ZFP36L1
Cellular Response To Oxygen-Containing Compound (GO:1901701)	34/406	0.000422051	0.036840721	1.969418563	15.3031381	CD274;CPNE8;PID1;PRKAA2;ROCK2;CD80;PXN;RORB;NOD2;CXCL13;EGFR;ZFP36L1;MAPK9;WNT11;PDK4;MAPK1;WNT2;TGM2;GBP3;WNT10B;IGFBP5;DAPK1;NR1H4;TNFRSF1B;PEX13;ESR1;CXCL10;FER;NR4A3;AXL;RAPGEF1;PDE3A;DPEP1;TLR4
Regulation Of Protein Phosphorylation (GO:0001932)	25/265	0.000427708	0.036840721	2.237210516	17.35419609	PRKN;PID1;ROCK2;ITGB3;EFNA5;FGF2;EGFR;C3;KDR;PLCE1;CHP1;FYN;NTRK2;LIMCH1;FN1;VEGFD;BMP7;BMP4;BMP2;DAB2;FER;SLCO3A1;FGF18;TEK;PLPP3
Enzyme-Linked Receptor Protein Signaling Pathway (GO:0007167)	15/124	0.00047483	0.039823269	2.942598562	22.5183916	NTRK2;CSF1;SYK;NPR1;IL31RA;MST1R;NGF;EGFR;AXL;KDR;RAPGEF1;FYN;TEK;MET;DDR2
Negative Regulation Of Wnt Signaling Pathway (GO:0030178)	16/140	0.00059102	0.047407362	2.760010183	20.51697839	TLE4;PRKN;WWOX;TLE1;IGFBP4;SOX13;TMEM170B;MCC;SFRP4;BMP2;DAB2;RNF213;WNT11;TMEM88;GRB10;IGFBP6
(GO:0018212)	9/54	0.000639435	0.047407362	4.262192394	31.34810789	FER;EFEMP1;SYK;KDR;FYN;TEK;EPHA1;EGFR;DDR2
Positive Regulation Of Endothelial Cell Proliferation (GO:0001938)	11/77	0.00065349	0.047407362	3.555866966	26.07582212	BMP4;BMP2;CCL11;ITGB3;KDR;PRKCA;ANG;VEGFD;TEK;FGF2;JCAD
Positive Regulation Of Cell Population Proliferation (GO:0008284)	38/483	0.000669079	0.047407362	1.841335325	13.4594405	HDAC4;PID1;TNXB;CSF1;LAMC2;NOD2;MST1R;RBPJ;FGF2;EGFR;DPP4;GRK5;PDGFD;NAMPT;KDR;HAS2;WNT2;NTRK2;OSR2;EDN1;TSLP;IL15;GLP2R;FN1;IL31RA;IL18;VEGFD;BMP6;BST2;BMP4;BMP2;FER;NR4A3;FGF18;HPSE;EPHA1;ZFPM2;DDR2
Angiogenesis Involved In Wound Healing (GO:0060055)	4/10	0.00069663	0.047407362	14.15721172	102.9123975	ITGB3;KDR;HPSE;CD34
Positive Regulation Of Interleukin-13 Production (GO:0032736)	4/10	0.00069663	0.047407362	14.15721172	102.9123975	TSLP;IRF4;IL18;LGALS9
Synthase Biosynthetic Process (GO:0051770)	4/10	0.00069663	0.047407362	14.15721172	102.9123975	NAMPT;KDR;NOD2;TLR4
Steroid Hormone Mediated Signaling Pathway (GO:0043401)	4/10	0.00069663	0.047407362	14.15721172	102.9123975	BMP4;MAPK1;BMP7;ESR1
Protein Signal Transduction (GO:0060391)	5/17	0.000728886	0.047407362	8.855326652	63.97081559	BMP4;DAB2;RBPMS;BMP7;BMP6
Prostaglandin Biosynthetic Process (GO:0001516)	5/17	0.000728886	0.047407362	8.855326652	63.97081559	EDN1;PLA2G4A;PTGS2;DAGLB;PTGS1
Regulation Of Cartilage Development (GO:0061035)	5/17	0.000728886	0.047407362	8.855326652	63.97081559	BMP4;BMP2;WNT11;ZBTB16;SOX5
Negative Regulation Of Macromolecule Metabolic Process (GO:0010605)	19/186	0.000780191	0.049729375	2.436326442	17.43428327	NOTCH2;PRKN;EDN1;PRKAA2;TIPARP;CITED2;ROCK2;PARP14;KLF4;FGF2;ESR1;BMP4;BMP2;ATOH8;KDR;PGR;LGALS9;CD34;OPTN

Positive Regulation Of Vasculature Development (GO:1904018)	13/104	0.000812183	0.049824096	3.050722311	21.70828321	RAMP2;ANXA3;GATA6;PRKCA;VEGFD;FGF2;STIM1;FGF18;KDR;EMILIN2;TEK;EPHA1;CD34
Positive Regulation Of Phosphorylation (GO:0042327)	22/231	0.000812944	0.049824096	2.256765637	16.05654483	NTRK2;LIMCH1;ROCK2;ITGB3;VEGFD;MST1R;EFNA5;FGF2;EGFR;C3;BMP4;BMP2;DAB2;SLCO3A1;AXL;FGF18;KDR;ANG;TEK;EPHA1;MET;DDR2

Supplementary Table 9: Significant reactome pathway terms for marker genes in fibroblast subcluster2

Term	Overlap	P.value	Adjusted.P.value	Odds.Ratio	Combined.Score	Genes
RHO GTPase Cycle R-HSA-9012999	45/441	3.0722E-07	0.000339785	2.47682242	37.14169065	MTMR1;CPNE8;STEAP3;ROCK2;ARHGEF28;MOSPD2;ARHGAP18;WASL;ADD3;ARHGAP6;UACA;LMNB1;PREX2;AKAP12;DOCK10;SYDE2;ARHGAP22;STK10;ARHGAP42;ARHGAP20;STBD1;MCF2L;MUC13;CKB;SRGAP2;SRGAP1;WASF3;ARHGEF10;RRAS2;MPP7;DIAPH1;ARHGAP10;FMNL2;DAAM1;TAOK3;DLC1;ABI1;RHOJ;RAPGEF1;ARHGEF3;TRIP10;FILIP1;RHOU;DBN1;CDC42SE2
Signal Transduction R-HSA-162582	160/2465	9.88395E-07	0.000546583	1.568782242	21.69183914	CPNE8;STEAP3;AHCYL1;CD80;RBPJ;CXCL13;PREX2;DOCK10;SYDE2;STK10;ARHGAP42;KDR;GRB10;PDK4;PDE8A;RGS6;TLE4;MYLIP;PRKAB2;TLE1;PRKCE;USP2;SOX13;PRKCA;RPGRIP1L;CLIP1;DAAM1;COL4A4;PGR;FKBP5;NOTCH2;MTMR1;CD274;CALCRL;ARHGEF28;ARHGAP18;DTX2;LPAR4;MST1R;ADH7;LTBP1;C3;SPTA1;ARHGAP22;ARHGAP20;PDZD2;GRK5;FLRT2;FLRT3;PDGFD;THEM4;ST3GAL6;SRGAP2;WNT2;SRGAP1;NGFR;WNT10B;SMURF2;GREB1;FN1;GNG12;ESR1;CXCL10;DIAPH1;BMP2;FER;FGF18;PDE3A;ID3;GNRH1;CDC42SE2;GABRB2;ITGB3;PRKAG2;PTPRJ;LAMC2;ADM;WASL;SHB;PCSK6;ADRA1B;ARHGAP6;CHRDL1;LMNB1;SMPD3;AKAP12;GNGT2;C1QTNF1;STBD1;MCF2L;MUC13;PRKG2;PRKG1;JAK1;NCOA2;ARHGEF10;EDN1;ANXA1;KSR1;SYK;ITGA2;GLP2R;RRAS2;PLA2G4A;NGF;ALDH1A3;ARHGAP10;KIF2A;ADORA2B;RHOJ;ALDH1A1;RAPGEF1;ARHGEF3;RARB;RHOU;DAGLB;DBN1;MGLL;HDAC4;KANK1;RAMP2;PRKAA2;CCL11;PHLPP1;LAMA2;ROCK2;MOSPD2;ADD3;TANK;UACA;EGFR;HDAC7;GNG3;WNT11;MAPK1;FYN;CKB;CD55;WASF3;CLASP2;NTRK2;WWOX;VEGFD;PML;MPP7;PDE10A;FMNL2;TAOK3;GPAM;ABHD17B;DHRS9;AXL;DLC1;ABI1;TRIP10;ACKR3;ACKR2;FILIP1;TEK;ACKR1
Interferon Alpha/Beta Signaling R-HSA-909733	13/72	1.77655E-05	0.006549546	4.713273662	51.55497686	RNASEL;RSAD2;ISG15;IFI35;IFIT1;SAMHD1;USP18;IFIT3;IFIT2;BST2;IRF4;IRF7;JAK1

Signaling By Rho GTPases R-HSA-194315	52/644	3.79087E-05	0.010481755	1.9100343	19.44477944	CPNE8;STEAP3;WASL;ARHGAP6;LMNB1;PREX2;AKAP12;DOCK10;SYDE2;STK10;ARHGAP42;STBD1;MCF2L;MUC13;NCOA2;ARHGEF10;RRAS2;PRKCA;ARHGAP10;CLIP1;DAAM1;KIF2A;RHOJ;RAPGEF1;ARHGEF3;RHOU;DBN1;MTMR1;ROCK2;ARHGEF28;MOSPD2;ARHGAP18;ADD3;UACA;ARHGAP22;ARHGAP20;PDZD2;MAPK1;CKB;SRGAP2;SRGAP1;WASF3;CLASP2;MPP7;DIAPH1;FMNL2;TAOK3;DLC1;ABI1;TRIP10;FILIP1;CDC42SE2
Cytokine Signaling In Immune System R-HSA-1280215	55/702	5.18669E-05	0.011472953	1.849519553	18.24889542	CSF1;CD80;PTPRJ;IFI35;IFIT1;IFIT3;IFIT2;LMNB1;GHR;GRB10;LBP;LGALS9;JAK1;GBP6;GBP5;ANXA1;RSAD2;SYK;IL15;FBXW11;IL1R2;IL18;PRKCA;IRAK3;TNFRSF1B;IL17RA;IRF4;RAPGEF1;IRF7;RHOU;RNASEL;SP100;CCL11;NLRC5;NOD2;PTGS2;SAMHD1;TANK;USP18;SOCS2;MAPK9;IL1RL2;PDZD2;MAPK1;FLNB;FYN;TSLP;FN1;IL31RA;ISG15;PML;BST2;CXCL10;TNFSF9;PTPN5
Signaling By Rho GTPases, Miro GTPases And RHOBTB3 R-HSA-9716542	52/660	7.16631E-05	0.013209891	1.858162224	17.73343647	CPNE8;STEAP3;WASL;ARHGAP6;LMNB1;PREX2;AKAP12;DOCK10;SYDE2;STK10;ARHGAP42;STBD1;MCF2L;MUC13;NCOA2;ARHGEF10;RRAS2;PRKCA;ARHGAP10;CLIP1;DAAM1;KIF2A;RHOJ;RAPGEF1;ARHGEF3;RHOU;DBN1;MTMR1;ROCK2;ARHGEF28;MOSPD2;ARHGAP18;ADD3;UACA;ARHGAP22;ARHGAP20;PDZD2;MAPK1;CKB;SRGAP2;SRGAP1;WASF3;CLASP2;MPP7;DIAPH1;FMNL2;TAOK3;DLC1;ABI1;TRIP10;FILIP1;CDC42SE2
RHOD GTPase Cycle R-HSA-9013405	10/52	9.422E-05	0.014886767	5.08052045	47.09580292	AKAP12;CPNE8;DIAPH1;STEAP3;STBD1;MOSPD2;FILIP1;ADD3;DBN1;LMNB1
RHOA GTPase Cycle R-HSA-8980692	18/147	0.000115242	0.015932178	2.990618841	27.12036134	ARHGEF10;ROCK2;ARHGEF28;ARHGAP18;ARHGAP6;PREX2;ARHGAP22;STK10;DIAPH1;ARHGAP10;ARHGAP42;ARHGAP20;DAAM1;MCF2L;STBD1;DLC1;ARHGEF3;SRGAP1
Extracellular Matrix Organization R-HSA-1474244	27/291	0.000334954	0.041162135	2.198746887	17.59331083	TNXB;LAMA2;PCOLCE2;COL14A1;ITGB3;NTN4;LAMC2;NID1;LTBP1;ADAMTS5;EFEMP1;SPOCK3;KDR;TIMP2;DMD;ITGB7;ITGA2;FN1;PRKCA;BMP7;BMP4;BMP2;COL4A4;TLL1;MATN4;DDR2;FBN1
CDC42 GTPase Cycle R-HSA-9013148	17/149	0.000411897	0.042641162	2.756729256	21.4879831	CPNE8;ARHGEF10;STEAP3;WASL;DOCK10;PREX2;ARHGAP22;ARHGAP10;ARHGAP42;ARHGAP20;FMNL2;DAAM1;MCF2L;DLC1;SRGAP2;SRGAP1;CDC42SE2
Non-integrin membrane-ECM Interactions R-HSA-3000171	8/41	0.000424098	0.042641162	5.163771796	40.09950309	LAMA2;ITGB3;ITGA2;NTN4;LAMC2;DMD;PRKCA;DDR2

Supplementary Table 10: Top 25 significantly enriched Gene Ontology Biological Processes terms for genes differentially expressed in Mfap5+/+ mouse fibroblasts vs Mfap5-/- mouse fibroblasts in NS (day 0 post-wounding)

Term	Overlap	P.value	Adjusted.P.value	Odds.Ratio	Combined.Score	Genes
Cytoplasmic Translation (GO:0002181)	53/93	9.61881E-76	9.75347E-73	119.6693807	20670.82085	RPL30;RPL32;RPL31;RPL34;RPLP1;RPS15;RPS14;RPS17;RPS16;RPL18A;RPS19;RPL36AL;RPS18;RPL36;RPL35;RPLP2;RPL38;RPL37;RPL39;RPS13;RPS9;RPS8;RPS5;RPL22;RPS6;RPL13A;RPL37A;RPL24;RPL27;RPL26;RPL28;UBA52;RPL10;RPL11;RPS15A;RPS27A;RPL17;RWDD1;RPL41;RPL35A;RPL23A;RPS26;RPS25;RPS28;RPS27;RPS29;RPL27A;RPL22L1;RPS20;FAU;RPS21;RPS24;RPS23
Peptide Biosynthetic Process (GO:0043043)	54/158	1.24816E-61	5.37798E-59	46.95808224	6585.214678	RPL30;MRPS16;RPL32;RPL31;RPL34;RPLP1;RPS15;RPS14;RPS17;RPS16;RPL18A;RPS19;RPL36AL;RPS18;RPL36;RPL35;RPLP2;RPL38;RPL37;RPL39;RPS13;RPS9;RPS8;RPS5;RPL22;RPS6;MRPS21;RPL13A;MRPL52;RPL37A;RPL24;RPL27;RPL26;RPL28;RPL10;RPL11;RRBP1;RPS15A;RPS27A;RPL17;RPL41;RPL35A;RPL23A;RPS26;RPS25;RPS28;RPS27;RPS29;RPL27A;RPS20;FAU;RPS21;RPS24;RPS23
Translation (GO:0006412)	61/234	1.59112E-61	5.37798E-59	32.8355629	4596.757273	RPL30;MRPS16;RPL32;RPL31;RPL34;RPLP1;MRPL33;RPS15;RPS14;RPS17;RPS16;RPL18A;RPS19;RPL36AL;RPS18;RPL36;RPL35;RPLP2;RPL38;RPL37;RPL39;RPS13;RPS9;RPS8;RPS5;RPL22;RPS6;MRPS21;RPL13A;MRPL52;LARS2;RPL37A;RPL24;RPL27;RPL26;RPL28;UBA52;RPL10;RPL11;RRBP1;MRPL54;RPS15A;RPS27A;RPL17;RWDD1;RPL41;NDUFA7;RPL35A;RPL23A;RPS26;RPS25;RPS28;RPS27;RPS29;RPL27A;RPS20;RPL22L1;FAU;RPS21;RPS24;RPS23
Macromolecule Biosynthetic Process (GO:0009059)	54/183	1.29076E-57	3.27209E-55	37.80945236	4952.740181	RPL30;MRPS16;RPL32;RPL31;RPL34;RPLP1;RPS15;RPS14;RPS17;RPS16;RPL18A;RPS19;RPL36AL;RPS18;RPL36;RPL35;RPLP2;RPL38;RPL37;RPL39;RPS13;RPS9;RPS8;RPS5;RPL22;RPS6;MRPS21;RPL13A;MRPL52;RPL37A;RPL24;RPL27;RPL26;RPL28;RPL10;RPL11;RRBP1;RPS15A;RPS27A;RPL17;RPL41;RPL35A;RPL23A;RPS26;RPS25;RPS28;RPS27;RPS29;RPL27A;RPS20;FAU;RPS21;RPS24;RPS23
Gene Expression (GO:0010467)	57/296	2.74537E-49	5.56761E-47	21.72076018	2428.744911	RPL30;MRPS16;RPL32;RPL31;RPL34;RPLP1;RPS15;RPS14;RPS17;RPS16;RPS19;RPL18A;RPL36AL;RPS18;RPL36;RPL35;RPLP2;RPL38;RPL37;RPL39;RPS13;TGM2;RPS9;RPS8;RPS5;RPL22;RPS6;MRPS21;RPL13A;MRPL52;RPL37A;RPL24;SRSF3;RPL27;RPL26;RPL28;RPL10;RPL11;RRBP1;RPS15A;RPS27A;RPL17;RPL41;RPL35A;RPL23A;RPS26;RPS25;RPS28;RPS27;RPS29;RPL27A;HNRNPA2B1;RPS20;FAU;RPS21;RPS24;RPS23
Cellular Respiration (GO:0045333)	37/85	5.65944E-47	9.56445E-45	64.83235399	6903.879062	NDUFB9;COX7B;NDUFA13;NDUFB7;NDUFA11;NDUFB6;UQCRB;NDUFB11;COX4I1;NDUFB4;NDUFB3;NDUFB2;COX7A2;UQCR11;ETFB;UQCR10;COX6A1;COX5B;COX7C;UQCRH;BLOC1S1;NDUFV3;COX8A;NDUFA7;NDUFA6;NDUFA5;NDUFA4;NDUFA3;NDUFA2;NDUFA1;NDUFC1;COX6C;COX6B1;NDUFS7;NDUFS6;UQCRQ;NDUFS5
Aerobic Electron Transport Chain (GO:0019646)	32/68	4.21176E-42	6.10104E-40	73.24221292	6977.836334	NDUFB9;COX7B;NDUFB7;NDUFB6;UQCRB;COX4I1;NDUFB4;NDUFB3;NDUFB2;COX7A2;UQCR11;UQCR10;COX6A1;COX5B;COX7C;UQCRH;NDUFV3;COX8A;NDUFA7;NDUFA6;NDUFA5;NDUFA4;NDUFA3;NDUFA2;NDUFA1;NDUFC1;COX6C;COX6B1;NDUFS7;NDUFS6;UQCRQ;NDUFS5
Mitochondrial ATP Synthesis Coupled Electron Transport (GO:0042775)	32/70	1.41358E-41	1.79171E-39	69.38031271	6525.902955	NDUFB9;COX7B;NDUFB7;NDUFB6;UQCRB;COX4I1;NDUFB4;NDUFB3;NDUFB2;COX7A2;UQCR11;UQCR10;COX6A1;COX5B;COX7C;UQCRH;NDUFV3;COX8A;NDUFA7;NDUFA6;NDUFA5;NDUFA4;NDUFA3;NDUFA2;NDUFA1;NDUFC1;COX6C;COX6B1;NDUFS7;NDUFS6;UQCRQ;NDUFS5
Aerobic Respiration (GO:0009060)	23/59	3.92553E-28	4.42276E-26	50.73241487	3201.463025	NDUFB9;NDUFA13;NDUFA7;NDUFB7;NDUFA6;NDUFA11;NDUFB6;UQCRB;NDUFA5;NDUFB11;NDUFB4;NDUFA3;NDUFB3;NDUFA2;NDUFB2;NDUFA1;NDUFC1;UQCRH;NDUFS7;NDUFS6;BLOC1S1;NDUFS5;NDUFV3
Oxidative Phosphorylation (GO:0006119)	22/63	1.08389E-25	1.09906E-23	42.42687824	2438.869727	NDUFB9;NDUFA13;NDUFA7;NDUFB7;NDUFA6;NDUFA11;NDUFB6;UQCRB;NDUFA5;NDUFB11;NDUFB4;NDUFA3;NDUFB3;NDUFA2;NDUFB2;NDUFA1;NDUFC1;UQCRH;NDUFS7;NDUFS6;NDUFS5;NDUFV3
Proton Motive Force-Driven Mitochondrial ATP Synthesis (GO:0042776)	20/53	2.90933E-24	2.68187E-22	47.5576482	2577.344865	NDUFB9;NDUFA13;NDUFA7;NDUFB7;NDUFA6;NDUFA11;NDUFB6;NDUFA5;NDUFB11;NDUFB4;NDUFA3;NDUFB3;NDUFA2;NDUFB2;NDUFA1;NDUFC1;NDUFS7;NDUFS6;NDUFS5;NDUFV3

Mitochondrial Electron Transport, NADH To Ubiquinone (GO:0006120)	17/34	2.00308E-23	1.6926E-21	77.60629921	4056.075617	NDUFB9;NDUFA7;NDUFB7;NDUFA6;NDUFB6;NDUFA5;NDUFA4;NDUFB4;NDUFA3;NDUFB3;NDUFA2;NDUFB2;NDUFC1;NDUFS7;NDUFS6;NDUFS5;NDUFV3
Proton Motive Force-Driven ATP Synthesis (GO:0015986)	20/60	5.54026E-23	4.32141E-21	39.22111554	2009.980778	NDUFB9;NDUFA13;NDUFA7;NDUFB7;NDUFA6;NDUFA11;NDUFB6;NDUFA5;NDUFB11;NDUFB4;NDUFA3;NDUFB3;NDUFA2;NDUFB2;NDUFA1;NDUFC1;NDUFS7;NDUFS6;NDUFS5;NDUFV3
NADH Dehydrogenase Complex Assembly (GO:0010257)	18/53	5.67558E-21	3.83669E-19	40.03297572	1866.261863	NDUFB9;NDUFA13;NDUFB7;NDUFA6;NDUFA11;NDUFB6;NDUFA5;NDUFB11;NDUFB4;NDUFA3;NDUFB3;NDUFA2;NDUFB2;NDUFA1;NDUFC1;NDUFAF8;NDUFS7;NDUFS5
Mitochondrial Respiratory Chain Complex I Assembly (GO:0032981)	18/53	5.67558E-21	3.83669E-19	40.03297572	1866.261863	NDUFB9;NDUFA13;NDUFB7;NDUFA6;NDUFA11;NDUFB6;NDUFA5;NDUFB11;NDUFB4;NDUFA3;NDUFB3;NDUFA2;NDUFB2;NDUFA1;NDUFC1;NDUFAF8;NDUFS7;NDUFS5
Mitochondrial Respiratory Chain Complex Assembly (GO:0033108)	20/90	4.69589E-19	2.97602E-17	22.3779169	944.4024564	NDUFB9;NDUFA13;NDUFB7;NDUFA6;NDUFA11;NDUFB6;NDUFA5;NDUFB11;UQCC2;NDUFB4;NDUFA3;COX17;NDUFB3;NDUFA2;NDUFB2;NDUFA1;NDUFC1;NDUFAF8;NDUFS7;NDUFS5
Energy Derivation By Oxidation Of Organic Compounds (GO:0015980)	15/43	6.97082E-18	4.15789E-16	41.22698103	1628.663564	COX8A;COX7B;UQCRB;NDUFA4;COX4I1;COX7A2;UQCR11;UQCR10;COX5B;COX6A1;COX6C;COX7C;UQCRH;COX6B1;UQCRQ
Ribonucleoprotein Complex Biogenesis (GO:0022613)	21/118	7.98069E-18	4.49579E-16	17.00090722	669.3173275	RPS9;RPS8;RPS5;RPS6;RPL11;RPL35A;RPS15;RPS14;RPS25;RPS17;RPS28;RPS16;RPS27;RPS15A;RPS19;RPL38;RPL26;RBIS;RPS13;RPS24;RPS23
Ribosome Biogenesis (GO:0042254)	22/155	1.951E-16	1.04122E-14	13.01784582	470.8947772	RPS9;RPS8;RPS5;RPS6;RPL11;RPL35A;RPS15;RPS14;RPS25;RPS17;RPS28;RPS16;RPS27;RPS15A;RPS19;RPL27;PIN4;RPL26;RBIS;RPS13;RPS24;RPS23
Ribosomal Small Subunit Biogenesis (GO:0042274)	17/84	1.19952E-15	6.08156E-14	19.64120343	674.8100077	RPS9;RPS8;RPS5;RPS6;RPS27L;RPS15;RPS14;RPS25;RPS17;RPS28;RPS16;RPS15A;RPS27;RPS19;RPS13;RPS24;RPS23
Mitochondrial Electron Transport, Cytochrome C To Oxygen (GO:0006123)	10/16	1.31694E-15	6.35896E-14	125.945083	4315.31459	COX8A;COX7B;NDUFA4;COX4I1;COX7A2;COX5B;COX6A1;COX6C;COX7C;COX6B1
rRNA Processing (GO:0006364)	16/101	4.73373E-13	2.18182E-11	14.50076125	411.5155528	RPS6;RPL11;RPL35A;RPS15;RPS14;RPS25;RPS17;RPS28;NSA2;RPS16;RPS27;RPS19;RPL27;PIN4;RPL26;RPS24
rRNA Metabolic Process (GO:0016072)	14/91	2.11693E-11	9.33291E-10	13.90307747	341.7163404	RPS6;RPL11;RPL35A;RPS15;RPS25;RPS17;RPS28;RPS16;RPS27;RPS19;RPL27;PIN4;RPL26;RPS24
ncRNA Processing (GO:0034470)	14/100	7.85613E-11	3.31921E-09	12.4424034	289.4991723	RPS6;RPL11;RPL35A;RPS15;RPS25;RPS17;RPS28;RPS16;RPS27;RPS19;RPL27;PIN4;RPL26;RPS24
protein-RNA Complex Assembly (GO:0022618)	16/150	2.24379E-10	9.1008E-09	9.175299971	203.8539368	RPL10;RPS5;RPL11;RPS27L;RPL23A;RPS15;RPS14;RPS28;RPS27;RPS19;SNRPD2;SNRPG;RPL38;SNRPE;SNRPF;EIF3C

Supplementary Table 11: Top 25 significantly enriched Reactome terms for genes differentially expressed in Mfap5+/+ mouse fibroblasts vs Mfap5-/- mouse fibroblasts in NS (day 0 post-wounding)

Term	Overlap	P.value	Adjusted.P.value	Old.P.value	Combined.Score	Genes
Peptide Chain Elongation R-HSA-156902	51/86	3.8121E-74	2.58841E-71	0	22051.42042	RPL30;RPL32;RPL31;RPL34;RPLP1;RPS15;RPS14;RPS17;RPS16;RPL18A;RPS19;RPL36AL;RPL36;RPL35;RPLP2;RPL38;RPL37;RPL39;RPS13;RPS12;RPS9;RPS8;RPL23;RPS5;RPL22;RPS6;RPL13A;RPL37A;RPL27;RPL26;RPL28;UBA52;RPL11;RPL36A;RPS27L;RPS15A;RPS27A;RPL41;RPL35A;RPL23A;RPS25;RPS28;RPS27;RPS29;RPL27A;RPL22L1;RPS20;FAU;RPS21;RPS24;RPS23
SRP-dependent Cotranslational Protein Targeting To Membrane R-HSA-1799339	54/108	6.7967E-73	1.28374E-70	0	15066.54114	RPL30;RPL32;RPL31;RPL34;RPLP1;RPS15;RPS14;RPS17;RPS16;RPL18A;RPS19;RPL36AL;SEC61G;RPL36;RPL35;RPLP2;RPL38;RPL37;SEC61B;RPL39;RPS13;RPS12;RPS9;RPS8;RPL23;RPS5;RPL22;RPS6;RPL13A;RPL37A;RPL27;RPL26;RPL28;UBA52;RPN1;RPL11;RPL36A;RPS27L;RPS15A;RPS27A;RPL41;RPL35A;RPL23A;RPS25;RPS28;RPS27;RPS29;RPL27A;RPS20;RPL22L1;FAU;RPS21;RPS24;RPS23
Selenocysteine Synthesis R-HSA-2408557	51/90	1.1344E-72	1.28374E-70	0	19388.59651	RPL30;RPL32;RPL31;RPL34;RPLP1;RPS15;RPS14;RPS17;RPS16;RPL18A;RPS19;RPL36AL;RPL36;RPL35;RPLP2;RPL38;RPL37;RPL39;RPS13;RPS12;RPS9;RPS8;RPL23;RPS5;RPL22;RPS6;RPL13A;RPL37A;RPL27;RPL26;RPL28;UBA52;RPL11;RPL36A;RPS27L;RPS15A;RPS27A;RPL41;RPL35A;RPL23A;RPS25;RPS28;RPS27;RPS29;RPL27A;RPL22L1;RPS20;FAU;RPS21;RPS24;RPS23
Eukaryotic Translation Elongation R-HSA-156842	51/90	1.1344E-72	1.28374E-70	0	19388.59651	RPL30;RPL32;RPL31;RPL34;RPLP1;RPS15;RPS14;RPS17;RPS16;RPL18A;RPS19;RPL36AL;RPL36;RPL35;RPLP2;RPL38;RPL37;RPL39;RPS13;RPS12;RPS9;RPS8;RPL23;RPS5;RPL22;RPS6;RPL13A;RPL37A;RPL27;RPL26;RPL28;UBA52;RPL11;RPL36A;RPS27L;RPS15A;RPS27A;RPL41;RPL35A;RPL23A;RPS25;RPS28;RPS27;RPS29;RPL27A;RPL22L1;RPS20;FAU;RPS21;RPS24;RPS23
Eukaryotic Translation Termination R-HSA-72764	51/90	1.1344E-72	1.28374E-70	0	19388.59651	RPL30;RPL32;RPL31;RPL34;RPLP1;RPS15;RPS14;RPS17;RPS16;RPL18A;RPS19;RPL36AL;RPL36;RPL35;RPLP2;RPL38;RPL37;RPL39;RPS13;RPS12;RPS9;RPS8;RPL23;RPS5;RPL22;RPS6;RPL13A;RPL37A;RPL27;RPL26;RPL28;UBA52;RPL11;RPL36A;RPS27L;RPS15A;RPS27A;RPL41;RPL35A;RPL23A;RPS25;RPS28;RPS27;RPS29;RPL27A;RPL22L1;RPS20;FAU;RPS21;RPS24;RPS23
Viral mRNA Translation R-HSA-192823	51/90	1.1344E-72	1.28374E-70	0	19388.59651	RPL30;RPL32;RPL31;RPL34;RPLP1;RPS15;RPS14;RPS17;RPS16;RPL18A;RPS19;RPL36AL;RPL36;RPL35;RPLP2;RPL38;RPL37;RPL39;RPS13;RPS12;RPS9;RPS8;RPL23;RPS5;RPL22;RPS6;RPL13A;RPL37A;RPL27;RPL26;RPL28;UBA52;RPL11;RPL36A;RPS27L;RPS15A;RPS27A;RPL41;RPL35A;RPL23A;RPS25;RPS28;RPS27;RPS29;RPL27A;RPL22L1;RPS20;FAU;RPS21;RPS24;RPS23

Formation Of A Pool Of Free 40S Subunits R-HSA-72689	52/98	5.2552E-72	4.81027E-70	0	16675.25554	RPL30;RPL32;RPL31;RPL34;RPLP1;RPS15;RPS14;RPS17;RPS16;RPL18A;RPS19;RPL36AL;RPL36;RPL35;RPLP2;RPL38;RPL37;RPL39;RPS13;RPS12;RPS9;RPS8;RPL23;RPS5;RPL22;RPS6;RPL13A;RPL37A;RPL27;RPL26;RPL28;UBA52;RPL11;RPL36A;RPS27L;RPS15A;RPS27A;RPL41;RPL35A;RPL23A;RPS25;RPS28;RPS27;RPS29;RPL27A;RPS20;RPL22L1;FAU;EIF3C;RPS21;RPS24;RPS23
Nonsense Mediated Decay (NMD) Independent Of Exon Junction Complex (EJC) R-HSA-975956	51/92	5.6675E-72	4.81027E-70	0	18261.86522	RPL30;RPL32;RPL31;RPL34;RPLP1;RPS15;RPS14;RPS17;RPS16;RPL18A;RPS19;RPL36AL;RPL36;RPL35;RPLP2;RPL38;RPL37;RPL39;RPS13;RPS12;RPS9;RPS8;RPL23;RPS5;RPL22;RPS6;RPL13A;RPL37A;RPL27;RPL26;RPL28;UBA52;RPL11;RPL36A;RPS27L;RPS15A;RPS27A;RPL41;RPL35A;RPL23A;RPS25;RPS28;RPS27;RPS29;RPL27A;RPL22L1;RPS20;FAU;RPS21;RPS24;RPS23
Response Of EIF2AK4 (GCN2) To Amino Acid Deficiency R-HSA-9633012	51/98	5.2079E-70	3.92908E-68	0	15486.85905	RPL30;RPL32;RPL31;RPL34;RPLP1;RPS15;RPS14;RPS17;RPS16;RPL18A;RPS19;RPL36AL;RPL36;RPL35;RPLP2;RPL38;RPL37;RPL39;RPS13;RPS12;RPS9;RPS8;RPL23;RPS5;RPL22;RPS6;RPL13A;RPL37A;RPL27;RPL26;RPL28;UBA52;RPL11;RPL36A;RPS27L;RPS15A;RPS27A;RPL41;RPL35A;RPL23A;RPS25;RPS28;RPS27;RPS29;RPL27A;RPS20;RPL22L1;FAU;RPS21;RPS24;RPS23
L13a-mediated Translational Silencing Of Ceruloplasmin Expression R-HSA-156827	52/108	5.1325E-69	3.485E-67	0	13116.3343	RPL30;RPL32;RPL31;RPL34;RPLP1;RPS15;RPS14;RPS17;RPS16;RPL18A;RPS19;RPL36AL;RPL36;RPL35;RPLP2;RPL38;RPL37;RPL39;RPS13;RPS12;RPS9;RPS8;RPL23;RPS5;RPL22;RPS6;RPL13A;RPL37A;RPL27;RPL26;RPL28;UBA52;RPL11;RPL36A;RPS27L;RPS15A;RPS27A;RPL41;RPL35A;RPL23A;RPS25;RPS28;RPS27;RPS29;RPL27A;RPS20;RPL22L1;FAU;EIF3C;RPS21;RPS24;RPS23
GTP Hydrolysis And Joining Of 60S Ribosomal Subunit R-HSA-72706	52/109	9.7098E-69	5.9936E-67	0	12833.32414	RPL30;RPL32;RPL31;RPL34;RPLP1;RPS15;RPS14;RPS17;RPS16;RPL18A;RPS19;RPL36AL;RPL36;RPL35;RPLP2;RPL38;RPL37;RPL39;RPS13;RPS12;RPS9;RPS8;RPL23;RPS5;RPL22;RPS6;RPL13A;RPL37A;RPL27;RPL26;RPL28;UBA52;RPL11;RPL36A;RPS27L;RPS15A;RPS27A;RPL41;RPL35A;RPL23A;RPS25;RPS28;RPS27;RPS29;RPL27A;RPS20;RPL22L1;FAU;EIF3C;RPS21;RPS24;RPS23
Cap-dependent Translation Initiation R-HSA-72737	52/116	6.7588E-67	3.82434E-65	0	11116.06035	RPL30;RPL32;RPL31;RPL34;RPLP1;RPS15;RPS14;RPS17;RPS16;RPL18A;RPS19;RPL36AL;RPL36;RPL35;RPLP2;RPL38;RPL37;RPL39;RPS13;RPS12;RPS9;RPS8;RPL23;RPS5;RPL22;RPS6;RPL13A;RPL37A;RPL27;RPL26;RPL28;UBA52;RPL11;RPL36A;RPS27L;RPS15A;RPS27A;RPL41;RPL35A;RPL23A;RPS25;RPS28;RPS27;RPS29;RPL27A;RPS20;RPL22L1;FAU;EIF3C;RPS21;RPS24;RPS23
Nonsense Mediated Decay (NMD) Enhanced By Exon Junction Complex (EJC) R-HSA-975957	51/112	4.7777E-66	2.49544E-64	0	11242.03443	RPL30;RPL32;RPL31;RPL34;RPLP1;RPS15;RPS14;RPS17;RPS16;RPL18A;RPS19;RPL36AL;RPL36;RPL35;RPLP2;RPL38;RPL37;RPL39;RPS13;RPS12;RPS9;RPS8;RPL23;RPS5;RPL22;RPS6;RPL13A;RPL37A;RPL27;RPL26;RPL28;UBA52;RPL11;RPL36A;RPS27L;RPS15A;RPS27A;RPL41;RPL35A;RPL23A;RPS25;RPS28;RPS27;RPS29;RPL27A;RPS20;RPL22L1;FAU;RPS21;RPS24;RPS23
Selenoamino Acid Metabolism R-HSA-2408522	51/114	1.542E-65	7.47848E-64	0	10799.25048	RPL30;RPL32;RPL31;RPL34;RPLP1;RPS15;RPS14;RPS17;RPS16;RPL18A;RPS19;RPL36AL;RPL36;RPL35;RPLP2;RPL38;RPL37;RPL39;RPS13;RPS12;RPS9;RPS8;RPL23;RPS5;RPL22;RPS6;RPL13A;RPL37A;RPL27;RPL26;RPL28;UBA52;RPL11;RPL36A;RPS27L;RPS15A;RPS27A;RPL41;RPL35A;RPL23A;RPS25;RPS28;RPS27;RPS29;RPL27A;RPS20;RPL22L1;FAU;RPS21;RPS24;RPS23

Influenza Viral RNA Transcription And Replication R-HSA-168273	53/137	6.4025E-64	2.89822E-62	0	8273.346853	RPL30;RPL32;RPL31;RPL34;RPLP1;RPS15;RPS14;RPS17;RPS16;RPL18A;RPS19;RPL36AL;RPL36;RPL35;RPLP2;RPL38;RPL37;RPL39;RPS13;RPS12;RPS9;RPS8;RPL23;RPS5;RPL22;RPS6;RPL13A;RPL37A;RPL27;RPL26;RPL28;UBA52;RPL11;RPL36A;RPS27L;RPS15A;POLR2F;RPS27A;POLR2K;RPL41;RPL35A;RPL23A;RPS25;RPS28;RPS27;RPS29;RPL27A;RPS20;RPL22L1;FAU;RPS21;RPS24;RPS23
Regulation Of Expression Of SLITs And ROBOs R-HSA-9010553	56/167	2.1186E-63	8.99101E-62	0	6643.302258	RPL30;RPL32;RPL31;RPL34;RPLP1;RPS15;RPS14;RPS17;RPS16;RPL18A;RPS19;RPL36AL;RPL36;RPL35;RPLP2;RPL38;ELOB;RPL37;RPL39;RPS13;RPS12;RPS9;RPS8;RPL23;RPS5;RPL22;RPS6;RPL13A;RBX1;RPL37A;PSME1;RPL27;RPL26;RPL28;UBA52;RPL11;RPL36A;RPS27L;PSMB6;RPS15A;RPS27A;RPL41;RPL35A;RPL23A;SEM1;RPS25;RPS28;RPS27;RPS29;RPL27A;RPS20;RPL22L1;FAU;RPS21;RPS24;RPS23
Influenza Infection R-HSA-168255	54/157	8.3044E-62	3.31687E-60	0	6668.808311	RPL30;RPL32;RPL31;RPL34;RPLP1;RPS15;RPS14;RPS17;RPS16;RPL18A;RPS19;RPL36AL;RPL36;RPL35;RPLP2;RPL38;RPL37;RPL39;RPS13;RPS12;RPS9;RPS8;RPL23;RPS5;RPL22;RPS6;RPL13A;RPL37A;RPL27;RPL26;RPL28;UBA52;RPL11;RPL36A;RPS27L;RPS15A;POLR2F;RPS27A;POLR2K;RPL41;RPL35A;RPL23A;RPS25;RPS28;RPS27;RPS29;RPL27A;RPS20;RPL22L1;FAU;RPS21;RPS24;RPS23;HSPA1A
Cellular Response To Starvation R-HSA-9711097	51/153	1.6426E-57	6.19631E-56	0	5832.388806	RPL30;RPL32;RPL31;RPL34;RPLP1;RPS15;RPS14;RPS17;RPS16;RPL18A;RPS19;RPL36AL;RPL36;RPL35;RPLP2;RPL38;RPL37;RPL39;RPS13;RPS12;RPS9;RPS8;RPL23;RPS5;RPL22;RPS6;RPL13A;RPL37A;RPL27;RPL26;RPL28;UBA52;RPL11;RPL36A;RPS27L;RPS15A;RPS27A;RPL41;RPL35A;RPL23A;RPS25;RPS28;RPS27;RPS29;RPL27A;RPS20;RPL22L1;FAU;RPS21;RPS24;RPS23
Signaling By ROBO Receptors R-HSA-376176	56/209	3.986E-57	1.42448E-55	0	4327.855293	RPL30;RPL32;RPL31;RPL34;RPLP1;RPS15;RPS14;RPS17;RPS16;RPL18A;RPS19;RPL36AL;RPL36;RPL35;RPLP2;RPL38;ELOB;RPL37;RPL39;RPS13;RPS12;RPS9;RPS8;RPL23;RPS5;RPL22;RPS6;RPL13A;RBX1;RPL37A;PSME1;RPL27;RPL26;RPL28;UBA52;RPL11;RPL36A;RPS27L;PSMB6;RPS15A;RPS27A;RPL41;RPL35A;RPL23A;SEM1;RPS25;RPS28;RPS27;RPS29;RPL27A;RPS20;RPL22L1;FAU;RPS21;RPS24;RPS23
Translation R-HSA-72766	61/281	3.2683E-56	1.10959E-54	0	3290.935976	RPL30;MRPS16;RPL32;RPL31;RPL34;RPLP1;MRPL33;RPS15;RPS14;RPS17;RPS16;RPS19;RPL18A;RPL36AL;SEC61G;RPL36;RPL35;RPLP2;RPL38;RPL37;SEC61B;RPL39;RPS13;RPS12;RPS9;RPS8;RPL23;RPS5;RPL22;RPS6;MRPS21;RPL13A;MRPL52;LARS2;RPL37A;RPL27;RPL26;UBA52;RPL28;RPN1;RPL11;RPL36A;RPS27L;MRPL54;RPS15A;RPS27A;RPL41;RPL35A;RPL23A;RPS25;RPS28;RPS27;RPS29;RPL27A;RPS20;RPL22L1;FAU;EIF3C;RPS21;RPS24;RPS23
Major Pathway Of rRNA Processing In Nucleolus And Cytosol R-HSA-6791226	51/179	1.7165E-53	5.54998E-52	0	4313.008751	RPL30;RPL32;RPL31;RPL34;RPLP1;RPS15;RPS14;RPS17;RPS16;RPL18A;RPS19;RPL36AL;RPL36;RPL35;RPLP2;RPL38;RPL37;RPL39;RPS13;RPS12;RPS9;RPS8;RPL23;RPS5;RPL22;RPS6;RPL13A;RPL37A;RPL27;RPL26;RPL28;UBA52;RPL11;RPL36A;RPS27L;RPS15A;RPS27A;RPL41;RPL35A;RPL23A;RPS25;RPS28;RPS27;RPS29;RPL27A;RPS20;RPL22L1;FAU;RPS21;RPS24;RPS23

rRNA Processing In Nucleus And Cytosol R-HSA-8868773	51/189	3.9166E-52	1.20881E-50	0	3895.504433	RPL30;RPL32;RPL31;RPL34;RPLP1;RPS15;RPS14;RPS17;RPS16;RPL18A;RPS19;RPL36AL;RPL36;RPL35;RPLP2;RPL38;RPL37;RPL39;RPS13;RPS12;RPS9;RPS8;RPL23;RPS5;RPL22;RPS6;RPL13A;RPL37A;RPL27;RPL26;RPL28;UBA52;RPL11;RPL36A;RPS27L;RPS15A;RPS27A;RPL41;RPL35A;RPL23A;RPS25;RPS28;RPS27;RPS29;RPL27A;RPS20;RPL22L1;FAU;RPS21;RPS24;RPS23
rRNA Processing R-HSA-72312	51/199	7.3552E-51	2.17137E-49	0	3540.491561	RPL30;RPL32;RPL31;RPL34;RPLP1;RPS15;RPS14;RPS17;RPS16;RPL18A;RPS19;RPL36AL;RPL36;RPL35;RPLP2;RPL38;RPL37;RPL39;RPS13;RPS12;RPS9;RPS8;RPL23;RPS5;RPL22;RPS6;RPL13A;RPL37A;RPL27;RPL26;RPL28;UBA52;RPL11;RPL36A;RPS27L;RPS15A;RPS27A;RPL41;RPL35A;RPL23A;RPS25;RPS28;RPS27;RPS29;RPL27A;RPS20;RPL22L1;FAU;RPS21;RPS24;RPS23
Cellular Responses To Stress R-HSA-2262752	71/722	4.6028E-41	1.30221E-39	0	966.2716843	RPL30;COX7B;RPL32;RPL31;RPL34;RPLP1;COX4I1;COX6A1;COX7C;RPS15;RPS14;RPS17;RPS16;RPS19;RPL18A;RPL36AL;RPL36;RPL35;RPLP2;RPL38;ELOB;RPL37;RPL39;RPS13;RPS12;COX8A;RPS9;RPS8;RPL23;RPS5;RPL22;RPS6;RPL13A;CYBA;COX6B1;RBX1;RPL37A;PSME1;RPL27;RPL26;UBA52;RPL28;RPL11;RPL36A;RPS27L;COX5B;PSMB6;RPS15A;SERPINH1;RPS27A;RPL41;JUN;RPL35A;RPL23A;COX6C;ATOX1;SOD1;SEM1;RPS25;RPS28;RPS27;RPS29;RPL27A;RPS20;RPL22L1;FAU;RPS21;RPS24;HSPA1B;RPS23;HSPA1A
Respiratory Electron Transport R-HSA-611105	34/90	1.3346E-40	3.62473E-39	0	4627.279706	NDUFB9;COX7B;NDUFA13;NDUFB7;NDUFA11;NDUFB6;UQCRB;NDUFB11;COX4I1;NDUFB4;NDUFB3;NDUFB2;UQCR11;ETFB;UQCR10;COX6A1;COX5B;COX7C;UQCRH;NDUFV3;COX8A;NDUFA7;NDUFA6;NDUFA5;NDUFA3;NDUFA2;NDUFA1;NDUFC1;COX6C;COX6B1;NDUFS7;NDUFS6;UQCRQ;NDUFS5

Supplementary Table 12: Top 25 significantly enriched Gene Ontology Biological Processes terms for genes differentially expressed in Mfap5-/- mouse fibroblasts vs Mfap5+/+ mouse fibroblasts in NS (day 0 post-wounding)

Term	Overlap	P.value	Adjusted.P.value	Odds.Ratio	Combined.Score	Genes
Protein Modification Process (GO:0036211)	136/711	1.0877E-15	4.6185E-12	2.343946488	80.75993608	APP;GMFB;CCNT1;MAML1;UBE2D3;PPP2R2A;PLAT;LOXL1;LOXL2;DCAF1;STK10;PTAR1;TBK1;RPS6KA5;AKT3;PIM3;MACROD2;LARGE1;MAP3K5;CSNK1G3;PRKCI;MAP2K1;MORC3;PPTC7;DAPK1;FBXW11;IGFBP3;PRKCD;COG3;ATG10;DYRK1A;LMTK2;PRKCA;PPP4R3A;FBXO11;ERN1;LOX;ADPRH;SIK3;SIK2;TRIB1;PXK;MTMR3;UGCG;GRK5;MAN2A1;STK38;ABL1;MAP3K20;ABL2;MARK4;ST3GAL1;MARK3;ST3GAL2;B3GALNT2;CDK17;UBE2F;UVRAG;TGFB2;YES1;INSR;FN1;OGA;BRAF;SSH2;PTK2;SSH1;FER;WNK1;COPS2;UST;UBA3;CDK14;BMPR1A;FGFR1;ACVRL1;SMG1;BMPR2;DYRK2;MAST2;PTPRM;PTPRJ;PTPRG;PPP6C;PPP2R5E;RB1CC1;TLK2;PRKG2;JAK2;PRKG1;JAK1;ANXA1;PDPK1;IRAK3;TGFBR1;CTTNBP2NL;TGFBR2;LATS1;PPM1A;PPM1B;NPC1;SGK1;B4GALT5;CAMK2D;CCL11;ROCK2;THBS1;RNF2;PPM1G;PPP2CA;PPP2CB;MAPK9;WNT11;CCL2;MAPK1;FYN;MAP4K5;MAPK6;FNIP2;ARFGEF1;USP25;MAP3K1;CSNK1A1;RYK;PTPN11;CDC42BPB;KRT10;CDC42BPA;ACVR2A;HIPK2;TMEM59;TAOK1;QPCT;PKN2;CAMK1;METAP1
Protein Phosphorylation (GO:0006468)	103/500	2.8864E-14	6.1279E-11	2.549656852	79.48852766	ATF2;APP;GMFB;CCNT1;MAML1;IGF1R;STK10;RPS6KA3;TBK1;EFEMP1;RPS6KA5;AKT3;PIM3;MAP3K5;CSNK1G3;PRKCI;MAP2K1;MORC3;DAPK1;IGFBP3;PRKCD;DYRK1A;LMTK2;PRKCA;ERN1;SIK3;SIK2;TRIB1;PXK;TNKS;GRK5;STK38;ABL1;MAP3K20;ABL2;MARK4;MARK3;CDK17;UVRAG;TGFB2;INSR;BRAF;CLK4;PTK2;SMAD7;BMP4;FER;WNK1;COPS2;CDK14;BMPR1A;FGFR1;ACVRL1;SMG1;BMPR2;DYRK2;MAST4;MAST2;EEF2K;RB1CC1;TLK2;PRKG2;ERC1;JAK2;PRKG1;JAK1;PDPK1;IRAK3;TGFBR1;TGFBR2;LATS1;TGFBR3;SGK1;TLR4;DDR2;CAMK2D;USP15;CCL11;CAB39;ROCK2;EGFR;MAPK9;WNT11;CCL2;MAPK1;RICTOR;FYN;MAP4K5;MAPK6;FNIP2;MAP3K1;CSNK1A1;RYK;CDC42BPB;CDC42BPA;ACVR2A;HIPK2;ABI2;RPS6KB1;TAOK1;PKN2;CAMK1;TEK
Ubiquitin-Dependent Protein Catabolic Process (GO:0006511)	83/367	4.8222E-14	6.825E-11	2.858659922	87.6549873	RNF11;RNF13;UBE2D3;OTUD7B;RNF19B;TNFAIP3;HERPUD1;RNF115;TRIM2;RNF19A;ARIH2;UBXN7;RNF111;FBXO9;FBXW11;FBXW7;UBE2E1;AXIN1;RC3H1;UBE4B;FBXO11;RAD23B;PJA2;UBE2R2;KCTD10;PELI1;CLOCK;CHMP5;PRKN;UBA6;CUL3;CUL1;RHOBTB3;UBR4;NEDD4L;UBR3;AMBRA1;RCHY1;RNF4;ZNRF1;COP1;HECTD1;ZNRF3;RNF216;PSMB2;RNF217;SPOP;TBL1X;NAE1;RFFL;KLHDC2;NDFIP2;XBP1;UBE2H;NDFIP1;ANKIB1;SMURF2;CSNK1A1;USP9X;SMURF1;FEM1B;FBXL17;WWP1;UBE2G1;BFAR;PML;MIB1;CNOT4;UBE2W;ITCH;SPSB1;NPLOC4;APPBP2;APC;TOLLIP;NEDD4;TTC3;MDM2;UBAP1;TRIP12;UBE2K;FBXL7;NFE2L2

Cellular Response To Growth Factor Stimulus (GO:0071363)	47/155	2.7218E-13	2.8891E-10	4.215461018	121.9630987	ACVRL1;RAMP2;BMPR2;SYAP1;HSPB1;NR3C1;ADAMTS12;EGFR;ZFP36L1;TMEM100;RAP1A;HAS1;CCL2;HAS2;CPNE3;PDE8A;WNT2;MAP2K5;WWOX;SMAD4;SMAD3;ANXA1;PDPK1;KIDINS220;MAGI2;PTPN11;KLF4;TGFBR1;ACVR2A;SMAD7;TGFBR2;EEF1A1;ERN1;BMP4;EHD1;SETX;SLC4A7;SFRP1;RPS6KB1;PDE3A;RAPGEF1;GAS1;RAPGEF2;CD44;BMPR1A;LGMN;LIMS1
Phosphorylation (GO:0016310)	88/429	2.8667E-12	2.4344E-09	2.523092227	67.05835847	ACVRL1;APP;SMG1;GMFB;BMPR2;PANK3;DYRK2;CCNT1;CERK;MAML1;MAST2;LIMD1;STK10;TBK1;RPS6KA5;RB1CC1;AKT3;TLK2;PRKG2;PIM3;JAK2;PRKG1;JAK1;MAP3K5;PRKCI;MAP2K1;MORC3;DAPK1;PDPK1;IGFBP3;PRKCD;DYRK1A;LMTK2;PRKCA;IRAK3;TGFBR1;TGFBR2;ERN1;LATS1;PIK3CA;SIK3;SIK2;TRIB1;SGK1;PXK;CAMK2D;CCL11;ROCK2;MAPK9;WNT11;GRK5;STK38;ABL1;MAP3K20;CCL2;MAPK1;RICTOR;FYN;MAP4K5;MARK4;MAPK6;FNIP2;MARK3;NADK;CDK17;UVRAG;TGFB2;MAP3K1;CSNK1A1;RYK;INSR;BRAF;CDC42BPB;CDC42BPA;PTK2;ACVR2A;HIPK2;FER;WNK1;TOLLIP;TAOK1;COPS2;PI4KA;PKN2;CAMK1;CDK14;BMPR1A;FGFR1
Protein Polyubiquitination (GO:0000209)	56/226	1.4802E-11	1.0475E-08	3.195365419	79.68052034	UBE2D3;RNF19B;TNFAIP3;UBE3A;NPEPPS;RNF115;UBE2Q1;TRIM2;RNF19A;CDC27;ARIH2;FBXO9;FBXW11;UBE2E3;UBE2E1;RC3H1;UBE2E2;UBE4B;UBE2R2;PELI1;UBE2V2;PRKN;TNKS;CUL3;MYCBP2;PELI2;CUL1;NEDD4L;UBE2J2;RNF4;UBE2J1;ZNRF1;HECTD1;RNF216;RNF217;UBR5;SPOP;RFFL;UBE2H;ANKIB1;SMURF1;HUWE1;FBXL17;LNPEP;UBE2G1;BFAR;UBE2W;ITCH;NEDD4;TTC3;MDM2;UBE2N;TPP2;TRIP12;UBE2K;FBXL7
Protein Ubiquitination (GO:0016567)	86/434	3.4245E-11	2.0772E-08	2.412541254	58.13620082	RNF11;RNF13;UBE2D3;RNF19B;UBE3A;NPEPPS;BABAM2;RNF115;UBE2Q1;TRIM2;RNF19A;ARIH2;RNF111;FBXO9;ATG3;FBXW7;FBXW11;UBE2E1;MSL2;RC3H1;UBE4B;FBXO11;PJA2;UBE2R2;KCTD10;GAN;NFX1;HECW2;UBE2V2;PRKN;UHRF2;TNKS;UBA6;CUL3;PELI2;CUL2;RNF38;MYCBP2;CUL1;NEDD4L;UBR3;DTX2;AMBRA1;KLHL13;RCHY1;UBE2J2;RNF4;RNFT1;UBE2J1;VCPIP1;ZNRF3;UBB;RNF217;UBR5;SPOP;NAE1;UBE2H;ANKIB1;SMURF2;USP9X;SMURF1;FBXL17;HUWE1;WWP1;KLHL2;LNPEP;UBE2G1;BFAR;KLHL24;MIB1;CNOT4;UBE2W;SPSB1;ITCH;KLHL7;NEDD4;OTULIN;MDM2;UBE2N;TPP2;TRIP12;FBXL5;UBE2K;TRIM33;FBXL7;NFE2L2
Protein K48-linked Ubiquitination (GO:0070936)	27/70	6.921E-11	3.6733E-08	6.039213986	141.2805863	PRKN;CUL3;UBE2D3;MYCBP2;CUL1;TNFAIP3;NEDD4L;UBE3A;RNF4;ZNRF1;RNF115;RNF216;UBR5;ARIH2;RFFL;FBXO9;UBE2H;UBE2E3;UBE2E1;UBE2E2;BFAR;UBE2G1;ITCH;UBE2R2;TTC3;PELI1;UBE2K
Proteasomal Protein Catabolic Process (GO:0010498)	52/220	4.9131E-10	2.3179E-07	2.996297439	64.22245295	UBE2D3;TRIM2;DDI2;UBXN7;FBXO9;FBXW11;FBXW7;AXIN1;UBE4B;RAD23B;PJA2;KCTD10;PSME4;PELI1;ATF6;CLOCK;PRKN;CUL3;SEL1L;CUL1;RHOBTB3;NEDD4L;AMBRA1;RNF4;ZNRF1;COP1;HECTD1;RNF216;PSMB2;SPOP;TBL1X;RFFL;KLHDC2;XBP1;SMURF2;CSNK1A1;SMURF1;FEM1B;FBXL17;WWP1;UBE2G1;BFAR;PML;UBE2W;ITCH;SPSB1;APPBP2;APC;MDM2;TRIP12;UBE2K;NFE2L2
Negative Regulation Of Cell Migration (GO:0030336)	42/163	1.426E-09	6.0549E-07	3.350835767	68.25105663	ACVRL1;HDAC2;ARID4B;MIA3;PTPRJ;ADARB1;FGF2;PTPRG;DUSP10;WNT11;HAS1;SAP30L;DPYSL3;DAG1;CYP1B1;EMILIN2;SUDS3;RAC1;SRGAP1;SCAI;CLASP1;CLASP2;TCAF1;IGFBP5;LIMCH1;IGFBP3;MAGI2;MITF;MCC;SULF1;SMAD7;SFRP1;TMEFF2;SFRP2;NAV3;NF1;DPEP1;NF2;TRIB1;RECK;VCL;BMPR1A

Regulation Of Cell Migration (GO:0030334)	81/434	2.4933E-09	9.6243E-07	2.233318674	44.24123939	ACVRL1;CSF1;MIA3;PTPRJ;ARID4B;ADARB1;FGF2;SYNE2;ACTG1;IGF1R;DOCK10;FGF7;DUSP10;PLAU;DPYSL3;DAG1;CYP1B1;EMILIN2;CPNE3;RAC1;PLEKHG3;ANXA1;IGFBP5;LIMCH1;MAGI2;PRKCA;MITF;RHOC;TGFBR1;TMEFF2;SFRP1;ADAM17;SFRP2;KIF2A;NAV3;RUFY3;NUMB;MYADM;ADAM9;DPEP1;SGK1;VCL;MACF1;HDAC2;CCL11;TNXB;SEMA3C;ROCK2;SEMA3E;THBS1;EGFR;SDCBP;WNT11;CCL7;PDGFD;SAP30L;HAS2;FYN;SUDS3;RFFL;SRGAP1;ARSB;SCAI;SPAG9;CARMIL1;XBP1;TCAF1;ACE;YTHDF3;INSR;LAMB1;SULF1;SOD2;PTK2;SMAD7;DAB2;FER;APC;NF1;NF2;RECK
Modification-Dependent Protein Catabolic Process (GO:0019941)	46/192	3.0628E-09	1.0837E-06	3.043853311	59.67149197	PRKN;RNF11;RNF13;UBA6;CUL3;UBE2D3;UBR4;RNF19B;NEDD4L;UBR3;RCHY1;HERPUD1;ZNRF3;UBB;RNF19A;RNF217;ARIH2;NAE1;RFFL;NDFIP2;UBE2H;XBP1;NDFIP1;ANKIB1;SMURF2;SMURF1;UBE2E1;RC3H1;UBE4B;BFAR;UBE2G1;FBXO11;MIB1;CNOT4;ITCH;SPSB1;NPLOC4;UBE2R2;KCTD10;TOLLIP;NEDD4;TTC3;MDM2;TRIP12;UBE2K;FBXL7
Protein Modification By Small Protein Conjugation (GO:0032446)	71/364	3.577E-09	1.1683E-06	2.353539303	45.77340437	UBE2D3;IFIH1;RNF115;SUMO1;SUMO2;ARIH2;RNF111;FBXO9;ATG3;FBXW11;FBXW7;NSMCE2;ATG10;UBE2E1;MSL2;UBE2E2;FBXO11;PIAS2;PJA2;PIAS1;NSMCE4A;KCTD10;GAN;NFX1;HECW2;UBE2V2;PRKN;HDAC4;DCUN1D5;UHRF2;UBA6;DCUN1D3;CUL3;MYCBP2;CUL2;RNF38;CUL1;NEDD4L;UBR3;DTX2;KLHL13;RCHY1;VCPIP1;ZNRF3;UBB;NAE1;RANBP2;UBE2F;SMURF2;USP9X;SMURF1;FBXL17;KLHL2;WWP1;KLHL24;PML;MIB1;CNOT4;ITCH;SPSB1;KLHL7;NEDD4;COPS2;OTULIN;MDM2;UBA3;UBE2N;FBXL5;FBXL7;TRIM33;NFE2L2
Organelle Organization (GO:0006996)	78/418	4.9592E-09	1.5041E-06	2.230796985	42.65733211	RB1;PI4K2B;MAST4;PITPNB;MAST2;TNFAIP3;SNX10;MIA3;WASL;LIMD1;PKD2;PHB2;SIPA1L3;DPYSL2;MYC;PLCE1;TMED2;ATP7A;TMED7;BCAS3;PRKCI;PDCD6IP;DST;STX7;VPS13D;COG3;PRPF40A;TMEM135;KAT6A;YME1L1;RHOU;CHMP5;PRKN;CCL11;ATL3;SAR1B;ATL2;HTT;TOR1AIP2;PRDX3;RHOT1;BAG5;CCL7;PACSIN2;CCL2;DYM;RICTOR;MARK4;RAB6B;EPS15;PDZD8;LNPK;CLASP1;CLASP2;ARFGEF1;YTHDF2;YTHDF3;ATP8B2;CSNK1A1;ATP8B1;MTX2;RANBP9;TRAPPC8;CDC42BPB;LYSMD3;WTIP;TTC17;DIAPH1;GOLPH3;PIKFYVE;NPLOC4;FMNL2;ABI2;VAPA;DNAJA3;RAB18;TMCC1;BRWD3

Positive Regulation Of DNA-templated Transcription (GO:0045893)	179/1243	7.3781E-09	2.0885E-06	1.661070066	31.10311411	ATF1;ATF2;APP;TCERG1;PID1;CCNT2;CRTC3;CCNT1;MAML1;HNRNPU;UBP1;RORA;RORB;RBPJ;NR3C1;FGF2;ETS2;DCAF6;RPS6KA3;FGF7;TBK1;RPS6KA5;MYC;ZC3H12A;CREB3L2;NAMPT;EP300;IER5;JUNB;RNF111;MAP3K5;CDK5RAP2;MEF2A;MAP2K1;FBXW11;MRTFB;UBE2E1;DYRK1A;MRTFA;MITF;SFRP1;SFRP2;KAT6A;ATF7;TRIM16;ZFPM2;ATF6;CLOCK;INO80D;KMT2A;DHX9;TNKS;PSEN1;BCL10;HIF1A;MBTD1;HIVEP1;ABL1;WNT2;SNX5;MAP2K5;FZD1;WWTR1;AKIRIN2;CREBBP;XBP1;AKIRIN1;SMAD4;TGFB2;JUND;POU2F1;SMAD3;JUP;SMARCA5;NR1D2;ESR1;NFKB1;SETX;BMP4;IL6;DAB2;AGO2;MAFF;REL;CREBRF;CDK13;NFE2L2;PICALM;BMPR1A;CDKN1C;ACVRL1;ARF4;CSRNP1;RARG;BMPR2;THRB;ELL;PHF20;TCF20;RSF1;WASL;PKD2;CDC73;GLI3;PHF8;MED13;SFR1;SPIN1;YAF2;TEAD1;YWHAH;ACTR3;SS18;BCAS3;NCOA2;RIPK2;MSL2;TET2;AXIN1;ARNT;VEZF1;PARP9;ZFX;TGFBR1;PPM1A;ELF1;ZEB2;ELF2;MDFIC;ELF4;MED21;IRF2;TET3;WAC;TLR4;RHOQ;CREB5;PRKN;HDAC4;NFAT5;CEBPB;GTF2A2;HDAC2;RNF4;NR2C2;EGFR;RELB;BICRA;NSD3;SCAF8;WNT11;PLAGL1;NRIP1;E2F3;APBB2;TBL1X;MEF2D;BRD4;MLXIP;WWOX;PCGF5;MBD2;FOXJ3;KLF4;GATAD2B;ACVR2A;KLF2;GATAD2A;HIPK2;ASXL1;KLF6;KANSL1;KANSL3;ASXL2;QRICH1;NAA15;CAMK1;SSBP2;LPIN2
Protein Localization (GO:0008104)	68/351	1.016E-08	2.6961E-06	2.331239367	42.9061136	ARF3;ARF4;CLTC;LYST;RAB22A;NIPBL;FGF7;AKAP11;SUMO1;SH3PXD2B;TMED2;VPS35;TMED7;ARL5A;TMED5;WLS;YWHAH;USP4;HEG1;COG3;STX7;VPS13A;AP3B1;FAM91A1;PARD3B;ARL4D;ARL4A;PARD3;UNC50;VCL;ARF6;STX12;COPA;STXBP3;SAR1B;VPS26A;EGFR;STX11;FBLN5;TM9SF3;RAB21;WNT11;SNX27;SCFD2;STX6;SNX9;SPTSSA;RAB6A;RAB6B;RIC1;USP9X;ATP8B1;SMURF1;LRBA;ERBIN;HOOK3;PEX13;ARCN1;EHD1;TM9SF1;NIN;NBEA;RAB18;RAB3GAP2;GOPC;LAMTOR3;RAB5A;NECTIN1
Endosomal Transport (GO:0016197)	42/180	3.3151E-08	8.0589E-06	2.935272965	50.55183088	WASHC4;STX12;DENND1A;CLTC;RHOBTB3;SNX12;VPS26A;CLN5;SNX4;SNX2;SNX27;TOM1;STX6;VPS54;VPS35;SNX9;ERC1;RAB6A;SNX7;CCDC93;EVI5;RAB6B;EPS15;SNX5;SPAG9;RIC1;DENND2A;CMTM6;LMTK2;PLEKHA3;EPS15L1;EHD1;RAB10;ACAP2;PIKFYVE;SNX18;EHD4;TMCC1;VAMP4;PICALM;ARF6;VAMP3
Positive Regulation Of Transmembrane Receptor Protein Serine/Threonine Kinase Signaling Pathway (GO:0090100)	28/95	3.4164E-08	8.0589E-06	4.016261974	69.04794713	ACVRL1;CDKN1C;BMPR2;TNXB;RBPJ;THBS1;SDCBP;EP300;JAK2;RNF111;CREBBP;SMAD4;TGFB2;RBPMS;BMP8A;SULF1;TGFBR1;ACVR2A;TGFBR2;HIPK2;NUP93;BMP4;ADAM17;TWSG1;ZEB2;DAB2;RNF165;BMPR1A
Proteasome-Mediated Ubiquitin-Dependent Protein Catabolic Process (GO:0043161)	62/319	3.979E-08	8.8919E-06	2.336358298	39.81074705	UBE2D3;HERPUD1;TRIM2;CDC27;UBXN7;FBXO9;FBXW11;FBXW7;AXIN1;UBE4B;RAD23B;PJA2;KCTD10;PELI1;CLOCK;FAM8A1;PRKN;CUL3;CUL2;SEL1L;CUL1;RHOBTB3;NEDD4L;UBR3;AMBRA1;UBE2J2;RNF4;UBE2J1;ECPAS;ZNRF1;COP1;HECTD1;RNF216;PSMB2;SPOP;DNAJB9;TBL1X;RFFL;UBQLN2;KLHDC2;AKIRIN2;SMURF2;CSNK1A1;SMURF1;FEM1B;FBXL17;WWP1;UBE2G1;BFAR;PML;UBE2W;ITCH;SPSB1;NPLOC4;APPBP2;APC;MDM2;TRIP12;FBXL5;UBE2K;FBXL7;NFE2L2
Regulation Of Focal Adhesion Assembly (GO:0051893)	20/54	4.5259E-08	9.6085E-06	5.63944049	95.36782877	ACVRL1;BCAS3;MACF1;LIMCH1;ROCK2;PTPRJ;EFNA5;ARHGAP6;THBS1;PTK2;ACTG1;SFRP1;FYN;RAC1;TEK;VCL;CLASP1;GPM6B;CLASP2;LIMS1

Peptidyl-Serine Phosphorylation (GO:0018105)	38/158	6.3015E-08	1.2741E-05	3.050589496	50.57843088	SMG1;CAMK2D;DYRK2;MAST4;CAB39;TNKS;ROCK2;MAST2;RPS6KA3;MAPK9;TBK1;AKT3;STK38;MAP3K20;TLK2;MAPK1;RICTOR;MARK3;CSNK1G3;MORC3;PRKCI;PDPK1;CSNK1A1;PRKCD;DYRK1A;LMTK2;PRKCA;TGFBR1;TGFBR2;HIPK2;ERN1;LATS1;PIKFYVE;RPS6KB1;WNK1;PKN2;CAMK1;SGK1
Peptidyl-Serine Modification (GO:0018209)	39/166	8.3334E-08	1.6084E-05	2.958734247	48.22856385	SMG1;CAMK2D;DYRK2;GALNT16;MAST4;CAB39;TNKS;ROCK2;MAST2;RPS6KA3;MAPK9;TBK1;AKT3;STK38;MAP3K20;TLK2;MAPK1;RICTOR;POGLUT3;MARK3;CSNK1G3;MORC3;PRKCI;PDPK1;CSNK1A1;GALNT1;PRKCD;DYRK1A;LMTK2;PRKCA;TGFBR1;TGFBR2;HIPK2;LATS1;RPS6KB1;WNK1;PKN2;CAMK1;SGK1
Positive Regulation Of Cell Differentiation (GO:0045597)	56/283	9.5064E-08	1.7492E-05	2.385395808	38.56879326	ACVRL1;MTCH2;BMPR2;CSF1;SYAP1;LAMC1;MEDAG;LOXL2;TMEM100;RPS6KA3;ZC3H12A;NRCAM;CD34;PBRM1;ZHX3;IGFBP3;TENT5A;NGF;TGFBR1;TGFBR2;SFRP1;GPRC5B;SFRP2;ZEB1;RAPGEF2;DDR2;CEBPB;TNXB;LAMA2;NID1;ZFP36L1;SDCBP;MAPK9;RAP1A;NOCT;WWTR1;XBP1;AKIRIN1;SMAD4;TGFB2;SMAD3;PHF10;RARRES2;SUCO;LAMB1;PTK2;NFKB1;ACVR2A;BMP4;NUDT21;DAB2;IL6;ASXL2;CAMK1;BMPR1A;FGFR1
Membrane Organization (GO:0061024)	39/167	9.8872E-08	1.7492E-05	2.935455764	47.34725203	GLTP;SAR1B;SNAP23;TOR1AIP2;WASL;PIK3C2A;ATP2C1;TOR1AIP1;EGFR;STX11;LMNB1;DPP4;SPAST;TMEM43;FCHO2;CHP1;SPTBN1;SH3GLB1;PRKCI;PDCD6IP;EXOC6B;STAM;NUP93;RABEP1;SYNJ1;NPC1;VAPA;TMEM33;REEP3;MYADM;AAK1;EXOC4;UBAP1;TMCC1;EXOC5;STAM2;PICALM;CHMP5;VAMP3
Positive Regulation Of Epithelial Cell Migration (GO:0010634)	27/95	1.3728E-07	2.3315E-05	3.81362625	60.26011642	BMPR2;ROCK2;ITGB3;FGF2;HIF1A;THBS1;RTN4;FGF7;ZC3H12A;ABL1;RAC1;CLASP1;CLASP2;BCAS3;TGFB2;ANXA3;IQSEC1;PRKCA;PTK2;RAB11A;FOXP1;BMP4;ADAM9;TEK;PLPP3;DOCK1;ARF6

Supplementary Table 13: Top 25 significantly enriched reactome terms for genes differentially expressed in Mfap5-/- mouse fibroblasts vs Mfap5+/+ mouse fibroblasts in NS (day 0 post-wounding)

Term	Overlap	P.value	Adjusted.P.value	Odds.Ratio	Combined.Score	Genes
Signal Transduction R-HSA-162582	410/2465	6.3662E-33	9.8612E-30	2.14216477	158.807912	NCKAP1;CYFIP1;TRIO;MAML2;WIPF1;MAML1;RBPJ;MYC;AKT3;VPS35;RNF111;WLS;MEF2A;PRKAB2;PRKCI;PRKCD;MRTFA;FRS2;PRKCA;DYNC1LI2;DAAM1;PARD3;PSME4;NUP98;MTMR1;NEDD4L;VPS26A;HIF1A;RHOT1;FLRT2;PDGFD;PIP5K1A;ABCA1;FZD1;CREBBP;JUND;JUP;FN1;BRAF;PHC3;CYLD;HNRNPF;AGO2;CDC42SE2;CDKN1A;RARG;ARPC1A;CLTC;ADM;SHB;ARHGAP5;ARHGAP6;GLI3;SMPD3;C1QTNF1;JAK2;JAK1;CNOT6L;ANXA1;RIPK2;RRAS2;EPS15L1;F3;APBB1IP;PLCB4;PXDN;RAPGEF1;RAPGEF2;GAS1;MGLL;VAMP3;ARF6;PHLPP2;CCL11;PHLPP1;ADCY2;FSTL1;TANK;EGFR;PSMB2;SPOP;RICTOR;CKB;MEF2D;FARP2;WWOX;CSNK1A1;KIDINS220;USP9X;WWP1;GATAD2B;GATAD2A;KTN1;TMEM59;ABI2;TAOK1;ABI1;FOSB;PKN2;GOPC;FILIP1;LAMTOR3;LGR4;CCNT2;CCNT1;FAM13B;NAB1;UBE2D3;RORA;IRS2;MSI2;IL1RAP;CXCL13;STK10;FGF7;SLK;SCMH1;GRB10;MAP2K1;TLE1;FBXW7;RALGAPA1;USP4;FST;SWAP70;USP2;FAM91A1;LEMD3;CLIP1;FKBP5;NSMAF;NOTCH2;KHDRBS1;CALCRL;TNKS;CUL3;RHOBTB3;CUL1;ITPR2;RASAL2;KLC1;SAV1;RAP1B;RAP1A;STK38;ABL1;ABL2;SRGAP2;RAB6A;SRGAP1;SKIL;MAP2K5;NFKB1;MOB1B;CNOT6;ARPC2;NBEA;RHEB;COPS2;MDM2;NF1;NF2;BMPR2;ITGB5;ITGB3;SNAP23;ECE1;CDC73;DUSP10;PRKG2;ITGAV;PRKG1;ACTR3;PLEKHG3;DST;NGF;PPM1A;PIK3CA;NUMB;PPP1R12B;DAGLB;SOS1;CRK;SOS2;BIRC3;HDAC4;HDAC2;GTF2A2;ROCK2;HDAC8;HDAC7;ZNRF3;CCL7;CCL2;FYN;E2F3;EIF4E;EIF4B;PCDH7;TXNL1;PTPN11;CDC42BPB;CDC42BPA;PML;VAPA;TRIM33;PTPN2;CPNE8;APP;AHCYL1;RPS6KA3;RPS6KA5;TMPO;SFRP1;SFRP2;TRIB1;ATP6V0D1;LPAR1;MYL12B;NLN;ATP6V1C1;SPTBN1;SCAI;WWTR1;YES1;INSR;MEMO1;STAM;GNG12;PTK2;ZWINT;STAG2;PDE3A;ID3;GRB2;RCOR1;ACVRL1;OTUD7B;PTPRJ;WASL;PCSK6;CHRDL1;PCSK5;AKAP12;AKAP13;TMED2;TMED5;NCOA2;GAB2;RHOC;KIF2A;RHOJ;ARHGEF3;RHOU;SGK1;DBN1;RHOQ;RAMP2;CAB39;GNAI3;XIAP;TRAK1;PPP2CA;PPP2CB;CTNNA1;TBL1X;EPS15;CD55;RANBP2;SUZ12;GIT2;RYK;ERBIN;RANBP9;VEGFD;MYO9A;MPP7;MIB1;ITCH;AXL;NEDD4;DLC1;OTULIN;TRIP10;SPPL2A;PLAT;NR3C1;ARHGAP35;IGF1R;ACTG1;DOCK10;SYDE2;DOCK11;ARHGAP42;BASP1;KIF5B;EP300;PDE8A;TMOD3;ADAM17;COL4A4;ELMO1;STAM2;VCL;ATP6V1A;DDX5;SEL1L;ARHGAP17;DTX2;PSEN1;LTBP1;RTN4;TOR1AIP1;ARHGAP12;ARHGAP20;GRK5;UBB;UBC;ATP6V1H;WNT2;MARK3;MYH10;SMAD1;TGFB2;SMAD4;PDHA1;POU2F1;SMAD3;SMURF2;SMURF1;ARHGAP29;MACO1;ARHGAP28;ESR1;ARHGAP24;SMAD7;DIAPH1;DIAPH2;IL6;FER;ARHGAP31;DNMBP;GNAQ;GNB4;FGFR1;BMPR1A;PICALM;DOCK4;TNFAIP3;ATP2A2;HSPB1;CXCL1;LAMC1;RND3;CHD1;LMNB1;HEBP1;SPRED1;EEF2K;PPP2R5E;DACT1;YWHAH;TGIF1;ARHGEF10;ARHGEF12;PDPK1;EED;MMP2;AXIN1;PLA2G4A;TGFBR1;TGFBR2;LATS1;ARHGAP10;RASA3;ALDH1A1;ATP6V1B2;COL6A6;DOCK1;CAMK2D;USP15;LAMA2;LAMA4;RAP1GDS1;MOSPD2;ADD3;THBS2;THBS1;UACA;RNF2;GNA13;WNT11;NRIP1;LRIG1;MAPK1;MAPK6;CLASP1;CLASP2;USP25;LAMB1;ACVR2A;SLC4A7;PDE10A;FMNL2;KANSL1;RPS6KB1;ABHD17B;ACKR3;TEK;ACKR1
Signaling By Rho GTPases, Miro GTPases And RHOBTB3 R-HSA-9716542	144/660	6.062E-22	4.695E-19	2.78752643	136.184149	CYFIP1;NCKAP1;CPNE8;TRIO;FAM13B;WIPF1;MSI2;ARHGAP35;ACTG1;DOCK10;SYDE2;STK10;DOCK11;ARHGAP42;SLK;BASP1;KIF5B;TMPO;RALGAPA1;SWAP70;TMOD3;PRKCD;MRTFA;FRS2;PRKCA;FAM91A1;LEMD3;DYNC1LI2;CLIP1;DAAM1;NUP98;STAM2;MTMR1;CUL3;RHOBTB3;ARHGAP17;RASAL2;KLC1;TOR1AIP1;MYL12B;ARHGAP12;ARHGAP20;RHOT1;STK38;ABL1;ABL2;SRGAP2;SRGAP1;MYH10;SCAI;SPTBN1;JUP;ARHGAP29;ARHGAP28;MACO1;STAM;PTK2;ZWINT;ARHGAP24;DIAPH1;ARHGAP31;DIAPH2;DNMBP;ARPC2;COPS2;GRB2;NF2;CDC42SE2;PICALM;DOCK4;ARPC1A;CLTC;SNAP23;WASL;ARHGAP5;ARHGAP6;RND3;LMNB1;AKAP12;AKAP13;PPP2R5E;YWHAH;ACTR3;PLEKHG3;NCOA2;ARHGEF10;ARHGEF12;DST;PDPK1;RRAS2;RHOC;ARHGAP10;PIK3CA;KIF2A;RHOJ;RAPGEF1;ARHGEF3;RHOU;PPP1R12B;SOS1;SOS2;DOCK1;DBN1;RHOQ;VAMP3;ROCK2;RAP1GDS1;MOSPD2;TRAK1;ADD3;UACA;PPP2CA;GNA13;PPP2CB;CTNNA1;MAPK1;CKB;CLASP1;CLASP2;FARP2;RANBP2;GIT2;KIDINS220;USP9X;PCDH7;TXNL1;ERBIN;CDC42BPB;CDC42BPA;MYO9A;MPP7;KTN1;SLC4A7;TMEM59;FMNL2;ABI2;KANSL1;TAOK1;DLC1;ABI1;TRIP10;FILIP1;GOPC;PKN2

Immune System R-HSA-168256	311/1943	1.0238E-21	5.2862E-19	1.96951737	95.188272	NCKAP1;CYFIP1;APP;AHCYL1;WIPF1;CBLB;IFI35;DCAF1;RPS6KA3;RNF115;TBK1;RPS6KA5;ANPEP;MYC;AKT3;CDC27;AP1S2;CPNE3;RNF111;FBXO9;MEF2A;IL15RA;GBP7;IL1R1;PRKCD;PRKCA;SCAMP1;NUP93;DYNC1LI2;HECW2;PSME4;SERPING1;NUP98;ATP6V0D1;SAR1B;DHX9;MAOA;MGST1;NEDD4L;BCL10;UBE2J2;GNS;HIF1A;UBE2J1;RNF216;RNF217;ATP6V1C1;ARSB;N4BP1;VAT1;CREBBP;YES1;JUP;FN1;LNPEP;MEX3C;PTK2;CYLD;HNRNPF;TOLLIP;REL;GRB2;FBXL5;CDK13;FBXL7;LGMN;PYGB;CDKN1A;HEXB;ARPC1A;CLTC;GDI2;PTPRJ;PYGL;WASL;IFIH1;EEA1;CTSO;CTSL;CTSK;TRIM2;TIMP2;ARIH2;CTSF;JAK2;JAK1;ANXA1;TNFRSF12A;RIPK2;IFNGR1;IFNGR2;ANO6;GAB2;AMPD3;OSMR;ENAH;ZEB1;KIF2A;GAN;RAPGEF1;PELI1;RHOU;PLEKHO2;CD47;TLR4;PAFAH1B2;CD44;VAMP3;PRKN;ASAH1;CCL11;CAB39;PELI2;PTGS2;TANK;RELB;PPP2CA;PPP2CB;PSMB2;RICTOR;FLNB;XDH;CD55;RANBP2;MAP3K1;IL10RB;HUWE1;KLHL2;WWP1;UBE2G1;ERP44;ITCH;SPSB1;ABI2;QPCT;CAPZA1;NEDD4;DLC1;CAPZA2;ABI1;PKN2;TRIP12;ACTR10;LAMTOR3;CSF1;PROS1;UBE2D3;RNF19B;UBE3A;RORA;IRS2;IL1RAP;PVR;ACTG1;NPEPPS;STK10;PLAU;KIF5B;RNF19A;LAMP2;TOM1;GRB10;EP300;SIRPA;MAP3K8;KPNA4;KPNA3;IL13RA1;KPNA1;KCMF1;MAP2K4;MAP2K1;FBXW7;FBXW11;UBE2E3;UBE2E1;UBE2E2;ATP11B;ATP11A;FBXO11;MID1;PIAS1;EEF1A1;ACLY;ADAM17;STIM1;UBE2V2;ELMO1;VCL;ATP6V1A;UBA6;COPB1;CUL3;CUL2;UBR4;CUL1;ITPR2;KLHL13;PSEN1;KLC1;RAP1B;SOCS2;SDCBP;RAP1A;UBB;BTBD1;UBC;ABL1;ABL2;ATP6V1H;RAB6A;MCL1;SOCS5;UBE2F;UBE2H;POU2F1;SMAD3;SMURF2;SMURF1;GGH;NUP153;DNAJC13;HOOK3;FBXO30;IGF2R;NFKB1;SMAD7;UBE2W;DIAPH1;RCAN1;IL6;ARPC2;UBE2N;UBA3;TPP2;NF2;UBE2K;EIF4G3;EDA;ITGB5;TNFAIP6;SNAP23;TNFAIP3;CXCL1;LMNB1;GHR;SEC61A2;RO60;SUMO1;UBE2Q1;AP1G1;PPP2R5E;CASP4;ITGAV;LBP;ATP7A;PRKG1;ACTR3;APAF1;PDPK1;MMP2;PLAUR;IRAK3;TNFRSF1B;PJA2;TAPBP;DNAJC3;PPM1B;PIK3CA;UBE2R2;DNAJC5;NUP50;IRF2;ATP6V1B2;SOS1;CRK;SOS2;DOCK1;BIRC3;COLEC12;USP14;CAMK2D;CEBPD;FGL2;MOSPD2;PRCP;RCHY1;RNF4;SAMHD1;ZNRF1;MAPK9;IL1RL2;HECTD1;HECTD2;MAPK1;CCL2;FYN;GBP2;EIF4E;SEC24B;TMEM30A;SEC24A;CMTM6;PTPN11;PML;RAB10;PIKFYVE;VAPA;RAB18;PTX3;PTPN2
Signaling By Rho GTPases R-HSA-194315	140/644	3.324E-21	1.2872E-18	2.7702192	130.62448	CYFIP1;NCKAP1;CPNE8;TRIO;FAM13B;WIPF1;MSI2;ARHGAP35;ACTG1;DOCK10;SYDE2;STK10;DOCK11;ARHGAP42;SLK;BASP1;KIF5B;TMPO;RALGAPA1;SWAP70;TMOD3;PRKCD;MRTFA;FRS2;PRKCA;FAM91A1;LEMD3;DYNC1LI2;CLIP1;DAAM1;NUP98;STAM2;MTMR1;CUL3;ARHGAP17;RASAL2;KLC1;TOR1AIP1;MYL12B;ARHGAP12;ARHGAP20;STK38;ABL1;ABL2;SRGAP2;SRGAP1;MYH10;SCAI;SPTBN1;JUP;ARHGAP29;ARHGAP28;MACO1;STAM;PTK2;ZWINT;ARHGAP24;DIAPH1;ARHGAP31;DIAPH2;DNMBP;ARPC2;COPS2;GRB2;NF2;CDC42SE2;PICALM;DOCK4;ARPC1A;CLTC;SNAP23;WASL;ARHGAP5;ARHGAP6;RND3;LMNB1;AKAP12;AKAP13;PPP2R5E;YWHAH;ACTR3;PLEKHG3;NCOA2;ARHGEF10;ARHGEF12;DST;PDPK1;RRAS2;RHOC;ARHGAP10;PIK3CA;KIF2A;RHOJ;RAPGEF1;ARHGEF3;RHOU;PPP1R12B;SOS1;SOS2;DOCK1;DBN1;RHOQ;VAMP3;ROCK2;MOSPD2;ADD3;UACA;PPP2CA;GNA13;PPP2CB;CTNNA1;MAPK1;CKB;CLASP1;CLASP2;FARP2;RANBP2;GIT2;KIDINS220;USP9X;PCDH7;TXNL1;ERBIN;CDC42BPB;CDC42BPA;MYO9A;MPP7;KTN1;SLC4A7;TMEM59;FMNL2;ABI2;KANSL1;TAOK1;DLC1;ABI1;TRIP10;FILIP1;GOPC;PKN2
RHO GTPase Cycle R-HSA-9012999	107/441	3.922E-20	1.215E-17	3.16651172	141.495909	CYFIP1;NCKAP1;CPNE8;TRIO;FAM13B;WIPF1;MSI2;ARHGAP35;ACTG1;DOCK10;SYDE2;STK10;DOCK11;ARHGAP42;SLK;BASP1;TMPO;RALGAPA1;SWAP70;TMOD3;FRS2;FAM91A1;LEMD3;DAAM1;STAM2;MTMR1;CUL3;ARHGAP17;RASAL2;TOR1AIP1;ARHGAP12;ARHGAP20;STK38;ABL2;SRGAP2;SRGAP1;SPTBN1;JUP;ARHGAP29;ARHGAP28;MACO1;STAM;ARHGAP24;DIAPH1;ARHGAP31;DIAPH2;DNMBP;COPS2;GRB2;CDC42SE2;PICALM;DOCK4;CLTC;SNAP23;WASL;ARHGAP5;ARHGAP6;RND3;LMNB1;AKAP12;AKAP13;PLEKHG3;ARHGEF10;ARHGEF12;DST;RRAS2;RHOC;ARHGAP10;PIK3CA;RHOJ;RAPGEF1;ARHGEF3;RHOU;SOS1;SOS2;DOCK1;DBN1;RHOQ;VAMP3;ROCK2;MOSPD2;ADD3;UACA;GNA13;CKB;FARP2;GIT2;KIDINS220;USP9X;PCDH7;TXNL1;ERBIN;CDC42BPB;CDC42BPA;MYO9A;MPP7;KTN1;SLC4A7;TMEM59;FMNL2;ABI2;DLC1;ABI1;TRIP10;FILIP1;GOPC;PKN2
Signaling By Receptor Tyrosine Kinases R-HSA-9006934	112/496	1.8356E-18	4.739E-16	2.8828125	117.731598	CYFIP1;NCKAP1;AHCYL1;NAB1;IRS2;PLAT;IGF1R;RPS6KA3;FGF7;RPS6KA5;MYC;AKT3;GRB10;EP300;MEF2A;MAP2K1;PRKCD;FRS2;PRKCA;ADAM17;COL4A4;ELMO1;ATP6V0D1;TRIB1;STAM2;ATP6V1A;ITPR2;PSEN1;HIF1A;RAP1B;RAP1A;FLRT2;UBB;PDGFD;UBC;ATP6V1H;MAP2K5;ATP6V1C1;JUND;YES1;JUP;INSR;BRAF;MEMO1;STAM;ESR1;PTK2;DIAPH1;FER;HNRNPF;COPS2;ID3;GRB2;FGFR1;ITGB3;CLTC;HSPB1;PTPRJ;LAMC1;SHB;PCSK6;PCSK5;SPRED1;ITGAV;JAK2;PDPK1;EPS15L1;GAB2;NGF;F3;PIK3CA;PXDN;RAPGEF1;ATP6V1B2;COL6A6;SOS1;SGK1;CRK;DOCK1;ARF6;HDAC2;LAMA2;ROCK2;LAMA4;THBS2;THBS1;EGFR;PPP2CA;PPP2CB;CTNNA1;LRIG1;MAPK1;RICTOR;FYN;MEF2D;EPS15;WWOX;KIDINS220;ERBIN;WWP1;RANBP9;PTPN11;VEGFD;LAMB1;ITCH;ABI2;AXL;NEDD4;ABI1;FOSB;PKN2;PTPN2
Post-translational Protein Modification R-HSA-597592	225/1383	1.653E-16	3.6578E-14	1.96013233	71.2288117	APP;DCAF5;DCAF6;TFG;MYC;DAG1;THSD7A;FBXO9;NSMCE2;COG3;MRTFA;NUP93;DYNC1LI2;PSME4;MXRA8;VDAC2;NUP98;DCUN1D5;SAR1B;DCUN1D3;BCL10;ADAMTS12;UBE2J2;HIF1A;RHOT1;ST3GAL1;ARSB;ST3GAL2;SPTBN1;B3GALNT2;EDEM3;NUS1;CREBBP;EDEM2;FN1;STAM;PHC3;PUM2;CYLD;NPLOC4;STAG2;FBXL5;FBXL7;ARF3;ARF4;TOP2B;OTUD7B;MIA3;IDE;CHRDL1;PRSS23;RAB22A;ADAMTSL1;IFIH1;BABAM2;ADAMTSL4;UIMC1;GNPNAT1;TMED2;UBXN7;TMED7;RAB8B;NCOA2;RIPK2;LRRC49;TUBB2B;GAN;WAC;PRKN;WDR20;FSTL1;TANK;PSMB2;STAMBPL1;CD55;RANBP2;SUZ12;MBD5;USP9X;MBD1;C1GALT1;KLHL2;UBE2G1;PEX13;RAB11A;ARCN1;HIPK2;KTN1;SPSB1;CAPZA1;OTULIN;CAPZA2;ACTR10;CSF1;TUSC3;PROS1;UBE2D3;RORA;NR3C1;TOMM20;TUBB6;SCMH1;EP300;LARGE1;IGFBP5;FBXW7;FBXW11;USP4;UBE2E3;IGFBP3;USP2;UBE2E1;MITF;FBXO11;PIAS2;PIAS1;TPST2;EEF1A1;UBE2V2;STAM2;DDX5;COPA;INO80D;UBA6;TNKS;CUL3;COPB1;SEL1L;CUL2;CUL1;UAP1;KLHL13;LTBP1;SOCS2;VCPIP1;FUT8;BTBD1;UBB;MAN2A1;UBC;NAE1;SUDS3;RAB6A;RAB6B;SOCS5;UBE2F;ANKRD28;SMAD1;UBE2H;SMAD4;SMAD3;SMURF2;GFPT2;NUP153;ESR1;FBXO30;SMAD7;BMP4;UBE2W;IL6;CNIH1;COPS2;SP3;MDM2;UBA3;UBE2N;UBE2K;RAB5A;NFE2L2;FBN1;THRB;USP32;TNFAIP3;LAMC1;CDC73;ADAMTS5;PPP6C;SUMO1;MAN1A2;ADAMTS1;SUMO2;PGM3;USP47;AXIN1;PLAUR;SENP2;RAD23B;TGFBR1;TGFBR2;DNAJC3;VCAN;NSMCE4A;UBE2R2;NUP50;BIRC3;B4GALT5;HDAC4;USP14;HDAC2;USP15;UHRF2;TULP4;THBS2;THBS1;RNF2;HDAC7;RAB21;GMDS;NRIP1;DCAF10;OPCML;USP24;USP25;SEC24B;SEC24A;FEM1B;GALNT1;LAMB1;PML;ASXL1;RAB10;RAB12;ASXL2;RAB18;RECK

Membrane Trafficking R-HSA-199991	116/599	6.2633E-14	1.2127E-11	2.36579265	71.923592	SCARB2;APP;SCOC;DENND5B;RABGEF1;TFG;KIF5B;AKT3;AP1S2;KIF21A;SBF2;AGFG1;PRKAB2;RALGAPA2;COG3;DYNC1LI2;STAM2;COPA;STXBP3;SAR1B;COPB1;RHOBTB3;KLC1;GNS;SNX2;UBB;MAN2A1;UBC;PACSIN2;SNX9;RAB6A;RAB6B;SNX5;SPTBN1;ANKRD28;DENND4A;DENND4C;LNPEP;TRAPPC8;STAM;PUM1;IGF2R;DAB2;CNIH1;ARPC2;SYNJ1;COPS2;GRB2;RAB5A;PICALM;ARF3;ARF4;DENND1A;ARPC1A;CLTC;SNAP23;GDI2;MIA3;STON2;WASL;PIK3C2A;CLINT1;BICD2;PPP6C;AP1G1;MAN1A2;KIF13B;TMED2;KIF1B;AP3S1;TMED7;RAB8B;YWHAH;ACTR3;DENND2C;DENND2A;PLA2G4A;AP3B1;EPS15L1;RABEP1;KIF2A;EXOC4;EXOC5;VAMP4;PAFAH1B2;RHOQ;ARF6;VAMP3;CHMP5;EGFR;RAB21;CYTH3;FCHO2;STX6;VPS54;EPS15;CD55;CYTH1;UBQLN2;SEC24B;RIC1;SEC24A;GALNT1;DTNBP1;RAB11A;ARCN1;RAB10;SNX18;RAB12;CAPZA1;CAPZA2;RAB18;TRIP10;UBAP1;RAB3GAP2;ACTR10
Vesicle-mediated Transport R-HSA-5653656	119/637	3.9529E-13	6.8034E-11	2.26229086	64.6091016	SCARB2;APP;SCOC;DENND5B;RABGEF1;TFG;KIF5B;AKT3;AP1S2;KIF21A;SBF2;AGFG1;PRKAB2;SCARA5;RALGAPA2;COG3;DYNC1LI2;STAM2;COPA;STXBP3;SAR1B;COPB1;RHOBTB3;KLC1;GNS;SNX2;UBB;MAN2A1;UBC;PACSIN2;SNX9;RAB6A;RAB6B;SNX5;SPTBN1;ANKRD28;DENND4A;DENND4C;LNPEP;TRAPPC8;STAM;PUM1;IGF2R;DAB2;CNIH1;ARPC2;SYNJ1;COPS2;GRB2;RAB5A;PICALM;ARF3;ARF4;DENND1A;ARPC1A;CLTC;SNAP23;GDI2;MIA3;STON2;WASL;PIK3C2A;CLINT1;BICD2;PPP6C;AP1G1;MAN1A2;KIF13B;TMED2;KIF1B;AP3S1;TMED7;RAB8B;YWHAH;ACTR3;DENND2C;DENND2A;PLA2G4A;AP3B1;EPS15L1;RABEP1;KIF2A;EXOC4;EXOC5;VAMP4;PAFAH1B2;RHOQ;ARF6;VAMP3;CHMP5;COLEC12;EGFR;RAB21;CYTH3;FCHO2;STX6;VPS54;EPS15;CD55;CYTH1;UBQLN2;SEC24B;RIC1;SEC24A;GALNT1;DTNBP1;RAB11A;ARCN1;RAB10;SNX18;RAB12;CAPZA1;DLC1;CAPZA2;RAB18;TRIP10;UBAP1;RAB3GAP2;ACTR10
Disease R-HSA-1643685	251/1736	2.8729E-12	4.4501E-10	1.69851815	45.1393124	RB1;NCKAP1;CYFIP1;APP;AHCYL1;MAML2;WIPF1;CCNH;MAML1;RBPJ;ANTXR2;SLC4A4;ANTXR1;TBK1;TFG;MPRIP;MYC;AKT3;CDC27;AP1S2;DAG1;CYP1B1;THSD7A;SLC12A6;IL1R1;OMD;SLC11A2;FRS2;PRKCA;SND1;NUP93;EML4;DYNC1LI2;PSME4;RBP1;SERPING1;NUP98;SDC4;SAR1B;MAOA;NEDD4L;ADAMTS12;GNS;LARP1;ST3GAL1;ST3GAL2;SPTBN1;ARSB;ABCA1;NUS1;CREBBP;YES1;EDEM2;FN1;BRAF;STAM;GNG12;PTK2;EXT1;POLA1;PPFIBP1;NMT2;NOX4;GRB2;RCOR1;CDKN1C;CDKN1A;ELL;GBE1;HEXB;ARPC1A;CLTC;ADM;WASL;MECP2;ADAMTSL1;IFIH1;C1QTNF1;ADAMTSL4;CTSL;JAK2;JAK1;PBRM1;RIPK2;IFNGR1;IFNGR2;AP3B1;GAB2;PARP9;PARP8;APBB1IP;RAD51B;PACS1;TLR4;RAMP2;ZDHHC20;GNAI3;ADCY2;TRAK1;TANK;EGFR;PAPSS2;PPP2CA;PPP2CB;PSMB2;RICTOR;TBL1X;MAP4K5;EPS15;GPC6;BRD4;RANBP2;SUZ12;SLC16A1;CSNK1A1;C1GALT1;ERBIN;GATAD2B;GATAD2A;MIB1;ABI2;ABI1;UBAP1;PKN2;CCNT2;CCNT1;TUSC3;GTF2B;IRS2;ARID4B;NR3C1;ACTG1;IPO5;FGF7;KIF5B;CAPN2;EP300;KPNA4;LARGE1;KPNA3;KPNA1;MAP2K4;MAP2K1;FBXW7;UBE2E1;TPST2;ADAM17;SLCO2A1;SLC9A9;ELMO1;DPEP1;STAM2;VCL;NOTCH2;DDX5;CALCRL;TNKS;CUL3;SEL1L;CUL1;ITPR2;PSEN1;KLC1;GTF2E2;RAP1B;FUT8;RAP1A;UBB;MAN2A1;G3BP1;UBC;ABL1;ATP6V1H;SUDS3;MARK3;UVRAG;SMAD4;CPSF6;SMAD3;NUP153;ESR1;NFKB1;GCLC;IL6;ARPC2;OGN;GNB4;UBE2N;MDM2;NF1;GCLM;RAB5A;FGFR1;NFE2L2;ITGB3;TENT4A;LMNB1;ADAMTS5;SPRED1;DUSP10;SUMO1;ADAMTS1;AP1G1;PPP2R5E;YWHAH;ACTR3;PDCD6IP;PDPK1;EED;AXIN1;TGFBR1;ETV6;TGFBR2;DNAJC3;PPM1B;LMBRD1;VCAN;PIK3CA;NUP50;SOS1;CRK;DOCK1;KDM7A;CHMP5;HDAC4;CEBPB;HDAC2;GTF2A2;CAMK2D;CEBPD;ROCK2;PRELP;HDAC8;THBS2;THBS1;HDAC7;FIP1L1;SAP30L;RBBP8;LRIG1;MAPK1;E2F3;FYN;SEC24B;RNGTT;SEC24A;GALNT1;TXNRD1;PTPN11;PML;P2RX4
Signaling By TGFB Family Members R-HSA-9006936	38/119	8.9287E-12	1.2301E-09	4.52919694	115.230686	ACVRL1;USP15;CCNT2;BMPR2;CCNT1;ITGB5;ITGB3;UBE2D3;NEDD4L;LTBP1;CHRDL1;FSTL1;UBB;MYC;UBC;EP300;MAPK1;ITGAV;SKIL;RNF111;TGIF1;SMAD1;WWTR1;SMAD4;TGFB2;SMAD3;SMURF2;SMURF1;USP9X;FST;TGFBR1;ACVR2A;SMAD7;TGFBR2;PPM1A;PARD3;TRIM33;BMPR1A
Diseases Of Signal Transduction By Growth Factor Receptors And Second Messengers R-HSA-5663202	86/424	9.5297E-12	1.2301E-09	2.48531776	63.0689416	CDKN1A;MAML2;MAML1;ITGB3;CLTC;IRS2;RBPJ;TENT4A;MECP2;FGF7;SPRED1;DUSP10;MPRIP;TFG;PPP2R5E;MYC;KIF5B;AKT3;EP300;JAK2;MAP2K1;FBXW7;PDPK1;AXIN1;FRS2;AP3B1;GAB2;SND1;TGFBR1;ETV6;TGFBR2;EML4;APBB1IP;PPM1B;ADAM17;PIK3CA;PSME4;SOS1;VCL;KDM7A;HDAC4;CEBPB;HDAC2;CAMK2D;TNKS;SEL1L;CUL1;PSEN1;KLC1;HDAC8;TRAK1;EGFR;HDAC7;RAP1B;PPP2CA;PPP2CB;RAP1A;PSMB2;FIP1L1;UBB;UBC;MAPK1;RICTOR;FYN;TBL1X;MARK3;SPTBN1;RANBP2;CREBBP;SMAD4;YES1;CPSF6;SMAD3;CSNK1A1;FN1;ERBIN;BRAF;PTPN11;ESR1;MIB1;PPFIBP1;MDM2;NF1;NOX4;GRB2;FGFR1
Class I MHC Mediated Antigen Processing And Presentation R-HSA-983169	78/378	3.6976E-11	4.4059E-09	2.53393209	60.8669438	ITGB5;UBE2D3;SNAP23;RNF19B;CBLB;UBE3A;DCAF1;SEC61A2;NPEPPS;RNF115;UBE2Q1;CTSL;RNF19A;CDC27;ARIH2;ITGAV;RNF111;FBXO9;FBXW11;FBXW7;UBE2E3;UBE2E1;UBE2E2;FBXO11;PJA2;TAPBP;UBE2R2;GAN;HECW2;PSME4;UBE2V2;TLR4;VAMP3;PRKN;SAR1B;UBA6;CUL3;CUL2;CUL1;UBR4;NEDD4L;KLHL13;UBE2J2;RCHY1;RNF4;UBE2J1;ZNRF1;HECTD1;PSMB2;BTBD1;UBB;HECTD2;RNF217;UBC;UBE2F;UBE2H;SEC24B;SEC24A;SMURF2;SMURF1;HUWE1;KLHL2;WWP1;LNPEP;UBE2G1;MEX3C;FBXO30;UBE2W;ITCH;SPSB1;NEDD4;UBA3;UBE2N;TPP2;TRIP12;FBXL5;UBE2K;FBXL7
Innate Immune System R-HSA-168249	163/1035	5.0152E-11	5.549E-09	1.84929572	43.8578158	CYFIP1;NCKAP1;APP;AHCYL1;WIPF1;PROS1;UBE2D3;ACTG1;STK10;RPS6KA3;TBK1;RPS6KA5;PLAU;ANPEP;LAMP2;TOM1;EP300;SIRPA;MAP3K8;CPNE3;MEF2A;KCMF1;MAP2K4;MAP2K1;FBXW11;PRKCD;ATP11B;PRKCA;ATP11A;SCAMP1;EEF1A1;ACLY;PSME4;ELMO1;SERPING1;ATP6V0D1;VCL;ATP6V1A;DHX9;COPB1;CUL1;UBR4;MGST1;ITPR2;PSEN1;BCL10;GNS;RAP1B;SDCBP;RAP1A;RNF216;UBB;UBC;ABL1;ATP6V1H;RAB6A;ARSB;ATP6V1C1;N4BP1;VAT1;CREBBP;YES1;JUP;GGH;DNAJC13;HOOK3;NFKB1;PTK2;IGF2R;CYLD;RCAN1;DIAPH1;ARPC2;TOLLIP;UBA3;UBE2N;GRB2;NF2;CDK13;UBE2K;LGMN;PYGB;TNFAIP6;HEXB;SNAP23;ARPC1A;GDI2;TNFAIP3;PTPRJ;CXCL1;WASL;PYGL;EEA1;IFIH1;RO60;CTSL;CTSK;CASP4;TIMP2;ITGAV;LBP;ATP7A;ACTR3;APAF1;RIPK2;PDPK1;PLAUR;ANO6;IRAK3;GAB2;AMPD3;TNFRSF1B;DNAJC3;PIK3CA;DNAJC5;PELI1;ATP6V1B2;CD47;PLEKHO2;SOS1;CRK;TLR4;DOCK1;PAFAH1B2;CD44;BIRC3;USP14;ASAH1;CAB39;PELI2;FGL2;MOSPD2;PRCP;TANK;RELB;PPP2CA;PPP2CB;MAPK9;PSMB2;MAPK1;FYN;CD55;MAP3K1;TMEM30A;CMTM6;HUWE1;PTPN11;RAB10;ERP44;ITCH;PIKFYVE;ABI2;VAPA;CAPZA1;DLC1;QPCT;ABI1;CAPZA2;RAB18;PKN2;PTX3;ACTR10;LAMTOR3

Cytokine Signaling In Immune System R-HSA-1280215	121/702	6.4359E-11	6.6461E-09	2.0458229	48.0084048	APP;CSF1;UBE2D3;IRS2;RORA;IL1RAP;IFI35;RPS6KA3;TBK1;RPS6KA5;MYC;AKT3;GRB10;KPNA4;MAP3K8;KPNA3;IL13RA1;KPNA1;MEF2A;MAP2K4;IL15RA;MAP2K1;GBP7;IL1R1;FBXW11;PRKCD;UBE2E1;PRKCA;MID1;PIAS1;NUP93;ADAM17;PSME4;NUP98;MAOA;CUL1;HIF1A;RAP1B;SOCS2;UBB;UBC;ABL2;N4BP1;MCL1;SOCS5;YES1;POU2F1;SMAD3;FN1;NUP153;NFKB1;SMAD7;IL6;HNRNPF;TOLLIP;UBA3;UBE2N;GRB2;EIF4G3;CDKN1A;EDA;PTPRJ;CXCL1;LMNB1;GHR;SUMO1;TRIM2;LBP;JAK2;JAK1;ANXA1;TNFRSF12A;IFNGR1;RIPK2;IFNGR2;MMP2;IRAK3;GAB2;TNFRSF1B;OSMR;PPM1B;ZEB1;PIK3CA;NUP50;IRF2;PELI1;RAPGEF1;RHOU;SOS1;CRK;SOS2;CD44;BIRC3;USP14;CAMK2D;CCL11;CEBPD;PELI2;PTGS2;SAMHD1;TANK;RELB;PPP2CA;PPP2CB;MAPK9;IL1RL2;PSMB2;CCL2;MAPK1;FLNB;FYN;GBP2;EIF4E;RANBP2;IL10RB;PTPN11;PML;CAPZA1;NEDD4;PKN2;PTPN2
Antigen Processing: Ubiquitination And Proteasome Degradation R-HSA-983168	67/307	7.122E-11	6.895E-09	2.71355471	63.4028634	UBE2D3;RNF19B;CBLB;UBE3A;DCAF1;NPEPPS;RNF115;UBE2Q1;RNF19A;CDC27;ARIH2;RNF111;FBXO9;FBXW11;FBXW7;UBE2E3;UBE2E1;UBE2E2;FBXO11;PJA2;UBE2R2;GAN;HECW2;PSME4;UBE2V2;PRKN;UBA6;CUL3;CUL2;CUL1;UBR4;NEDD4L;KLHL13;UBE2J2;RCHY1;RNF4;UBE2J1;ZNRF1;HECTD1;PSMB2;BTBD1;UBB;HECTD2;RNF217;UBC;UBE2F;UBE2H;SMURF2;SMURF1;HUWE1;KLHL2;WWP1;LNPEP;UBE2G1;MEX3C;FBXO30;UBE2W;ITCH;SPSB1;NEDD4;UBA3;UBE2N;TPP2;TRIP12;FBXL5;UBE2K;FBXL7
Adaptive Immune System R-HSA-1280218	122/733	5.1791E-10	4.7191E-08	1.95919001	41.8898816	AHCYL1;UBE2D3;RNF19B;CBLB;UBE3A;PVR;DCAF1;NPEPPS;RNF115;RNF19A;KIF5B;AKT3;CDC27;AP1S2;MAP3K8;RNF111;FBXO9;FBXW7;FBXW11;UBE2E3;UBE2E1;UBE2E2;PRKCA;FBXO11;DYNC1LI2;STIM1;HECW2;PSME4;UBE2V2;SAR1B;UBA6;CUL3;CUL2;CUL1;UBR4;NEDD4L;ITPR2;BCL10;KLHL13;KLC1;UBE2J2;UBE2J1;RAP1B;RAP1A;BTBD1;UBB;RNF217;UBC;UBE2F;UBE2H;YES1;SMURF2;SMURF1;LNPEP;MEX3C;FBXO30;NFKB1;UBE2W;REL;UBA3;UBE2N;TPP2;GRB2;FBXL5;UBE2K;FBXL7;LGMN;ITGB5;CLTC;SNAP23;PTPRJ;SEC61A2;CTSO;UBE2Q1;AP1G1;CTSL;PPP2R5E;CTSK;ARIH2;ITGAV;CTSF;PRKG1;RIPK2;PDPK1;PJA2;TAPBP;ENAH;PIK3CA;KIF2A;UBE2R2;GAN;SOS1;TLR4;VAMP3;PRKN;COLEC12;RCHY1;RNF4;PPP2CA;ZNRF1;PPP2CB;HECTD1;PSMB2;HECTD2;RICTOR;FYN;XDH;SEC24B;SEC24A;HUWE1;WWP1;KLHL2;PTPN11;UBE2G1;SPSB1;ITCH;CAPZA1;NEDD4;CAPZA2;PKN2;TRIP12;ACTR10
Signaling By TGF-beta Receptor Complex R-HSA-170834	30/91	5.6742E-10	4.883E-08	4.73301608	100.765522	USP15;CCNT2;CCNT1;ITGB5;ITGB3;UBE2D3;NEDD4L;LTBP1;UBB;MYC;UBC;EP300;MAPK1;ITGAV;SKIL;RNF111;TGIF1;WWTR1;SMAD4;TGFB2;SMAD3;SMURF2;SMURF1;USP9X;TGFBR1;TGFBR2;SMAD7;PPM1A;PARD3;TRIM33
RAC1 GTPase Cycle R-HSA-9013149	45/178	7.2648E-10	5.9228E-08	3.26934199	68.7961237	CYFIP1;NCKAP1;DOCK4;TRIO;FAM13B;WIPF1;SNAP23;ARHGAP17;WASL;ARHGAP5;ARHGAP35;ARHGAP12;DOCK10;SYDE2;GNA13;DOCK11;ARHGAP42;ARHGAP20;ABL2;SRGAP2;SRGAP1;TMPO;PLEKHG3;FARP2;ARHGEF10;GIT2;SWAP70;ERBIN;ARHGAP29;LEMD3;CDC42BPA;ARHGAP24;MPP7;KTN1;ARHGAP10;ARHGAP31;PIK3CA;ABI2;DLC1;ABI1;PKN2;SOS1;DOCK1;SOS2;VAMP3
Downregulation Of SMAD2/3:SMAD4 Transcriptional Activity R-HSA-2173795	16/31	3.2055E-09	2.4827E-07	10.2152542	199.793945	TGIF1;WWTR1;SMAD4;SMAD3;SMURF2;USP9X;UBE2D3;NEDD4L;SMAD7;PPM1A;UBB;UBC;MAPK1;SKIL;RNF111;TRIM33
Metabolism Of Proteins R-HSA-392499	254/1890	3.5971E-09	2.6533E-07	1.54880344	30.1135875	APP;DCAF5;DCAF6;PCMT1;TFG;ANPEP;MYC;DAG1;THSD7A;FBXO9;COG3;NSMCE2;MRTFA;NUP93;DYNC1LI2;PPA1;PSME4;MXRA8;VDAC2;NUP98;DCUN1D5;SAR1B;DCUN1D3;BCL10;ADAMTS12;UBE2J2;HIF1A;RHOT1;TSPAN5;ST3GAL1;ST3GAL2;SPTBN1;ARSB;B3GALNT2;EDEM3;NUS1;CREBBP;ACE;EDEM2;FN1;STAM;GNG12;PHC3;PUM2;CYLD;NPLOC4;STAG2;FBXL5;FBXL7;ITM2B;ARF3;TOP2B;ARF4;OTUD7B;MIA3;IDE;CHRDL1;PRSS23;RAB22A;FARS2;ADAMTSL1;IFIH1;BABAM2;C1QTNF1;ADAMTSL4;UIMC1;TMED2;UBXN7;GNPNAT1;TMED7;RAB8B;NCOA2;RIPK2;LRRC49;SSR3;MRPL48;TUBB2B;GAN;EXOC4;WAC;EXOC5;PRKN;WDR20;FSTL1;TANK;PSMB2;STAMBPL1;CD55;CCT4;RANBP2;SUZ12;MBD5;USP9X;MBD1;C1GALT1;KLHL2;UBE2G1;PEX13;RAB11A;ARCN1;HIPK2;KTN1;SPSB1;CAPZA1;OTULIN;CAPZA2;ACTR10;CSF1;TUSC3;PROS1;UBE2D3;RORA;NR3C1;TOMM20;TUBB6;KIF5B;SCMH1;EP300;LARGE1;IGFBP5;FBXW7;FBXW11;USP4;UBE2E3;IGFBP3;USP2;UBE2E1;MITF;FBXO11;PIAS2;PIAS1;TPST2;EEF1A1;UBE2V2;STAM2;DDX5;COPA;INO80D;UBA6;TNKS;COPB1;CUL3;SEL1L;CUL2;CUL1;UAP1;KLHL13;LTBP1;SOCS2;DPP4;VCPIP1;FUT8;UBB;BTBD1;MAN2A1;UBC;NAE1;SUDS3;RAB6A;RAB6B;SOCS5;UBE2F;ANKRD28;SMAD1;UBE2H;SMAD4;SMAD3;SMURF2;GFPT2;NUP153;EIF2S1;ESR1;FBXO30;SMAD7;BMP4;UBE2W;IL6;CNIH1;GNAQ;COPS2;SP3;GNB4;UBA3;UBE2N;MDM2;TGFBI;UBE2K;RAB5A;FBN1;NFE2L2;THRB;USP32;TNFAIP3;LAMC1;CDC73;SEC61A2;ADAMTS5;PPP6C;SUMO1;MAN1A2;ADAMTS1;SUMO2;PGM3;USP47;MMP2;AXIN1;PLAUR;SENP2;RAD23B;TGFBR1;TGFBR2;DNAJC3;VCAN;NSMCE4A;UBE2R2;NUP50;ETF1;BIRC3;B4GALT5;HDAC4;USP14;HDAC2;USP15;UHRF2;TULP4;THBS2;THBS1;RNF2;HDAC7;RAB21;GMDS;NRIP1;DCAF10;EIF4E;EIF4B;OPCML;USP24;ARFGEF2;USP25;SEC24B;SEC24A;FEM1B;GALNT1;LAMB1;KLF4;PML;ASXL1;RAB10;RAB12;ASXL2;RAB18;EIF3E;EIF3F;RECK
Signaling By MET R-HSA-6806834	23/63	6.1829E-09	4.3533E-07	5.5195109	104.326907	LAMA2;LAMA4;RANBP9;PTPRJ;LAMB1;PTPN11;LAMC1;STAM;PTK2;RAP1B;RAP1A;PIK3CA;UBB;UBC;RAPGEF1;LRIG1;GRB2;SOS1;CRK;STAM2;EPS15;PTPN2;ARF6
Transcriptional Activity Of SMAD2/SMAD3:SMAD4 Heterotrimer R-HSA-2173793	20/51	1.4451E-08	9.7325E-07	6.18622012	111.676728	TGIF1;WWTR1;SMAD4;CCNT2;SMAD3;CCNT1;SMURF2;USP9X;UBE2D3;NEDD4L;SMAD7;PPM1A;UBB;MYC;UBC;EP300;MAPK1;SKIL;RNF111;TRIM33
Hemostasis R-HSA-109582	97/576	1.7572E-08	1.1341E-06	1.9742858	35.2547488	APP;PROS1;JMJD1C;PLAT;SLC8A1;DOCK10;DOCK11;ISLR;PLAU;KIF5B;LAMP2;SIRPA;KIF21A;PRKCD;PRKCA;PROCR;STIM1;SELENOP;SERPING1;TAGLN2;ZFPM2;VCL;SDC4;STXBP3;AK3;ITPR2;KLC1;GLG1;RAP1B;RAP1A;ABL1;CARMIL1;TGFB2;YES1;RARRES2;FN1;GNG12;PTK2;MANF;EHD1;GNAQ;MAFF;GNB4;MAFK;GRB2;RAB5A;RCOR1;DOCK4;ITGB3;ATP2A2;THBD;PPP2R5E;KIF13B;PRKG2;ITGAV;KIF1B;JAK2;PRKG1;PDPK1;PLAUR;PLA2G4A;F3;APBB1IP;RAD51B;SLC7A6;PIK3CA;KIF2A;IRF2;CD47;DAGLB;SOS1;CRK;DOCK1;MGLL;CD44;HDAC2;GNAI3;PRCP;SRI;THBS1;PPP2CA;GNA13;PPP2CB;MAPK1;FYN;SLC16A1;PCDH7;PTPN11;VEGFD;ATP2B1;PIKFYVE;WEE1;P2RX4;PDE10A;CAPZA1;CAPZA2;TEK
Signaling By NOTCH1 R-HSA-1980143	24/74	4.2122E-08	2.6099E-06	4.60748936	78.2475785	HDAC4;HDAC2;CREBBP;TLE1;MAML2;MAML1;FBXW7;CUL1;DTX2;PSEN1;RBPJ;HDAC8;HIF1A;HDAC7;MIB1;ADAM17;ITCH;UBB;NBEA;MYC;UBC;NUMB;EP300;TBL1X

Supplementary Table 14: Top 25 significantly enriched Gene Ontology Biological Processes terms for genes differentially expressed in Mfap5+/+ mouse fibroblasts vs Mfap5-/- mouse fibroblasts in day 3 post-wounding

Term	Overlap	P.value	Adjusted.P.value	Odds.Ratio	Combined.Score	Genes
Protein Phosphorylation (GO:0006468)	22/500	1.00575E-05	0.008691922	3.114149331	35.8351162	SMG1;CAMK1D;ROCK2;PRKCE;STK39;PRKCA;YWHAZ;DCLK1;TTBK2;STK3;TGFBR3;CDK8;PPP3CB;WNK1;TAOK1;ABL1;CCL2;FYN;TNIK;PRKACB;CDK14;ATR
Protein Modification Process (GO:0036211)	27/711	1.45529E-05	0.008691922	2.68958687	29.95586401	CL2;FYN;PRKACB;ANXA1;PRKCE;PRKCA;YWHAZ;DCLK1;CDK8;WNK1;PTPRA;TAOK1;TNIK;CDK14;ATR
Regulation Of Focal Adhesion Assembly (GO:0051893)	7/54	1.75262E-05	0.008691922	9.786451292	107.1794076	ROCK2;PTPRA;FYN;THY1;ARHGAP6;THBS1;GPM6B
Extracellular Matrix Organization (GO:0030198)	12/176	1.78662E-05	0.008691922	4.860627178	53.13928317	COL15A1;COL4A2;COL5A1;COL4A1;LUM;COL5A3;ELN;HAS1;SH3PXD2B;PXDN;LAMC1;GPM6B
Negative Regulation Of Protein Localization (GO:1903828)	6/40	3.02546E-05	0.011775079	11.56470588	120.3407484	MFHAS1;ROCK2;YWHAZ;GPM6B;TTBK2;PICALM
Phosphorylation (GO:0016310)	19/429	3.73225E-05	0.012104944	3.113758817	31.74761443	SMG1;ROCK2;PRKCE;STK39;PRKCA;YWHAZ;DCLK1;STK3;CDK8;PPP3CB;WNK1;TAOK1;ABL1;CCL2;FYN;TNIK;PRKACB;CDK14;ATR
(GO:0018209)	11/166	5.17888E-05	0.012817716	4.700470202	46.38582425	SMG1;CAMK1D;GALNT16;WNK1;ROCK2;PRKCE;STK39;PRKCA;PRKACB;TTBK2;ATR
(GO:0016070)	9/111	5.26936E-05	0.012817716	5.820558526	57.33841946	ATXN2L;PNPT1;DDX3X;HNRNPH1;CELF2;PCBP2;HNRNPD;RBMS1;RBM6
Vessel Endothelial Cell Migration (GO:0043536)	6/46	6.83607E-05	0.013339618	9.827	94.24792651	ANXA1;TMSB4X;RHOJ;PRKCA;HIF1A;THBS1
Smooth Muscle Cell Migration (GO:0014909)	3/6	6.85489E-05	0.013339618	64.98679868	623.091025	ITGB3;CCN3;PLAT
Regulation Of RNA Metabolic Process (GO:0051252)	8/91	7.74937E-05	0.013400576	6.343009622	60.03857665	MBNL1;AHNAK;SNRNP70;HNRNPH1;FUS;PCBP2;PDE3A;RC3H2
Mediated Signal Transduction (GO:0051056)	9/118	8.4944E-05	0.013400576	5.444815124	51.03707495	TIAM1;ARHGAP32;AKAP13;TRIO;ARHGEF12;ARHGAP26;KALRN;ARHGAP6;CGNL1
Apoptotic Signaling Pathway (GO:2001237)	7/70	9.6292E-05	0.013400576	7.295057599	67.46560963	DDX3X;EYA3;BCL2;HTT;THBS1;HSPA1B;PF4
Positive Regulation Of Epithelial Cell Migration (GO:0010634)	8/95	0.000105143	0.013400576	6.050142714	55.42045587	ROCK2;ITGB3;PRKCE;ABL1;PRKCA;DOCK1;HIF1A;THBS1
Extracellular Matrix Assembly (GO:0085029)	4/17	0.000109263	0.013400576	20.05196128	182.9090769	ELN;HAS1;PXDN;GPM6B
Regulation Of Epithelial Cell Migration (GO:0010632)	6/50	0.000110179	0.013400576	8.931818182	81.39923303	PRKCE;RHOJ;DOCK1;HIF1A;ARSB;PTPRG
Nervous System Development (GO:0007399)	18/433	0.00013286	0.014781039	2.903463855	25.91694226	NCKAP1;FARP1;MBNL1;SEMA6A;PLXND1;ZBTB16;LPAR1;FBXL17;TRAK1;ARHGAP26;KALRN;DCLK1;SDK1;ZEB2;MARCKS;NFASC;GPM6B;CDK5RAP2
Regulation Of Cell Migration (GO:0030334)	18/434	0.000136721	0.014781039	2.896334135	25.77033448	CLIC4;ANXA1;PLXND1;FZD4;ROCK2;PRKCA;LDLRAD4;THY1;THBS1;TTBK2;TIAM1;CCL7;TMSB4X;DPYSL3;MYADM;FYN;ROR2;ARSB
Peptidyl-Serine Phosphorylation (GO:0018105)	10/158	0.000165192	0.016919126	4.461742148	38.85464812	SMG1;CAMK1D;WNK1;ROCK2;PRKCE;STK39;PRKCA;PRKACB;TTBK2;ATR
Activated MAPK Cascade (GO:0032874)	8/105	0.000211436	0.020572678	5.423649069	45.89269693	MFHAS1;RASSF2;TAOK1;STK39;CCN2;TNIK;PLCB1;MID1
Regulation Of Cell-Substrate Junction Assembly (GO:0090109)	4/21	0.000261814	0.022673298	15.33073627	126.4460286	ROCK2;PTPRA;FYN;GPM6B
(GO:0014812)	3/9	0.000278232	0.022673298	32.48844884	265.98469	ITGB3;CCN3;PLAT
Negative Regulation Of Extrinsic Apoptotic Signaling Pathway In Absence Of Ligand (GO:2001240)	4/22	0.00031616	0.022673298	14.47829286	116.6843449	EYA3;BCL2;HSPA1B;PF4
Transduction In Absence Of Ligand (GO:1901099)	4/22	0.00031616	0.022673298	14.47829286	116.6843449	EYA3;BCL2;HSPA1B;PF4
(GO:0070555)	7/85	0.000326235	0.022673298	5.88765972	47.26550875	ANXA1;CCL7;IL1R1;USP10;CCL2;PRKCA;HIF1A

Supplementary Table 15: Top 25 significantly enriched Reactome terms for genes differentially expressed in Mfap5+/+ mouse fibroblasts vs Mfap5-/- mouse fibroblasts in day 3 post-wounding

Term	Overlap	P.value	Adjusted.P.value	Odds.Ratio	Combined.Score	Genes
Signaling By Rho GTPases, Miro GTPases And RHOBTB3 R-HSA-9716542	39/660	4.9091E-13	3.39709E-10	4.486221933	127.1508181	NCKAP1;DYNC1I2;DOCK4;TRIO;PLXND1;ROCK2;WIPF2;RAP1GDS1;IQGAP2;TRAK1;KALRN;ARHGAP6;NUP160;AKAP13;TUBA1B;ARHGAP42;SLK;ABL1;NHS;RANBP2;FARP1;ACTR2;PRPF38B;ARHGEF12;RALGAPA1;MRTFA;PRKCA;ARHGAP26;YWHAZ;TIAM1;ARHGAP32;DIAPH2;FMNL2;DAAM1;TAOK1;DLC1;RHOJ;DOCK1;PICALM
Signaling By Rho GTPases R-HSA-194315	37/644	4.70286E-12	1.62719E-09	4.32512264	112.8115243	NCKAP1;DYNC1I2;DOCK4;TRIO;PLXND1;ROCK2;WIPF2;IQGAP2;KALRN;ARHGAP6;NUP160;AKAP13;TUBA1B;ARHGAP42;SLK;ABL1;NHS;RANBP2;FARP1;ACTR2;PRPF38B;ARHGEF12;RALGAPA1;MRTFA;PRKCA;ARHGAP26;YWHAZ;TIAM1;ARHGAP32;DIAPH2;FMNL2;DAAM1;TAOK1;DLC1;RHOJ;DOCK1;PICALM
Signal Transduction R-HSA-162582	79/2465	7.34474E-11	1.69419E-08	2.524513406	58.90813534	NCKAP1;DOCK4;TRIO;PLXND1;WIPF2;ITGB3;PLAT;LAMC1;ARHGAP6;DUSP16;AKAP13;TUBA1B;PPP3CB;ARHGAP42;SLK;PRKACB;ACTR2;ANXA1;ARHGEF12;RALGAPA1;PRKCE;TCF12;MRTFA;PRKCA;GAB2;YWHAZ;APBB1IP;ACTA2;TIAM1;DAAM1;COL4A2;HNRNPH1;COL4A1;PARD3;PXDN;RHOJ;COL6A3;PLCB1;DOCK1;DYNC1I2;CALCRL;ROCK2;RAP1GDS1;LPAR1;IQGAP2;TRAK1;KALRN;HIF1A;THBS1;NUP160;ADCY5;HDAC7;PPP3R1;CCL7;ABL1;NHS;CCL2;NCAM1;FYN;ABCA1;RANBP2;FARP1;SMAD1;PRPF38B;FZD4;ARHGAP26;HNRNPM;CDK8;ARHGAP32;DIAPH2;FMNL2;PTPRA;TAOK1;DLC1;PDE3A;BCL2;PTPN2;PF4;PICALM
RHO GTPase Cycle R-HSA-9012999	28/441	2.31258E-10	4.00076E-08	4.702109499	104.3280033	NCKAP1;DOCK4;TRIO;PLXND1;ROCK2;WIPF2;IQGAP2;KALRN;ARHGAP6;AKAP13;TUBA1B;ARHGAP42;SLK;NHS;FARP1;PRPF38B;ARHGEF12;RALGAPA1;ARHGAP26;TIAM1;ARHGAP32;DIAPH2;FMNL2;DAAM1;DLC1;RHOJ;DOCK1;PICALM
RHOA GTPase Cycle R-HSA-8980692	15/147	8.05555E-09	1.11489E-06	7.639019057	142.3676672	FARP1;PRPF38B;TRIO;ARHGEF12;ROCK2;ARHGAP26;KALRN;ARHGAP6;TIAM1;ARHGAP32;AKAP13;ARHGAP42;SLK;DAAM1;DLC1
Extracellular Matrix Organization R-HSA-1474244	20/291	2.54521E-08	2.93548E-06	5.011999071	87.64215312	COL15A1;LUM;ITGB3;PRKCA;PLOD2;LAMC1;THBS1;MFAP5;VCAN;COL4A2;COL5A1;COL4A1;COL5A3;P4HA3;PXDN;CAPN2;COL6A3;NCAM1;CD44;FBN1
Nervous System Development R-HSA-9675108	26/545	3.3738E-07	3.33524E-05	3.430704652	51.12455348	TRIO;PLXND1;ROCK2;ITGB3;LAMC1;KALRN;PPP3CB;DPYSL3;ABL1;NCAM1;FYN;SLIT3;PRKACB;TEAD1;ACTR2;SEMA6A;ARHGEF12;PRKCA;GAB2;TIAM1;NFASC;COL4A2;COL4A1;PTPRA;COL6A3;DOCK1
Axon Guidance R-HSA-422475	25/519	4.79301E-07	4.14595E-05	3.457864481	50.31516797	TRIO;PLXND1;ROCK2;ITGB3;LAMC1;KALRN;PPP3CB;DPYSL3;ABL1;NCAM1;FYN;SLIT3;PRKACB;ACTR2;SEMA6A;ARHGEF12;PRKCA;GAB2;TIAM1;NFASC;COL4A2;COL4A1;PTPRA;COL6A3;DOCK1
RAC1 GTPase Cycle R-HSA-9013149	14/178	6.57513E-07	5.05554E-05	5.709572335	81.2746303	NCKAP1;FARP1;DOCK4;TRIO;WIPF2;IQGAP2;ARHGAP26;KALRN;TIAM1;ARHGAP32;ARHGAP42;DLC1;NHS;DOCK1
RHOB GTPase Cycle R-HSA-9013026	9/69	1.03516E-06	7.16332E-05	9.916161616	136.6541593	AKAP13;ARHGAP32;PRPF38B;SLK;ARHGEF12;DAAM1;ROCK2;DLC1;ARHGAP26
RHOC GTPase Cycle R-HSA-9013106	9/73	1.6809E-06	0.000105744	9.294507576	123.5814452	AKAP13;ARHGAP32;SLK;ARHGEF12;FMNL2;DAAM1;ROCK2;DLC1;ARHGAP26
CDC42 GTPase Cycle R-HSA-9013148	12/149	3.25265E-06	0.00018757	5.826605095	73.62521595	TIAM1;ARHGAP32;FARP1;ARHGAP42;TRIO;ARHGEF12;FMNL2;DAAM1;WIPF2;DLC1;IQGAP2;ARHGAP26

RHO GTPase Effectors R-HSA-195258	16/269	4.29254E-06	0.000228495	4.239552951	52.39507658	RANBP2;NCKAP1;DYNC1I2;ACTR2;ROCK2;WIPF2;MRTFA;PRKCA;IQGAP2;YWHAZ;NUP160;DIAPH2;FMNL2;DAAM1;TAOK1;ABL1
RAC2 GTPase Cycle R-HSA-9013404	9/87	7.36562E-06	0.000361125	7.620823621	90.06813384	NCKAP1;TIAM1;ARHGAP32;DOCK4;ARHGAP42;TRIO;NHS;ARHGAP26;DOCK1
Collagen Biosynthesis And Modifying Enzymes R-HSA-1650814	8/67	8.1654E-06	0.000361125	8.934137186	104.6688175	COL15A1;COL4A2;COL5A1;COL4A1;COL5A3;P4HA3;COL6A3;PLOD2
Developmental Biology R-HSA-1266738	36/1073	8.44652E-06	0.000361125	2.398842816	28.02269774	CEBPB;TRIO;PLXND1;CHD9;ROCK2;ITGB3;LPL;LAMC1;KALRN;PPP3CB;DPYSL3;ABL1;NCAM1;FYN;SLIT3;PRKACB;TEAD1;KDM6A;ACTR2;SEMA6A;ARHGEF12;TCF12;LGI2;EBF1;PRKCA;GAB2;MED13L;TIAM1;CDK8;NFASC;COL4A2;COL4A1;PTPRA;COL6A3;ZFPM2;DOCK1
Signaling By Receptor Tyrosine Kinases R-HSA-9006934	22/496	8.87156E-06	0.000361125	3.141082784	36.53914885	NCKAP1;ROCK2;PRKCE;ITGB3;TCF12;PRKCA;PLAT;GAB2;LAMC1;HIF1A;THBS1;TIAM1;HNRNPM;COL4A2;HNRNPH1;COL4A1;PXDN;COL6A3;FYN;PRKACB;DOCK1;PTPN2
Collagen Formation R-HSA-1474290	9/90	9.74385E-06	0.000374597	7.33744856	84.66589719	COL15A1;COL4A2;COL5A1;COL4A1;COL5A3;P4HA3;PXDN;COL6A3;PLOD2
Assembly Of Collagen Fibrils And Other Multimeric Structures R-HSA-2022090	7/57	2.5159E-05	0.000916317	9.197859532	97.40804495	COL15A1;COL4A2;COL5A1;COL4A1;COL5A3;PXDN;COL6A3
Collagen Chain Trimerization R-HSA-8948216	6/44	5.28565E-05	0.001828834	10.34526316	101.8794332	COL15A1;COL4A2;COL5A1;COL4A1;COL5A3;COL6A3
Integrin Cell Surface Interactions R-HSA-216083	7/66	6.59021E-05	0.002171631	7.791224987	75.00877458	COL4A2;COL4A1;LUM;ITGB3;THBS1;CD44;FBN1
RAC3 GTPase Cycle R-HSA-9013423	8/93	9.04463E-05	0.002721254	6.193130675	57.66271758	NCKAP1;TIAM1;ARHGAP32;ARHGAP42;TRIO;NHS;ARHGAP26;ARHGAP6
VEGFA-VEGFR2 Pathway R-HSA-4420097	8/93	9.04463E-05	0.002721254	6.193130675	57.66271758	NCKAP1;ROCK2;ITGB3;PXDN;PRKCA;FYN;PRKACB;DOCK1
HDL Assembly R-HSA-8963896	3/7	0.000118601	0.003419659	48.73762376	440.5757726	ABCA1;PRKCA;PRKACB
EPHB-mediated Forward Signaling R-HSA-3928662	5/34	0.000156885	0.004342576	11.26417688	98.67416225	ACTR2;TIAM1;ROCK2;FYN;KALRN

Supplementary Table 16: Top 25 significantly enriched Gene Ontology Biological Processes terms for genes differentially expressed in Mfap5-/- mouse fibroblasts vs Mfap5+/+ mouse fibroblasts in day 3 post-wounding

Term	Overlap	P.value	Adjusted.P.value	Odds.Ratio	Combined.Score	Genes
Cytoplasmic Translation (GO:0002181)	66/93	8.36242E-77	1.91917E-73	78.92939428	13826.4807	RPL4;RPL5;RPL30;RPL3;RPL32;RPL31;RPL34;RPLP0;RPL10A;RPL8;RPL9;RPL6;RPL7;RPS4X;RPL7A;RPS14;RPS17;RPS16;RPL18A;RPS19;RPL36AL;RPS18;RACK1;RPL36;RPL35;RPL38;RPL37;RPS11;RPL39;RPS10;RPS13;RPL21;RPS7;RPS5;RPL22;RPS6;RPL13A;RPSA;RPL24;RPL26;RPL29;RPL28;UBA52;RPL10;RPL12;RPL11;RPS15A;RPL14;RPS3;RPL13;RPL15;RPS2;RPL18;RPS27A;RPL17;RPL19;RPS25;RPS27;RPS29;EIF3I;RPL27A;RPS20;FAU;RPS21;RPS24;RPS23
Peptide Biosynthetic Process (GO:0043043)	72/158	6.63349E-64	7.61193E-61	27.22344437	3960.284683	RPL4;RPL5;RPL30;RPL3;MRPS16;RPL32;RPL31;RPL34;RPLP0;RPL10A;RPL8;MRPL34;RPL9;RPL6;RPL7;RPS4X;RPS14;RPL7A;RPS17;RPS16;RPL18A;RPS19;RPL36AL;RPS18;RPL36;RPL35;RPL38;RPL37;RPS11;RPL39;RPS10;RPS13;RPL21;RPS7;RPS5;RPL22;RPS6;MRPS21;RPL13A;RPSA;MRPL43;EEF1A1;MRPL52;RPL24;RPL26;RPL29;RPL28;RPL10;RPL12;RPL11;RRBP1;RPS15A;RPL14;RPS3;RPL13;RPL15;RPS2;RPL18;RPS27A;RPL17;RPL19;MRPL27;MRPL23;RPS25;RPS27;RPS29;RPL27A;RPS20;FAU;RPS21;RPS24;RPS23
Macromolecule Biosynthetic Process (GO:0009059)	75/183	2.68556E-62	2.05446E-59	22.6702509	3214.015549	RPL4;RPL5;RPL30;RPL3;MRPS16;RPL32;RPL31;RPL34;RPLP0;RPL10A;RPL8;MRPL34;RPL9;RPL6;RPL7;EEF1B2;RPS4X;RPS14;RPL7A;RPS17;RPS16;RPL18A;RPS19;RPL36AL;RPS18;RPL36;RPL35;RPL38;RPL37;RPS11;RPL39;RPS10;RPS13;RPL21;RPS7;RPS5;RPL22;RPS6;MRPS21;RPL13A;RPSA;MRPL43;EEF1A1;MRPL52;RPL24;RPL26;RPL29;RPL28;RPL10;RPL12;RPL11;RRBP1;RPS15A;RPL14;RPS3;RPL13;RPL15;RPS2;RPL18;RPS27A;RPL17;RPL19;MRPL27;INHBA;EEF2;MRPL23;RPS25;RPS27;RPS29;RPL27A;RPS20;FAU;RPS21;RPS24;RPS23
Translation (GO:0006412)	82/234	1.42852E-61	8.19611E-59	17.7822391	2491.31045	RPL4;RPL5;RPL30;RPL3;MRPS16;RPL32;RPL31;RPL34;RPLP0;RPL8;MRPL34;RPL10A;RPL9;RPL6;RPL7;EEF1B2;RPS4X;RPS14;RPL7A;RPS17;RPS16;RPL18A;RPS19;RPL36AL;RPS18;RPL36;RACK1;RPL35;RPL38;RPL37;RPS11;RPL39;RPS10;RPS13;RPL21;RPS7;RPS5;RPL22;RPS6;MRPS21;RPL13A;RPSA;MRPL43;MRPL52;EEF1A1;RPL24;RPL26;AURKAIP1;RPL29;RPL28;UBA52;RPL10;RPL12;RPL11;MRPL17;RRBP1;MRPL54;RPS15A;RPL14;RPS3;RPL13;RPL15;RPS2;RPL18;RPS27A;RPL17;RPL19;GADD45GIP1;NDUFA7;MRPL27;MRPL23;EEF2;MRPL30;RPS25;RPS27;RPS29;RPL27A;RPS20;FAU;RPS21;RPS24;RPS23
Gene Expression (GO:0010467)	75/296	7.75081E-45	3.55762E-42	11.01356698	1118.63183	RPL4;RPL5;RPL30;RPL3;MRPS16;RPL32;RPL31;RPL34;RPLP0;RPL8;MRPL34;RPL10A;RPL9;RPL6;RPL7;RBM3;RPS4X;RPS14;RPL7A;RPS17;RPS16;RPL18A;RPS19;RPL36AL;RPS18;RPL36;RPL35;RPL38;RPL37;RPS11;RPL39;RPS10;RPS13;RPL21;RPS7;RPS5;RPL22;RPS6;MRPS21;RPL13A;RPSA;MRPL43;MRPL52;EEF1A1;RPL24;RPL26;RPL29;RPL28;RPL10;RPL12;RPL11;RRBP1;EXOSC4;RPS15A;RPL14;RPS3;RPL13;RPL15;RPS2;RPL18;RPS27A;RPL17;RPL19;MRPL27;MRPL23;RPS25;RPS27;RPS29;RPL27A;RPS20;STUB1;FAU;RPS21;RPS24;RPS23
Ribonucleoprotein Complex Biogenesis (GO:0022613)	22/118	4.36682E-11	1.67031E-08	6.867860852	163.8287092	RPL5;RPS7;RPS5;RPS6;RPL11;GTF3A;RPL7;RPS4X;RPS14;RPS25;RPS17;RPS16;RPS27;RPS15A;RPS19;RPL14;RPL38;RPL26;RPS11;RPS13;RPS24;RPS23
Ribosome Biogenesis (GO:0042254)	22/155	9.4062E-09	3.08389E-06	4.947719767	91.44324389	RPL5;RPS7;RPS5;RPS6;RPL11;GTF3A;RPL7;RPS4X;RPS14;RPS25;RPS17;RPS16;RPS27;EXOSC4;RPS15A;RPS19;RPL14;RPL26;RPS11;RPS13;RPS24;RPS23
Ribosomal Small Subunit Biogenesis (GO:0042274)	16/84	1.42507E-08	4.08817E-06	6.996368918	126.39962	RPS7;RPS5;RPS6;RPSA;RPS4X;RPS14;RPS25;RPS17;RPS16;RPS15A;RPS27;RPS19;RPS11;RPS13;RPS24;RPS23
Cellular Respiration (GO:0045333)	15/85	1.16936E-07	2.98186E-05	6.361215056	101.5354458	COX8A;NDUFB9;NDUFA13;NDUFA7;NDUFB11;NDUFB5;NDUFA3;NDUFA1;UQCR11;UQCR10;COX5B;NDUFS3;ETFRF1;NDUFV3;NDUFV1
rRNA Processing (GO:0006364)	16/101	2.11344E-07	4.85035E-05	5.592156863	85.95020661	RPL5;RPS7;RPS6;RPL11;RPL7;RPS14;RPS25;RPS17;RPS16;RPS27;EXOSC4;RPS19;EIF6;RPL14;RPL26;RPS24

rRNA Metabolic Process (GO:0016072)	15/91	2.96548E-07	6.18707E-05	5.857189198	88.03974456	RPL5;RPS7;RPS6;RPL11;GTF3A;RPL7;RPS25;RPS17;RPS16;EXOSC4;RPS27;RPS19;RPL14;RPL26;RPS24
Ribosome Assembly (GO:0042255)	11/50	5.7107E-07	0.000109217	8.334982526	119.8216614	RPL5;RPS14;RPS27;RPS19;RPL10;EIF6;RPS5;RPLP0;RPL11;RPSA;RPL6
Endoplasmic Reticulum To Golgi Vesicle-Mediated Transport (GO:0006888)	16/115	1.28703E-06	0.00022721	4.797855094	65.07415645	TMED9;SAR1B;YIPF4;TEX261;IER3IP1;YIF1A;YIF1B;LMAN1;TRAPPC6A;TMED3;KDELR2;TMED1;KDELR3;ERGIC3;COPE;BET1
Aerobic Electron Transport Chain (GO:0019646)	12/68	2.15529E-06	0.000353314	6.336546889	82.67662229	COX8A;NDUFB9;NDUFA7;NDUFB5;NDUFA3;NDUFS3;NDUFA1;UQCR11;UQCR10;NDUFV3;COX5B;NDUFV1
Mitochondrial ATP Synthesis Coupled Electron Transport (GO:0042775)	12/70	2.96376E-06	0.000453455	6.11741062	77.86884267	COX8A;NDUFB9;NDUFA7;NDUFB5;NDUFA3;NDUFS3;NDUFA1;UQCR11;UQCR10;NDUFV3;COX5B;NDUFV1
Glycolytic Process (GO:0006096)	8/29	3.27297E-06	0.000469467	11.21660859	141.6636686	LDHA;PFKL;TPI1;PKM;PGAM1;PGK1;ENO1;GAPDH
Positive Regulation Of Intrinsic Apoptotic Signaling Pathway (GO:2001244)	9/39	4.0282E-06	0.000543807	8.842442748	109.8425113	SLC9A3R1;FIS1;UBB;RPS7;RACK1;RPS3;NUPR1;S100A9;S100A8
ncRNA Processing (GO:0034470)	14/100	5.46208E-06	0.000696416	4.821109123	58.42065759	RPL5;RPS7;RPS6;RPL11;RPL7;RPS25;RPS17;RPS16;RPS27;EXOSC4;RPS19;RPL14;RPL26;RPS24
Carbohydrate Catabolic Process (GO:0016052)	9/41	6.2759E-06	0.000758062	8.288931298	99.29140382	LDHA;PFKL;TPI1;PKM;PGAM1;PGK1;ENO1;GAPDH;DERA
Proton Motive Force-Driven Mitochondrial ATP Synthesis (GO:0042776)	10/53	8.15897E-06	0.000936241	6.860465116	80.3799065	NDUFB9;NDUFA13;NDUFA7;NDUFB11;NDUFB5;NDUFA3;NDUFS3;NDUFA1;NDUFV3;NDUFV1
Ribosomal Large Subunit Biogenesis (GO:0042273)	10/54	9.71849E-06	0.001062093	6.704197943	77.3763653	RPL5;RPL10;EIF6;RPLP0;RPL11;GTF3A;RPL14;RPL26;RPL6;RPL7
protein-RNA Complex Assembly (GO:0022618)	17/150	1.0474E-05	0.00109263	3.793692113	43.50080031	RPL5;RAMAC;RPL10;RPS5;RPLP0;RPL11;RPSA;RPL6;RPS14;RPS27;RPS19;EIF6;EIF3K;EIF3I;EIF3H;RPL38;EIF3F
Maintenance Of Protein Localization In Endoplasmic Reticulum (GO:0035437)	5/11	1.55432E-05	0.001550942	24.44360142	270.6367888	OS9;RER1;INSIG1;KDELR2;KDELR3
Aerobic Respiration (GO:0009060)	10/59	2.19769E-05	0.002101538	6.018535855	64.55192634	NDUFB9;NDUFA13;NDUFA7;NDUFB11;NDUFB5;NDUFA3;NDUFS3;NDUFA1;NDUFV3;NDUFV1
Regulation Of Intrinsic Apoptotic Signaling Pathway (GO:2001242)	9/48	2.44166E-05	0.00224144	6.798708162	72.20397288	SLC9A3R1;FIS1;NDUFA13;RACK1;NDUFS3;NUPR1;P4HB;S100A9;S100A8

Supplementary Table 17: Top 25 significantly enriched reactome terms for genes differentially expressed in Mfap5-/- mouse fibroblasts vs Mfap5+/+ mouse fibroblasts in day 7 post-wounding

Term	Overlap	P.value	Adjusted.P.value	Odds.Ratio	Combined.Score	Genes
Peptide Chain Elongation R-HSA-156902	63/86	8.36E-75	7.0573E-72	88.02134124	15013.83746	RPL4;RPL5;RPL30;RPL3;RPL32;RPL31;RPL34;RPLP0;RPL10A;RPL8;RPL9;RPL6;RPL7;RPS4X;RPL7A;RPS14;RPS17;RPS16;RPL18A;RPS19;RPL36AL;RPL36;RPL35;RPL38;RPL37;RPS11;RPL39;RPS10;RPS13;RPL21;RPS7;RPL23;RPS5;RPL22;RPS6;RPL13A;RPSA;EEF1A1;RPL26;RPL29;RPL28;UBA52;RPL12;RPL11;RPL36A;RPS15A;RPL14;RPS3;RPL15;RPS2;RPL18;RPS27A;RPL19;EEF2;RPS25;RPS27;RPS29;RPL27A;RPS20;FAU;RPS21;RPS24;RPS23
Eukaryotic Translation Elongation R-HSA-156842	64/90	1.4157E-74	7.0573E-72	79.22051282	13470.94666	RPL4;RPL5;RPL30;RPL3;RPL32;RPL31;RPL34;RPLP0;RPL10A;RPL8;RPL9;RPL6;RPL7;EEF1B2;RPS4X;RPL7A;RPS14;RPS17;RPS16;RPL18A;RPS19;RPL36AL;RPL36;RPL35;RPL38;RPL37;RPS11;RPL39;RPS10;RPS13;RPL21;RPS7;RPL23;RPS5;RPL22;RPS6;RPL13A;RPSA;EEF1A1;RPL26;RPL29;RPL28;UBA52;RPL12;RPL11;RPL36A;RPS15A;RPL14;RPS3;RPL15;RPS2;RPL18;RPS27A;RPL19;EEF2;RPS25;RPS27;RPS29;RPL27A;RPS20;FAU;RPS21;RPS24;RPS23
Formation Of A Pool Of Free 40S Subunits R-HSA-72689	65/98	1.5679E-72	5.2105E-70	63.47422472	10494.60103	RPL4;RPL5;RPL30;RPL3;RPL32;RPL31;RPL34;RPLP0;RPL10A;RPL8;RPL9;RPL6;RPL7;RPS4X;RPS14;RPL7A;RPS17;RPS16;RPL18A;RPS19;RPL36AL;RPL36;RPL35;RPL38;RPL37;RPS11;RPL39;RPS10;RPS13;RPL21;RPS7;RPL23;RPS5;RPL22;RPS6;RPL13A;RPSA;RPL26;RPL29;RPL28;UBA52;RPL12;RPL11;RPL36A;RPS15A;RPL14;RPS3;RPL15;RPS2;RPL18;RPS27A;RPL19;RPS25;RPS27;EIF3K;RPS29;RPL27A;EIF3I;EIF3H;RPS20;EIF3F;FAU;RPS21;RPS24;RPS23
SRP-dependent Cotranslational Protein Targeting To Membrane R-HSA-1799339	67/108	9.1979E-72	2.2926E-69	52.81550027	8638.880724	RPL4;RPL5;RPL30;RPL3;RPL32;RPL31;RPL34;RPLP0;RPL10A;RPL8;RPL9;RPL6;RPL7;RPS4X;RPS14;RPL7A;SEC61A1;RPS17;RPS16;RPL18A;RPS19;RPL36AL;SEC61G;RPL36;RPL35;RPL38;RPL37;RPS11;RPL39;RPS10;RPS13;RPL21;RPS7;SSR4;RPL23;RPS5;SSR2;RPL22;RPS6;RPL13A;RPSA;RPL26;RPL29;RPL28;UBA52;RPL12;RPL11;RPL36A;RPS15A;RPL14;RPS3;RPL15;RPS2;RPL18;RPS27A;RPL19;SEC11C;RPS25;SPCS2;RPS27;RPS29;RPL27A;RPS20;FAU;RPS21;RPS24;RPS23

Viral mRNA Translation R-HSA-192823	62/90	7.803E-71	1.5559E-68	71.01898434	11464.52598	RPL4;RPL5;RPL30;RPL3;RPL32;RPL31;RPL34;RPLP0;RPL10A;RPL8;RPL9;RPL6;RPL7;RPS4X;RPL7A;RPS14;RPS17;RPS16;RPL18A;RPS19;RPL36AL;RPL36;RPL35;RPL38;RPL37;RPS11;RPL39;RPS10;RPS13;RPL21;RPS7;RPL23;RPS5;RPL22;RPS6;RPL13A;RPSA;DNAJC3;RPL26;RPL29;RPL28;UBA52;RPL12;RPL11;RPL36A;RPS15A;RPL14;RPS3;RPL15;RPS2;RPL18;RPS27A;RPL19;RPS25;RPS27;RPS29;RPL27A;RPS20;FAU;RPS21;RPS24;RPS23
Eukaryotic Translation Termination R-HSA-72764	61/90	5.3491E-69	7.6187E-67	67.34871619	10587.31475	RPL4;RPL5;RPL30;RPL3;RPL32;RPL31;RPL34;RPLP0;RPL10A;RPL8;RPL9;RPL6;RPL7;RPS4X;RPL7A;RPS14;RPS17;RPS16;RPL18A;RPS19;RPL36AL;RPL36;RPL35;RPL38;RPL37;RPS11;RPL39;RPS10;RPS13;RPL21;RPS7;RPL23;RPS5;RPL22;RPS6;RPL13A;RPSA;RPL26;RPL29;RPL28;UBA52;RPL12;RPL11;RPL36A;RPS15A;RPL14;RPS3;RPL15;RPS2;RPL18;RPS27A;RPL19;RPS25;RPS27;RPS29;RPL27A;RPS20;FAU;RPS21;RPS24;RPS23
Selenocysteine Synthesis R-HSA-2408557	61/90	5.3491E-69	7.6187E-67	67.34871619	10587.31475	RPL4;RPL5;RPL30;RPL3;RPL32;RPL31;RPL34;RPLP0;RPL10A;RPL8;RPL9;RPL6;RPL7;RPS4X;RPL7A;RPS14;RPS17;RPS16;RPL18A;RPS19;RPL36AL;RPL36;RPL35;RPL38;RPL37;RPS11;RPL39;RPS10;RPS13;RPL21;RPS7;RPL23;RPS5;RPL22;RPS6;RPL13A;RPSA;RPL26;RPL29;RPL28;UBA52;RPL12;RPL11;RPL36A;RPS15A;RPL14;RPS3;RPL15;RPS2;RPL18;RPS27A;RPL19;RPS25;RPS27;RPS29;RPL27A;RPS20;FAU;RPS21;RPS24;RPS23
Cap-dependent Translation Initiation R-HSA-72737	67/116	1.0202E-68	1.2715E-66	44.17423854	6915.730641	RPL4;RPL5;RPL30;RPL3;RPL32;RPL31;RPL34;RPLP0;RPL10A;RPL8;RPL9;RPL6;RPL7;RPS4X;RPS14;RPL7A;RPS17;RPS16;RPL18A;RPS19;RPL36AL;RPL36;RPL35;RPL38;RPL37;RPS11;RPL39;RPS10;RPS13;RPL21;RPS7;RPL23;RPS5;RPL22;RPS6;RPL13A;RPSA;RPL26;RPL29;RPL28;UBA52;RPL12;RPL11;RPL36A;RPS15A;EIF4EBP1;RPL14;RPS3;RPL15;RPS2;RPL18;RPS27A;RPL19;EIF2B2;RPS25;RPS27;EIF3K;RPS29;RPL27A;EIF3I;EIF3H;RPS20;EIF3F;FAU;RPS21;RPS24;RPS23
L13a-mediated Translational Silencing Of Ceruloplasmin Expression R-HSA-156827	65/108	2.3472E-68	2.6001E-66	48.68754125	7581.749354	RPL4;RPL5;RPL30;RPL3;RPL32;RPL31;RPL34;RPLP0;RPL10A;RPL8;RPL9;RPL6;RPL7;RPS4X;RPS14;RPL7A;RPS17;RPS16;RPL18A;RPS19;RPL36AL;RPL36;RPL35;RPL38;RPL37;RPS11;RPL39;RPS10;RPS13;RPL21;RPS7;RPL23;RPS5;RPL22;RPS6;RPL13A;RPSA;RPL26;RPL29;RPL28;UBA52;RPL12;RPL11;RPL36A;RPS15A;RPL14;RPS3;RPL15;RPS2;RPL18;RPS27A;RPL19;RPS25;RPS27;EIF3K;RPS29;RPL27A;EIF3I;EIF3H;RPS20;EIF3F;FAU;RPS21;RPS24;RPS23

Nonsense Mediated Decay (NMD) Independent Of Exon Junction Complex (EJC) R-HSA-975956	61/92	4.5342E-68	4.5206E-66	62.99711122	9768.592841	RPL4;RPL5;RPL30;RPL3;RPL32;RPL31;RPL34;RPLP0;RPL10A;RPL8;RPL9;RPL6;RPL7;RPS4X;RPL7A;RPS14;RPS17;RPS16;RPL18A;RPS19;RPL36AL;RPL36;RPL35;RPL38;RPL37;RPS11;RPL39;RPS10;RPS13;RPL21;RPS7;RPL23;RPS5;RPL22;RPS6;RPL13A;RPSA;RPL26;RPL29;RPL28;UBA52;RPL12;RPL11;RPL36A;RPS15A;RPL14;RPS3;RPL15;RPS2;RPL18;RPS27A;RPL19;RPS25;RPS27;RPS29;RPL27A;RPS20;FAU;RPS21;RPS24;RPS23
GTP Hydrolysis And Joining Of 60S Ribosomal Subunit R-HSA-72706	65/109	5.6429E-68	5.1145E-66	47.57853999	7367.31724	RPL4;RPL5;RPL30;RPL3;RPL32;RPL31;RPL34;RPLP0;RPL10A;RPL8;RPL9;RPL6;RPL7;RPS4X;RPS14;RPL7A;RPS17;RPS16;RPL18A;RPS19;RPL36AL;RPL36;RPL35;RPL38;RPL37;RPS11;RPL39;RPS10;RPS13;RPL21;RPS7;RPL23;RPS5;RPL22;RPS6;RPL13A;RPSA;RPL26;RPL29;RPL28;UBA52;RPL12;RPL11;RPL36A;RPS15A;RPL14;RPS3;RPL15;RPS2;RPL18;RPS27A;RPL19;RPS25;RPS27;EIF3K;RPS29;RPL27A;EIF3I;EIF3H;RPS20;EIF3F;FAU;RPS21;RPS24;RPS23
Response Of EIF2AK4 (GCN2) To Amino Acid Deficiency R-HSA-9633012	62/98	3.1908E-67	2.651E-65	55.21410114	8453.992927	RPL4;RPL5;RPL30;RPL3;RPL32;RPL31;RPL34;RPLP0;RPL10A;RPL8;RPL9;RPL6;RPL7;RPS4X;RPS14;RPL7A;RPS17;RPS16;RPL18A;RPS19;RPL36AL;RPL36;RPL35;RPL38;RPL37;RPS11;RPL39;RPS10;RPS13;RPL21;RPS7;RPL23;RPS5;RPL22;RPS6;RPL13A;RPSA;RPL26;RPL29;RPL28;UBA52;RPL12;RPL11;RPL36A;RPS15A;RPL14;RPS3;RPL15;RPS2;RPL18;RPS27A;RPL19;RPS25;RPS27;GDNF;RPS29;RPL27A;RPS20;FAU;RPS21;RPS24;RPS23
Nonsense Mediated Decay (NMD) Enhanced By Exon Junction Complex (EJC) R-HSA-975957	62/112	5.3635E-62	4.1134E-60	39.72531561	5604.473858	RPL4;RPL5;RPL30;RPL3;RPL32;RPL31;RPL34;RPLP0;RPL10A;RPL8;RPL9;RPL6;RPL7;RPS4X;RPS14;RPL7A;RPS17;RPS16;RPL18A;RPS19;RPL36AL;RPL36;RPL35;RPL38;RPL37;RPS11;RPL39;RPS10;RPS13;RPL21;RPS7;RPL23;RPS5;RPL22;RPS6;RPL13A;RPSA;RPL26;RPL29;RPL28;UBA52;RPL12;RPL11;RPL36A;RPS15A;RPL14;RPS3;RPL15;RPS2;RPL18;RPS27A;RPL19;PNRC2;RPS25;RPS27;RPS29;RPL27A;RPS20;FAU;RPS21;RPS24;RPS23
Translation R-HSA-72766	87/281	4.1874E-60	2.982E-58	14.87746788	2034.092369	RPL4;RPL5;RPL30;RPL3;MRPS16;RPL32;RPL31;RPL34;RPLP0;RPL8;MRPL34;RPL10A;RPL9;RPL6;RPL7;EEF1B2;RPS4X;RPS14;RPL7A;SEC61A1;RPS17;RPS16;RPL18A;RPS19;RPL36AL;SEC61G;RPL36;RPL35;RPL38;RPL37;RPS11;RPL39;RPS10;RPS13;RPL21;SSR4;RPS7;RPL23;SSR2;RPS5;RPL22;RPS6;MRPS21;RPL13A;RPSA;MRPL43;MRPL52;EEF1A1;RPL26;AURKAIP1;RPL29;RPL28;UBA52;RPL12;RPL11;RPL36A;MRPL17;MRPL54;RPS15A;RPL14;RPS3;EIF4EBP1;RPL15;RPS2;RPL18;RPS27A;RPL19;SEC11C;GADD45GIP1;EIF2B2;MRPL23;EEF2;MRPL30;RPS25;SPCS2;RPS27;EIF3K;RPS29;RPL27A;EIF3I;EIF3H;RPS20;FAU;EIF3F;RPS21;RPS24;RPS23

Selenoamino Acid Metabolism R-HSA-2408522	61/114	9.1901E-60	6.1083E-58	36.80537564	5003.211599	RPL4;RPL5;RPL30;RPL3;RPL32;RPL31;RPL34;RPLP0;RPL10A;RPL8;RPL9;RPL6;RPL7;RPS4X;RPS14;RPL7A;RPS17;RPS16;RPL18A;RPS19;RPL36AL;RPL36;RPL35;RPL38;RPL37;RPS11;RPL39;RPS10;RPS13;RPL21;RPS7;RPL23;RPS5;RPL22;RPS6;RPL13A;RPSA;RPL26;RPL29;RPL28;UBA52;RPL12;RPL11;RPL36A;RPS15A;RPL14;RPS3;RPL15;RPS2;RPL18;RPS27A;RPL19;RPS25;RPS27;RPS29;RPL27A;RPS20;FAU;RPS21;RPS24;RPS23
Influenza Viral RNA Transcription And Replication R-HSA-168273	64/137	1.0509E-57	6.5482E-56	28.14684932	3692.802901	RPL4;RPL5;RPL30;RPL3;RPL32;RPL31;RPL34;RPLP0;RPL10A;RPL8;RPL9;RPL6;RPL7;RPS4X;RPS14;RPL7A;RPS17;RPS16;RPL18A;RPS19;RPL36AL;RPL36;RPL35;RPL38;RPL37;RPS11;RPL39;RPS10;RPS13;RPL21;RPS7;RPL23;RPS5;RPL22;RPS6;RPL13A;RPSA;DNAJC3;RPL26;RPL29;RPL28;UBA52;RPL12;RPL11;RPL36A;RPS15A;RPL14;RPS3;RPL15;RPS2;RPL18;RPS27A;POLR2J;POLR2L;RPL19;RPS25;RPS27;RPS29;RPL27A;RPS20;FAU;RPS21;RPS24;RPS23
Regulation Of Expression Of SLITs And ROBOs R-HSA-9010553	68/167	2.9123E-56	1.708E-54	22.16995458	2835.001033	RPL4;RPL5;RPL30;RPL3;RPL32;RPL31;RPL34;RPLP0;RPL10A;RPL8;RPL9;RPL6;RPL7;RPS4X;RPS14;RPL7A;RPS17;PSMD7;RPS16;RPL18A;RPS19;RPL36AL;RPL36;RPL35;RPL38;ELOB;RPL37;RPS11;RPL39;RPS10;RPS13;RPL21;RPS7;RPL23;RPS5;RPL22;RPS6;RPL13A;RPSA;RPL26;RPL29;RPL28;UBA52;RPL12;RPL11;RPL36A;RPS15A;UBB;PSMB2;UBC;PSMB1;RPL14;RPS3;RPL15;RPS2;RPL18;RPS27A;RPL19;RPS25;RPS27;RPS29;RPL27A;PSMC2;RPS20;FAU;RPS21;RPS24;RPS23
Cellular Response To Starvation R-HSA-9711097	65/153	2.9974E-55	1.6602E-53	23.7350129	2979.798448	RPL4;RPL5;RPL30;RPL3;RPL32;RPL31;RPL34;RPLP0;RPL10A;RPL8;RPL9;RPL6;RPL7;RPS4X;RPS14;RPL7A;RPS17;RPS16;RPL18A;RPS19;RPL36AL;RPL36;RPL35;RPL38;RPL37;RPS11;RPL39;RPS10;RPS13;ATP6V0B;RPL21;RPS7;RPL23;RPS5;RPL22;RPS6;RPL13A;RPSA;RPL26;RPL29;RPL28;UBA52;RPL12;RPL11;RPL36A;RPS15A;RPL14;RPS3;RPL15;RPS2;RPL18;RPS27A;ATP6V1F;RPL19;RPS25;RPS27;GDNF;RPS29;RPL27A;RPS20;LAMTOR2;FAU;RPS21;RPS24;RPS23
Influenza Infection R-HSA-168255	64/157	5.202E-53	2.7297E-51	22.07082437	2657.061522	RPL4;RPL5;RPL30;RPL3;RPL32;RPL31;RPL34;RPLP0;RPL10A;RPL8;RPL9;RPL6;RPL7;RPS4X;RPS14;RPL7A;RPS17;RPS16;RPL18A;RPS19;RPL36AL;RPL36;RPL35;RPL38;RPL37;RPS11;RPL39;RPS10;RPS13;RPL21;RPS7;RPL23;RPS5;RPL22;RPS6;RPL13A;RPSA;DNAJC3;RPL26;RPL29;RPL28;UBA52;RPL12;RPL11;RPL36A;RPS15A;RPL14;RPS3;RPL15;RPS2;RPL18;RPS27A;POLR2J;POLR2L;RPL19;RPS25;RPS27;RPS29;RPL27A;RPS20;FAU;RPS21;RPS24;RPS23

Signaling By ROBO Receptors R-HSA-376176	68/209	1.3601E-48	6.7802E-47	15.53215289	1711.899668	RPL4;RPL5;RPL30;RPL3;RPL32;RPL31;RPL34;RPLP0;RPL8;RPL10A;RPL9;RPL6;RPL7;RPS4X;RPS14;RPL7A;RPS17;PSMD7;RPS16;RPL18A;RPS19;RPL36AL;RPL36;RPL35;RPL38;ELOB;RPL37;RPS11;RPL39;RPS10;RPS13;RPL21;RPS7;RPL23;RPS5;RPL22;RPS6;RPL13A;RPSA;RPL26;RPL29;RPL28;UBA52;RPL12;RPL11;RPL36A;RPS15A;PSMB2;UBB;PSMB1;UBC;RPL14;RPS3;RPL15;RPS2;RPL18;RPS27A;RPL19;RPS25;RPS27;RPS29;RPL27A;PSMC2;RPS20;FAU;RPS21;RPS24;RPS23
Major Pathway Of rRNA Processing In Nucleolus And Cytosol R-HSA-6791226	62/179	3.0693E-46	1.4572E-44	16.91765341	1772.92743	RPL4;RPL5;RPL30;RPL3;RPL32;RPL31;RPL34;RPLP0;RPL10A;RPL8;RPL9;RPL6;RPL7;RPS4X;RPS14;RPL7A;RPS17;RPS16;RPL18A;RPS19;RPL36AL;RPL36;RPL35;RPL38;RPL37;RPS11;RPL39;RPS10;RPS13;RPL21;RPS7;RPL23;RPS5;RPL22;RPS6;RPL13A;RPSA;RPL26;RPL29;RPL28;UBA52;RPL12;RPL11;RPL36A;EXOSC4;RPS15A;RPL14;RPS3;RPL15;RPS2;RPL18;RPS27A;RPL19;RPS25;RPS27;RPS29;RPL27A;RPS20;FAU;RPS21;RPS24;RPS23
rRNA Processing In Nucleus And Cytosol R-HSA-8868773	62/189	1.3639E-44	6.1811E-43	15.57744526	1573.374471	RPL4;RPL5;RPL30;RPL3;RPL32;RPL31;RPL34;RPLP0;RPL10A;RPL8;RPL9;RPL6;RPL7;RPS4X;RPS14;RPL7A;RPS17;RPS16;RPL18A;RPS19;RPL36AL;RPL36;RPL35;RPL38;RPL37;RPS11;RPL39;RPS10;RPS13;RPL21;RPS7;RPL23;RPS5;RPL22;RPS6;RPL13A;RPSA;RPL26;RPL29;RPL28;UBA52;RPL12;RPL11;RPL36A;EXOSC4;RPS15A;RPL14;RPS3;RPL15;RPS2;RPL18;RPS27A;RPL19;RPS25;RPS27;RPS29;RPL27A;RPS20;FAU;RPS21;RPS24;RPS23
rRNA Processing R-HSA-72312	63/199	3.1885E-44	1.3822E-42	14.79886464	1482.168288	RPL4;RPL5;RPL30;RPL3;RPL32;RPL31;RPL34;RPLP0;RPL8;RPL10A;RPL9;RPL6;RPL7;RPS4X;RPS14;RPL7A;RPS17;RPS16;RPL18A;RPS19;RPL36AL;RPL36;RPL35;RPL38;RPL37;RPS11;RPL39;RPS10;RPS13;RPL21;RPS7;RPL23;RPS5;RPL22;RPS6;RPL13A;RPSA;RPL26;RPL29;RPL28;UBA52;RPL12;RPL11;RPL36A;HSD17B10;EXOSC4;RPS15A;RPL14;RPS3;RPL15;RPS2;RPL18;RPS27A;RPL19;RPS25;RPS27;RPS29;RPL27A;RPS20;FAU;RPS21;RPS24;RPS23

Cellular Responses To Stress R-HSA-2262752	101/722	1.073E-35	4.4574E-34	5.406437792	435.3265397	RPL4;RPL5;RPL30;RPL3;RPL32;RPL31;RPL34;RPL8;RPL10A;RPL9;RPL6;RPL7;RPS14;RPS17;PSMD7;RPS16;CREB3L3;RPL18A;RPS19;RPL36AL;RPL36;RPL35;RPL38;RPL37;RPS11;RPL39;RPS10;RPS13;RPL21;RPS7;RPL23;RPS5;RPL22;RPS6;RPSA;EEF1A1;COX7A2L;RPL26;KDELR3;RPL29;RPL28;UBA52;SQSTM1;HIGD1A;PRDX2;PRDX5;UBB;BAG1;UBC;ATP6V1F;JUN;NR1D1;RPS25;RPS27;GDNF;RPS29;RPL27A;RPS20;RPS21;RPS24;RPS23;CDKN1A;FKBP14;RPLP0;RPS4X;RPL7A;MYDGF;MAP1LC3B;ELOB;COX8A;ATP6V0B;GPX8;RPL13A;GPX7;DNAJC3;AKT1S1;CRYAB;DCTN2;RPL12;RPL11;DCTN3;RPL36A;COX5B;RPS15A;EXOSC4;PSMB2;PSMB1;RPS3;RPL14;RPL15;RPS2;RPL18;RPS27A;RPL19;EGLN1;UBL4A;YIF1A;PSMC2;LAMTOR2;P4HB;FAU
Cellular Responses To Stimuli R-HSA-8953897	101/736	5.7379E-35	2.2883E-33	5.283285548	416.5521414	RPL4;RPL5;RPL30;RPL3;RPL32;RPL31;RPL34;RPL8;RPL10A;RPL9;RPL6;RPL7;RPS14;RPS17;PSMD7;RPS16;CREB3L3;RPL18A;RPS19;RPL36AL;RPL36;RPL35;RPL38;RPL37;RPS11;RPL39;RPS10;RPS13;RPL21;RPS7;RPL23;RPS5;RPL22;RPS6;RPSA;EEF1A1;COX7A2L;RPL26;KDELR3;RPL29;RPL28;UBA52;SQSTM1;HIGD1A;PRDX2;PRDX5;UBB;BAG1;UBC;ATP6V1F;JUN;NR1D1;RPS25;RPS27;GDNF;RPS29;RPL27A;RPS20;RPS21;RPS24;RPS23;CDKN1A;FKBP14;RPLP0;RPS4X;RPL7A;MYDGF;MAP1LC3B;ELOB;COX8A;ATP6V0B;GPX8;RPL13A;GPX7;DNAJC3;AKT1S1;CRYAB;DCTN2;RPL12;RPL11;DCTN3;RPL36A;COX5B;RPS15A;EXOSC4;PSMB2;PSMB1;RPS3;RPL14;RPL15;RPS2;RPL18;RPS27A;RPL19;EGLN1;UBL4A;YIF1A;PSMC2;LAMTOR2;P4HB;FAU

Supplementary Table 18: Significantly enriched Gene Ontology Biological Processes terms for genes differentially expressed in Mfap5+/+ mouse fibroblasts vs Mfap5-/- mouse fibroblasts in day 7 post-wounding

Term	Overlap	P.value	Adjusted.P.value	Odds.Ratio	Combined.Score	Genes
Apoptotic Process (GO:0006915)	9/228	1.03781E-05	0.005870571	7.213796477	82.78416458	PPP1R15A;CSRNP1;BCL2L11;APAF1;PIK3CA;CASP3;PLAGL1;DDIT4;RHOB
Extracellular Matrix Organization (GO:0030198)	8/176	1.18478E-05	0.005870571	8.306363253	94.22215718	COL18A1;POSTN;ADAMTS14;COL27A1;LUM;HMCN1;ADAMTS9;MATN2
Positive Regulation Of Apoptotic Process (GO:0043065)	9/270	3.94422E-05	0.012726256	6.040024631	61.24992479	BCL2L11;BTG1;APAF1;CASP3;KCNMA1;HTRA1;SIK1;SOX4;RHOB
Negative Regulation Of Mitotic Cell Cycle (GO:0045930)	4/34	5.13673E-05	0.012726256	22.61994302	223.4060518	CDKN1C;BTG1;CDKN1B;ZFP36L2
Regulation Of Mitotic Cell Cycle (GO:0007346)	6/125	0.000112965	0.022389757	8.663500183	78.73760051	CDKN1C;BTG1;CDKN1B;DUSP3;SIK1;SOX9
Negative Regulation Of Protein Kinase Activity (GO:0006469)	5/88	0.00019574	0.032329768	10.28043207	87.78175082	CDKN1C;CDKN1B;DUSP3;HSPB1;ADARB1
Regulation Of Hepatocyte Proliferation (GO:2000345)	2/5	0.000358706	0.035547765	111.3501401	883.3414895	MDK;PTN
Notochord Development (GO:0030903)	2/5	0.000358706	0.035547765	111.3501401	883.3414895	COL18A1;COL27A1
Chondrocyte Development (GO:0002063)	2/5	0.000358706	0.035547765	111.3501401	883.3414895	SOX9;SULF2
Leukocyte Chemotaxis Involved In Inflammatory Response (GO:0002232)	2/5	0.000358706	0.035547765	111.3501401	883.3414895	MDK;PTN
Response To Glucocorticoid (GO:0051384)	3/26	0.000507307	0.04022117	21.94841562	166.5093277	BCL2L11;GOT1;ZFP36L2
Neurotrophin TRK Receptor Signaling Pathway (GO:0048011)	2/6	0.000535929	0.04022117	83.50840336	628.9442822	CASP3;DDIT4
Definitive Hemopoiesis (GO:0060216)	2/6	0.000535929	0.04022117	83.50840336	628.9442822	MFAP5;ZFP36L2
Cellular Response To Epidermal Growth Factor Stimulus (GO:0071364)	3/27	0.00056821	0.04022117	21.03283898	157.1788056	DUSP3;SOX9;ZFP36L2
Positive Regulation Of Programmed Cell Death (GO:0043068)	7/245	0.000734084	0.042851904	5.067337461	36.57040358	BCL2L11;APAF1;CASP3;KCNMA1;HTRA1;SOX4;RHOB
Response To Auditory Stimulus (GO:0010996)	2/7	0.000747331	0.042851904	66.80336134	480.917531	MDK;PTN
Positive Regulation Of Oligodendrocyte Differentiation (GO:0048714)	2/7	0.000747331	0.042851904	66.80336134	480.917531	MDK;PTN
Response To Epidermal Growth Factor (GO:0070849)	3/30	0.000778339	0.042851904	18.69303202	133.8112278	DUSP3;SOX9;ZFP36L2

Supplementary Table 19: Significantly enriched Reactome terms for genes differentially expressed in Mfap5+/+ mouse fibroblasts vs Mfap5-/- mouse fibroblasts in day 7 post-wounding

Term	Overlap	P.value	Adjusted.P.value	Odds.Ratio	Combined.Score	Genes
Extracellular Matrix Organization R-HSA-1474244	11/291	1.58496E-06	0.000664099	6.999642857	93.4798781	MFAP5;COL18A1;ADAMTS14;COL27A1;LUM;CASP3;COL6A2;COL6A1;HTRA1;ADAMTS9;ASPN
Collagen Biosynthesis And Modifying Enzymes R-HSA-1650814	5/67	5.3383E-05	0.011183742	13.77711346	135.5394894	COL18A1;ADAMTS14;COL27A1;COL6A2;COL6A1
Collagen Chain Trimerization R-HSA-8948216	4/44	0.000143508	0.018225762	16.95641026	150.0493314	COL18A1;COL27A1;COL6A2;COL6A1
RHO GTPases Activate CIT R-HSA-5625900	3/19	0.000195001	0.018225762	31.56197034	269.6183931	CDKN1B;MYL9;RHOB
Collagen Formation R-HSA-1474290	5/90	0.000217491	0.018225762	10.03752535	84.64998654	COL18A1;ADAMTS14;COL27A1;COL6A2;COL6A1
Signaling By PDGF R-HSA-186797	4/52	0.000275724	0.01925474	14.12464387	115.767127	PIK3CA;COL6A2;COL6A1;THBS2
Assembly Of Collagen Fibrils And Other Multimeric Structures R-HSA-2022090	4/57	0.000393051	0.023526924	12.78890502	100.2851011	COL18A1;COL27A1;COL6A2;COL6A1
Activation Of Caspases Thru Apoptosome-Mediated Cleavage R-HSA-111459	2/6	0.000535929	0.028069281	83.50840336	628.9442822	APAF1;CASP3
SMAC (DIABLO) Binds To IAPs R-HSA-111463	2/7	0.000747331	0.034792423	66.80336134	480.917531	APAF1;CASP3
SMAC, XIAP-regulated Apoptotic Response R-HSA-111469	2/8	0.0009925	0.041585738	55.66666667	384.9507996	APAF1;CASP3

Supplementary Table 20: Significantly enriched Gene Ontology Biological Processes terms for genes differentially

Term	Overlap	P.value	Adjusted.P.value	Odds.Ratio	Combined.Score	Genes
Positive Regulation Of Apoptotic Cell Clearance (GO:2000427)	2/7	3.39887E-05	0.003109966	332.8166667	3424.511256	C3;C4B
Regulation Of Apoptotic Cell Clearance (GO:2000425)	2/7	3.39887E-05	0.003109966	332.8166667	3424.511256	C3;C4B
Positive Regulation Of Phagocytosis (GO:0050766)	3/60	6.35342E-05	0.003875584	45.57665904	440.4497689	C3;C4B;CAMK1D
Skeletal System Development (GO:0001501)	3/149	0.000929087	0.04250571	17.71411554	123.6677082	SHOX2;PRELP;PCSK5

Supplementary Table 21: Significantly enriched reactome terms for genes differentially expressed in Mfap5-/- mouse fibroblasts vs Mfap5+/+ mouse fibroblasts in day 7 post-wounding

Term	Overlap	P.value	Adjusted.P.value	Odds.Ratio	Combined.Score	Genes
Activation Of C3 And C5 R-HSA-174577	3/6	3.88996E-08	4.66795E-06	868.3043478	14815.25373	C3;C4B;CFB
Regulation Of Complement Cascade R-HSA-977606	4/45	3.22335E-07	1.93401E-05	88.39467849	1321.29499	C3;C4B;SERPING1;CFB
Complement Cascade R-HSA-166658	4/55	7.31374E-07	2.9255E-05	71.02673797	1003.489973	C3;C4B;SERPING1;CFB
Initial Triggering Of Complement R-HSA-166663	3/21	2.55371E-06	7.66113E-05	144.6086957	1862.265502	C3;C4B;CFB
Post-translational Protein Phosphorylation R-HSA-8957275	3/106	0.000344267	0.008262403	25.16378219	200.658357	C3;IGFBP7;PRSS23
Regulation Of IGF Transport And Uptake By IGFBPs R-HSA-381426	3/123	0.000532182	0.010643633	21.58043478	162.6846608	C3;IGFBP7;PRSS23
Innate Immune System R-HSA-168249	6/1035	0.001784252	0.030587173	5.523323615	34.95576826	C3;C4B;ITPR2;SERPING1;CFB;DERA

Supplementary Table 25: Top 25 significant biological processes gene ontology terms for genes differentially upregulated in *Mfap5*$^{+/+}$ mouse fibroblasts versus *Mfap5*$^{-/-}$ mouse fibroblasts *in vitro*

Term	Overlap	P.value	Adjusted.P.value	Odds.Ratio	Combined.Score	Genes
Extracellular Matrix Organization (GO:0030198)	23/176	9.44E-15	1.96E-11	9.88	319.18	COL18A1;POSTN;COL15A1;ECM2;MMP2;COL11A1;TGFBR1;COL1A1;ADAMTS4;ADAMTS2;COL3A1;COL1A2;CREB3L1;COL5A3;SH3PXD2B;MMP17;SERPINH1;CYP1B1;COL8A1;COL10A1;TGFBI;ADAMTS9;GPM6B
Extracellular Structure Organization (GO:0043062)	17/109	1.67E-12	1.73E-09	11.95	323.95	POSTN;COL15A1;ECM2;MMP2;COL11A1;TGFBR1;COL1A1;ADAMTS4;ADAMTS2;COL3A1;COL1A2;COL5A3;MMP17;COL8A1;COL10A1;TGFBI;ADAMTS9
External Encapsulating Structure Organization (GO:0045229)	16/110	2.28E-11	1.58E-08	10.97	268.72	POSTN;COL15A1;ECM2;MMP2;COL11A1;COL1A1;ADAMTS4;ADAMTS2;COL3A1;COL1A2;COL5A3;MMP17;COL8A1;COL10A1;TGFBI;ADAMTS9
Collagen Fibril Organization (GO:0030199)	10/42	8.93E-10	4.64E-07	19.81	412.69	COL1A1;COL18A1;ADAMTS2;COL3A1;COL1A2;COL11A1;COL5A3;SERPINH1;CYP1B1;TGFBR1
Positive Regulation Of Epithelial To Mesenchymal Transition (GO:0010718)	10/47	2.93E-09	1.22E-06	17.13	336.49	COL1A1;TIAM1;IL6;NOTCH1;JAG1;RGCC;TGFB3;GCNT2;AXIN2;TGFBR1
Regulation Of Cell Population Proliferation (GO:0042127)	36/766	7.84E-09	2.72E-06	3.29	61.42	HDAC4;COL18A1;BTG2;NOTCH1;CXCL1;ADARB1;CX3CL1;CXCL5;SLC9A3R1;GCNT2;NCK2;CYP1B1;JAK2;ARID2;PDGFRB;EPHA4;JAG1;KSR1;TGFB3;NTRK3;CLEC11A;STAT3;ITGA1;LIF;LIFR;AXIN2;OSMR;TGFBR1;TIAM1;KAT2B;IL6;RGCC;IL6ST;PLPP1;HSPA1B;HSPA1A
Regulation Of Cell Migration (GO:0030334)	25/434	3.80E-08	1.13E-05	3.99	68.23	RET;NOTCH1;SERPINE2;ADARB1;CX3CL1;MYLK;TNN;GCNT2;MGAT3;CYP1B1;CTNNA2;SLIT2;ARID2;PDGFRB;EPHA4;NGFR;JAG1;LIMCH1;FZD4;NTRK3;STAT3;SULF1;TGFBR1;COL1A1;TIAM1
Negative Regulation Of Cell Population Proliferation (GO:0008285)	23/379	5.28E-08	1.37E-05	4.20	70.44	COL18A1;BTG2;NOTCH1;TGFB3;ITGA1;CXCL1;ADARB1;AXIN2;PER2;TGFBR3;KAT2B;SLC9A3R1;IL6;RGCC;TNN;NCK2;CYP1B1;JAK2;ARID2;SLC16A2;PLPP1;HSPA1B;HSPA1A
Positive Regulation Of Cell Differentiation (GO:0045597)	19/283	1.67E-07	3.85E-05	4.64	72.46	FBN2;TGFB3;TMEM64;AXIN2;TENT5A;TGFBR1;ACVR2A;COL1A1;TIAM1;HEYL;IL6;RGCC;GPC1;GCNT2;KCTD11;TCF4;ARID2;IL6ST;WNT4
Regulation Of Epithelial To Mesenchymal Transition (GO:0010717)	10/83	8.27E-07	1.72E-04	8.66	121.34	COL1A1;EPHA4;TIAM1;IL6;RGCC;TGFB3;GCNT2;AXIN2;TGFBR1;CLASP2
Regulation Of Osteoblast Differentiation (GO:0045667)	10/85	1.03E-06	1.96E-04	8.43	116.21	FBN2;IL6;NOTCH1;TNFAIP6;TNN;TENT5A;TMEM64;IL6ST;ACVR2A;WNT4
Transforming Growth Factor Beta Receptor Signaling Pathway (GO:0007179)	9/72	2.16E-06	3.44E-04	9.01	117.56	TGFBR3;COL3A1;COL1A2;TGFB3;STAT3;GCNT2;LRRC32;FMOD;TGFBR1
Negative Regulation Of Cell Adhesion (GO:0007162)	9/72	2.16E-06	3.44E-04	9.01	117.56	EPHA4;NOTCH1;JAG1;FZD4;GCNT2;CYP1B1;TGFBI;CORO2B;CX3CL1
Negative Regulation Of Cell Migration (GO:0030336)	13/163	2.32E-06	3.44E-04	5.51	71.53	EPHA4;NGFR;JAG1;LIMCH1;ADARB1;SULF1;CX3CL1;COL3A1;TNN;CYP1B1;SLIT2;ARID2;CLASP2
Cellular Response To Transforming Growth Factor Beta Stimulus (GO:0071560)	10/96	3.19E-06	4.43E-04	7.35	93.00	TGFBR3;COL3A1;COL1A2;TGFB3;STAT3;GCNT2;PDE3A;LRRC32;TGFBR1;WNT4
Enzyme-Linked Receptor Protein Signaling Pathway (GO:0007167)	11/124	5.03E-06	6.29E-04	6.16	75.21	PDGFRB;RET;NTRK3;LIF;LIFR;ROR1;IL6ST;JAK2;ANGPTL1;ACVR2A;FRK
Skeletal System Development (GO:0001501)	12/149	5.14E-06	6.29E-04	5.56	67.69	COL1A1;ADAMTS4;COL18A1;LAMA5;COL1A2;SH3PXD2B;COL10A1;PTH1R;SULF1;TGFBR1;FBN1;GLI2
Regulation Of Cell-Substrate Adhesion (GO:0010810)	7/44	5.80E-06	6.69E-04	11.87	143.17	ECM2;NOTCH1;FZD4;GCNT2;JAK2;CORO2B;CX3CL1
Regulation Of Collagen Biosynthetic Process (GO:0032965)	5/18	7.34E-06	7.63E-04	24.01	283.90	IL6;RGCC;TGFB3;CREB3L1;WNT4
Pulmonary Valve Morphogenesis (GO:0003184)	5/18	7.34E-06	7.63E-04	24.01	283.90	NOTCH2;HEYL;JAG1;NOTCH1;SLIT2
Eye Morphogenesis (GO:0048592)	5/19	9.83E-06	9.73E-04	22.30	257.09	FBN2;MFAP5;ALDH1A3;STAT3;FBN1
Notch Signaling Involved In Heart Development (GO:0061314)	4/10	1.25E-05	1.17E-03	41.51	468.54	NOTCH2;HEYL;JAG1;NOTCH1
Pulmonary Valve Development (GO:0003177)	5/20	1.29E-05	1.17E-03	20.81	234.23	NOTCH2;HEYL;JAG1;NOTCH1;SLIT2
Transmembrane Receptor Protein Tyrosine Kinase Signaling Pathway (GO:0007169)	16/284	1.49E-05	1.29E-03	3.81	42.37	RET;PDGFRB;EPHA4;MMP2;NTRK3;STAT3;PLAT;IDE;SULF1;KALRN;TIAM1;EFNB1;ROR1;JAK2;ANGPTL1;FRK
Positive Regulation Of Actin Filament Bundle Assembly (GO:0032233)	7/51	1.59E-05	1.32E-03	9.98	110.27	RGCC;LIMCH1;TGFB3;ARHGEF10L;SYNPO;CX3CL1;TGFBR1

Supplementary Table 26: Top 25 significant reactome pathway terms for genes differentially upregulated in *Mfap5*$^{+/+}$ mouse fibroblasts versus *Mfap5*$^{-/-}$ mouse fibroblasts *in vitro*

Term	Overlap	P.value	Adjusted. P.value	Odds.Ratio	Combined. Score	Genes
Extracellular Matrix Organization R-HSA-1474244	30/291	5.41E-16	3.72E-13	7.70	270.57	FBN2;COL18A1;LAMA5;COL15A1;COL11A1;ITGB3;LAMA4;ADAMTS4;ADAMTS2;TNN;SERPINH1;COL10A1;ADAMTS9;TGFB3;MMP2;ITGA1;COL1A1;MFAP5;COL3A1;VCAN;COL1A2;COL5A3;COL6A2;MMP17;ITGA11;COL6A1;COL8A1;SDC1;FMOD;FBN1
Collagen Biosynthesis And Modifying Enzymes R-HSA-1650814	13/67	4.13E-11	1.42E-08	15.39	367.99	COL18A1;COL15A1;COL11A1;COL1A1;ADAMTS2;COL3A1;COL1A2;COL6A2;COL5A3;COL6A1;SERPINH1;COL10A1;COL8A1
Collagen Chain Trimerization R-HSA-8948216	11/44	7.10E-11	1.46E-08	21.19	495.27	COL1A1;COL18A1;COL15A1;COL3A1;COL1A2;COL11A1;COL5A3;COL6A2;COL6A1;COL10A1;COL8A1
Assembly Of Collagen Fibrils And Other Multimeric Structures R-HSA-2022090	12/57	8.51E-11	1.46E-08	17.00	394.18	COL1A1;COL18A1;COL15A1;COL3A1;COL1A2;COL6A2;COL11A1;LAMA4;COL5A3;COL6A1;COL10A1;COL8A1
Collagen Formation R-HSA-1474290	14/90	1.64E-10	2.26E-08	11.80	265.88	COL18A1;COL15A1;COL11A1;LAMA4;COL1A1;ADAMTS2;COL3A1;COL1A2;COL6A2;COL5A3;COL6A1;SERPINH1;COL10A1;COL8A1
Interleukin-6 Family Signaling R-HSA-6783589	8/24	2.32E-09	2.65E-07	31.51	626.58	SOCS3;IL6;STAT3;LIF;LIFR;OSMR;IL6ST;JAK2
Signal Transduction R-HSA-162582	72/2465	2.29E-07	2.25E-05	2.10	32.06	RET;ITGB3;PLAT;ARHGEF10L;CXCL1;PTH1R;RND3;CX3CL1;CXCL5;MYLK;GLI2;CYP26B1;C1QTNF1;DBT;NCK2;TMED2;JAK2;CDON;DACT1;PDGFRB;KSR1;MMP2;NPY1R;AXIN2;TGFBR1;TIAM1;KAT2B;ALDH1A3;PLCB4;VGF;RASA3;COL6A2;CDC42EP3;COL6A1;TRIB3;PDE5A;IL6ST;NOTCH2;HDAC4;LAMA5;NOTCH1;LAMA4;ITPR1;SLC1A5;THBS2;HIF1A;KALRN;SOCS3;PAG1;WNT4;CLASP2;FZD1;NGFR;IL33;JAG1;PHC1;FZD4;TGFB3;NTRK3;STAT3;ARHGAP29;INHBB;PTPN13;ACVR2A;HEYL;IL6;COL1A2;GPAM;ARTN;CILP;PDE3A;FRK
Interleukin-6 Signaling R-HSA-1059683	5/11	4.34E-07	3.73E-05	52.05	762.52	SOCS3;IL6;STAT3;IL6ST;JAK2
Diseases Of Glycosylation R-HSA-3781865	13/143	5.24E-07	4.00E-05	6.37	92.09	NOTCH2;SPON2;NOTCH1;THBS2;ADAMTS4;ADAMTSL1;ADAMTS2;VCAN;ADAMTSL3;GPC1;SDC1;FMOD;ADAMTS9
Diseases Associated With O-glycosylation Of Proteins R-HSA-3906995	9/69	1.50E-06	1.03E-04	9.46	126.91	ADAMTS4;ADAMTSL1;NOTCH2;SPON2;ADAMTS2;NOTCH1;ADAMTSL3;THBS2;ADAMTS9
Defective B3GALTL Causes PpS R-HSA-5083635	7/37	1.71E-06	1.07E-04	14.65	194.49	ADAMTS4;ADAMTSL1;SPON2;ADAMTS2;ADAMTSL3;THBS2;ADAMTS9
O-glycosylation Of TSR Domain-Containing Proteins R-HSA-5173214	7/38	2.07E-06	1.19E-04	14.18	185.51	ADAMTS4;ADAMTSL1;SPON2;ADAMTS2;ADAMTSL3;THBS2;ADAMTS9
IL-6-type Cytokine Receptor Ligand Interactions R-HSA-6788467	5/17	5.37E-06	2.84E-04	26.02	315.70	LIF;LIFR;OSMR;IL6ST;JAK2
Interleukin-4 And Interleukin-13 Signaling R-HSA-6785807	10/107	8.52E-06	4.18E-04	6.51	76.02	SOCS3;LAMA5;IL6;COL1A2;CEBPD;MMP2;STAT3;LIF;JAK2;HIF1A
Diseases Of Metabolism R-HSA-5668914	15/247	1.14E-05	5.21E-04	4.12	46.93	NOTCH2;SPON2;NOTCH1;THBS2;ADAMTSL1;ADAMTS4;ADAMTS2;CYP26B1;VCAN;ADAMTSL3;GPC1;CYP1B1;SDC1;FMOD;ADAMTS9
Signaling By Interleukins R-HSA-449147	21/453	1.44E-05	6.19E-04	3.13	34.88	IL33;LAMA5;CEBPD;MMP2;STAT3;LIF;LIFR;CXCL1;NOD1;OSMR;PTPN13;HIF1A;SOCS3;IL6;IL1RL2;COL1A2;SDC1;ALPK1;JAK2;IL6ST;BRWD1
Signaling By PDGF R-HSA-186797	7/52	1.81E-05	7.33E-04	9.76	106.54	PDGFRB;COL6A2;COL6A1;STAT3;NCK2;PLAT;THBS2
ECM Proteoglycans R-HSA-3000178	7/55	2.64E-05	1.01E-03	9.15	96.43	LAMA5;VCAN;TNN;TGFB3;ITGB3;LAMA4;FMOD
O-linked Glycosylation R-HSA-5173105	9/107	5.57E-05	2.02E-03	5.78	56.64	ADAMTS4;ADAMTSL1;SPON2;ADAMTS2;ADAMTSL3;GCNT4;THBS2;ADAMTS9;GALNT10
MAPK1 (ERK2) Activation R-HSA-112411	3/9	3.17E-04	1.09E-02	31.03	249.98	IL6;IL6ST;JAK2
Degradation Of Extracellular Matrix R-HSA-1474228	8/109	3.69E-04	1.12E-02	4.97	39.29	ADAMTS4;COL18A1;LAMA5;COL15A1;MMP2;LAMA4;MMP17;ADAMTS9
Elastic Fibre Formation R-HSA-1566948	5/39	3.74E-04	1.12E-02	9.17	72.37	FBN2;MFAP5;TGFB3;ITGB3;FBN1
Laminin Interactions R-HSA-3000157	4/22	3.75E-04	1.12E-02	13.83	109.09	COL18A1;LAMA5;LAMA4;ITGA1
Cytokine Signaling In Immune System R-HSA-1280215	24/702	4.21E-04	1.18E-02	2.27	17.66	IFITM3;IL33;LAMA5;CEBPD;MMP2;STAT3;LIF;LIFR;CXCL1;NOD1;OSMR;PTPN13;HIF1A;ISG20;SOCS3;IL6;IL1RL2;COL1A2;SDC1;ALPK1;JAK2;IL6ST;BRWD1;GBP2
Signaling By Receptor Tyrosine Kinases R-HSA-9006934	19/496	4.33E-04	1.18E-02	2.54	19.68	PDGFRB;LAMA5;ITGB3;LAMA4;NTRK3;STAT3;ITPR1;PLAT;THBS2;HIF1A;TIAM1;VGF;CILP;COL6A2;COL6A1;NCK2;TRIB3;JAK2;PAG1

Table 26: Top 25 significant biological processes gene ontology terms for genes differentially upregulated in *Mfap5*$^{-/-}$ mouse fibroblasts versus *Mfap5*$^{+/+}$ mouse fibroblasts *in vitro*

Term	Overlap	P.value	Adjusted. P.value	Odds. Ratio	Combined. Score	Genes
Phagocytosis (GO:0006909)	17/69	3.10E-14	7.23E-11	16.30	506.85	MSR1;ITGAM;ANXA1;CD93;SLC11A1;ITGB2;PLD4;TREM2;AIF1;CORO1A;ARHGAP25;TYROBP;BIN2;ELMO1;CD14;FCGR2B;TGM2
Regulation Of Interleukin-6 Production (GO:0032675)	19/114	1.81E-12	2.10E-09	10.00	270.35	CD84;SYK;F2R;CARD9;CYBA;ARRB1;TREM2;POU2F2;AIF1;MAPK13;CD200R1;TYROBP;PLCG2;TLR8;F2RL1;NCKAP1L;TLR7;PTPN6;LCP1
Inflammatory Response (GO:0006954)	26/236	3.15E-12	2.45E-09	6.27	165.92	CSF1R;NRROS;C5AR1;ITGB2;ADM;F11R;ITGAL;AIF1;PIK3CG;C3AR1;BLNK;NLRP3;CD14;CCR1;ANXA1;VCAM1;SLC11A1;NFAM1;F2R;CYBB;CYBA;ADAM8;SIGLEC1;FCGR2B;CD44;PF4
Positive Regulation Of Phagocytosis (GO:0050766)	13/60	1.96E-10	1.14E-07	13.65	305.16	CAMK1D;FCER1G;SLC11A1;TREM2;CYBA;CFP;PTPRC;PLCG2;F2RL1;NCKAP1L;DOCK2;CD300LF;FCGR2B
Receptor-Mediated Endocytosis (GO:0006898)	17/122	4.96E-10	2.19E-07	8.05	172.42	MSR1;ITGAM;FCER1G;RAMP3;SYK;ITGB2;ADM;ARHGAP27;ARRB2;CXCL16;DAB2;MRC1;STAB1;ACKR3;SNX9;APOE;FCGR2B
Positive Regulation Of Cytokine Production (GO:0001819)	27/320	5.64E-10	2.19E-07	4.66	99.14	CSF1R;FLT4;AIF1;PIK3CG;PLCG2;NLRP3;CD14;HAVCR2;ANXA1;FCER1G;CADM1;SYK;SLC11A1;NFAM1;CARD9;F2R;LAPTM5;CYBA;POU2F2;MAPK13;CCDC88B;TYROBP;PTPRC;TLR8;F2RL1;TLR7;LCP1
Macrophage Activation (GO:0042116)	10/35	1.33E-09	4.44E-07	19.62	400.84	C1QA;ITGAM;CD93;SYK;SLC11A1;C5AR1;ITGB2;PLCG2;TREM2;AIF1
Endocytosis (GO:0006897)	20/189	2.14E-09	6.23E-07	5.91	117.96	DENND1C;MSR1;ITGAM;ANXA1;CD93;SLC11A1;IL10RA;ITGB2;PLD4;ARHGAP27;CORO1A;CXCL16;DAB2;DNER;MRC1;STAB1;SNX9;APOE;CD14;FCGR2B
Microglial Cell Activation (GO:0001774)	8/20	2.90E-09	7.52E-07	32.55	639.86	CX3CR1;C1QA;ITGAM;TYROBP;C5AR1;ITGB2;TREM2;AIF1
Positive Regulation Of Interleukin-6 Production (GO:0032755)	13/76	4.30E-09	9.94E-07	10.18	196.03	SYK;F2R;CARD9;CYBA;POU2F2;AIF1;MAPK13;TYROBP;PLCG2;TLR8;F2RL1;TLR7;LCP1
Negative Regulation Of Cytokine Production (GO:0001818)	19/178	4.69E-09	9.94E-07	5.95	114.19	CD84;ANXA1;PPP1R11;SLC11A1;LAPTM5;ARRB1;TREM2;VSIR;CD200R1;TYROBP;PTPRC;TLR8;F2RL1;NCKAP1L;PTPN6;FCGR2B;TRIB2;CD33;HAVCR2

Leukocyte Cell-Cell Adhesion (GO:0007159)	9/31	7.76E-09	1.51E-06	20.01	373.76	VCAM1;ITGA4;SEMA4D;SYK;ITGB2;F11R;ITGAL;JAM2;FERMT3
Positive Regulation Of Cell Migration (GO:0030335)	23/272	1.05E-08	1.88E-06	4.63	85.04	CCR1;CSF1R;SEMA4D;SEMA6D;FLT4;SEMA3A;F2R;TWIST2;AIF1;VSIR;CXCL16;SHTN1;DAB2;PTPRC;PLAU;PLCG2;F2RL1;SPRY2;NCKAP1L;ITGA6;LGR6;EPHA2;FERMT3
Negative Regulation Of Leukocyte Activation (GO:0002695)	7/17	2.32E-08	3.77E-06	34.10	599.44	MILR1;CD84;CD300A;CD300LF;FCGR2B;CD33;HAVCR2
B Cell Receptor Signaling Pathway (GO:0050853)	10/46	2.42E-08	3.77E-06	13.61	238.73	VAV3;LAT2;PTPRC;SYK;PRKCB;NFAM1;BLNK;PLCG2;BTK;NCKAP1L
Plasma Membrane Invagination (GO:0099024)	8/26	3.23E-08	4.66E-06	21.69	374.16	MSR1;ITGAM;BIN2;ITGB2;ELMO1;SNX9;FCGR2B;ARHGAP25
Regulation Of ERK1 And ERK2 Cascade (GO:0070372)	21/244	3.40E-08	4.66E-06	4.70	80.84	CCR1;CSF1R;RAMP3;SYK;FLT4;CARD9;F2R;C5AR1;ARRB1;TREM2;ARRB2;HTR2A;DUSP6;PTPRC;F2RL1;ACKR3;SPRY2;PTPN6;APOE;CD44;EPHA2
Actin Filament Organization (GO:0007015)	16/144	4.42E-08	5.72E-06	6.19	104.84	WAS;COBL;CAPG;CORO1A;ARHGAP25;AIF1;EPS8;ACTA1;DPYSL3;ELMO1;RAC2;HCLS1;LCP1;CGNL1;MYO1F;MYO1G
Regulation Of Tumor Necrosis Factor Production (GO:0032680)	15/127	5.14E-08	6.31E-06	6.62	111.13	CX3CR1;CD84;SYK;HSPB1;CYBA;VSIR;TYROBP;PTPRC;PLCG2;PTPN6;CD14;LCP1;CD33;HAVCR2;PF4
Phagocytosis, Engulfment (GO:0006911)	8/30	1.13E-07	1.31E-05	17.75	283.90	MSR1;ITGAM;BIN2;ITGB2;ELMO1;FCGR2B;AIF1;ARHGAP25
Positive Regulation Of MAPK Cascade (GO:0043410)	23/310	1.18E-07	1.31E-05	4.01	63.95	CCR1;CSF1R;RAMP3;FLT4;CARD9;F2R;C5AR1;LAPTM5;ARRB1;TREM2;ARRB2;HTR2A;ADRA1B;PIK3CG;GHR;PTPRC;PLCG2;F2RL1;ACKR3;SPRY2;ADAM8;APOE;CD44
Regulation Of Small GTPase Mediated Signal Transduction (GO:0051056)	14/118	1.36E-07	1.44E-05	6.64	105.02	ARHGAP9;VAV3;DOCK8;ARHGAP28;ARHGAP27;ARHGAP25;VAV1;ARHGAP45;ARHGAP4;PREX1;ARHGAP30;DOCK2;CGNL1;TGM2
Positive Regulation Of ERK1 And ERK2 Cascade (GO:0070374)	17/179	1.70E-07	1.67E-05	5.20	81.06	CCR1;CSF1R;RAMP3;FLT4;F2R;C5AR1;CARD9;ARRB1;TREM2;ARRB2;HTR2A;PTPRC;ACKR3;F2RL1;SPRY2;APOE;CD44
Synapse Pruning (GO:0098883)	5/8	1.86E-07	1.67E-05	80.81	1252.42	C1QB;CX3CR1;ITGAM;TREM2;C1QC
Negative Regulation Of Leukocyte Degranulation (GO:0043301)	5/8	1.86E-07	1.67E-05	80.81	1252.42	CD84;SPI1;CD300A;NCKAP1L;FCGR2B

Table 27: Top 25 significant reactome pathway terms for genes differentially upregulated in *Mfap5*$^{-/-}$ mouse fibroblasts versus *Mfap5*$^{+/+}$ mouse fibroblasts *in vitro*

Term	Overlap	P.value	Adjusted. P.value	Odds. Ratio	Combined. Score	Genes
Innate Immune System R-HSA-168249	76/1035	9.75633E-23	7.80507E-20	4.434148434	224.72947	ARHGAP9;TINAGL1;ITGAM;NCF1;NCF2;NCF4;ITGB2;TREM2;ITGAL;CTSS;LGALS3;GLIPR1;PLAU;RPS6KA2;CLEC5A;RPS6KA1;C3AR1;RAC2;COTL1;B2M;CTSD;CD33;CTSB;MAP2K3;VAV3;FCER1G;SYK;CD93;S100A1;CD300A;NFAM1;SLC11A1;CD180;CYBB;IRAG2;CYBA;MMP9;DUSP6;VAV1;ARHGAP45;MMP12;CLEC4D;CDC34;TYROBP;BIN2;SLC7A8;BTK;ELMO1;TLR8;ADAM8;LCP2;TLR7;DOCK2;CD44;C1QB;C1QA;C5AR1;NLRC5;WAS;PLD4;CFP;PDZD2;PLCG2;CD300LB;NLRP3;NCKAP1L;CD55;LAIR1;CARD9;LIMK1;MAPK13;PTPRC;PTPN6;CD68;BCL2L1;C1QC
Immune System R-HSA-168256	104/1943	1.45323E-20	5.8129E-18	3.291539415	150.35066	NCF1;NCF2;NCF4;IFIT2;LGALS3;PLAU;RPS6KA2;CLEC5A;RPS6KA1;C3AR1;AP1S2;COTL1;B2M;MAP2K3;CD93;PRKCB;S100A1;CD300A;SLC11A1;CD180;TALDO1;CYBB;IRAG2;CYBA;ARHGAP45;CLEC4D;CDC34;TYROBP;BIN2;BTK;ELMO1;ADAM8;CSF1R;C5AR1;PLD4;CSF2RB;CFP;PDZD2;INPP5D;PLCG2;CD300LB;NDN;NLRP3;NCKAP1L;CD300LD;CD300LF;CCR1;VCAM1;SMAD7;PTPRC;BCL2L1;ARHGAP9;TINAGL1;ITGAM;ITGB2;TREM2;ITGAL;CTSS;GHR;GLIPR1;MRC1;RAC2;BLNK;CTSD;CD33;HAVCR2;CTSB;VAV3;ANXA1;FCER1G;ITGA4;SYK;GSTO1;NFAM1;MMP9;DUSP6;VAV1;MMP12;CD200R1;SLC7A8;TLR8;IRF8;LCP2;TLR7;DOCK2;CD44;C1QB;C1QA;NLRC5;WAS;STX3;CD55;LAIR1;IL10RA;CARD9;LIMK1;MAPK13;PTPN6;SIGLEC1;PTPN7;FCGR2B;PIK3AP1;CD68;C1QC
Hemostasis R-HSA-109582	47/576	5.73754E-16	1.53001E-13	4.67845095	164.18711	TOR4A;CD84;ITGAM;SELPLG;DOCK8;ITGB2;PLEK;ENDOD1;ATP2A3;ARRB1;ARRB2;F11R;ITGAL;PIK3CG;GNG2;GRB14;PLAU;INPP5D;PLCG2;RAC2;JAM2;S100A10;VAV3;FCER1G;ITGA4;SYK;PRKCB;ANXA5;F2R;L1CAM;GNG11;TUBA4A;VAV1;PROCR;PRKAR1B;SLC7A8;CD109;GNB4;LCP2;ITGA6;PTPN6;CD48;ZFPM2;DOCK2;CD44;FERMT3;PF4
Neutrophil Degranulation R-HSA-6798695	41/468	4.28165E-15	8.56331E-13	5.000280012	165.43145	ARHGAP9;TINAGL1;ITGAM;ITGB2;C5AR1;CFP;ITGAL;CTSS;LGALS3;GLIPR1;PLAU;CLEC5A;C3AR1;COTL1;NCKAP1L;B2M;CTSD;CD33;CD55;LAIR1;CTSB;FCER1G;CD93;CD300A;NFAM1;SLC11A1;CYBB;IRAG2;CYBA;MMP9;ARHGAP45;MMP12;CLEC4D;PTPRC;TYROBP;BIN2;ADAM8;PTPN6;CD68;DOCK2;CD44

Signal Transduction R-HSA-162582	100/2465	7.49012E-12	1.19842E-09	2.35719124	60.385196	NCF1;NCF2;NCF4;STMN2;IKZF1;F11R;CXCL16;AMOT;PREX1;RPS6KA2;RPS6KA1;ARHGDIB;C3AR1;TLE1;H2AC6;PRKCB;F2R;CYBB;CYBA;DKK2;ARHGAP45;PRKAR1B;BTK;ELMO1;ABCG1;EPHA2;ADCYAP1R1;CRABP1;C5AR1;CSF2RB;RASAL3;LTBP1;PDZD2;FLRT2;PLCG2;NLRP3;NCKAP1L;ST3GAL6;APOE;CCR1;RAB4A;ARHGAP28;ARHGAP27;GNG11;ARHGAP25;ZWINT;SMAD7;ARHGAP30;ID1;MDM2;GNB4;RGL2;BCL2L1;ARHGAP9;GABRB1;FLT4;DOCK8;ATP2A3;HSPB1;ADM;ARRB1;HTR2A;ARRB2;ADRA1B;PIK3CG;ARHGAP4;HEBP1;ADGRE1;DNER;RAC2;CTSD;VAV3;ANXA1;SYK;ARAP3;MMP9;DUSP6;VAV1;CD200R1;RASA4;DOCK2;CX3CR1;RGS17;RAMP3;WAS;GNG2;CD55;LIMK1;CFLAR;MAPK13;FMNL1;DHRS9;F2RL1;SPRY2;ACKR3;PTPN6;PTPN7;LGR6;PIK3AP1;PF4
RAC1 GTPase Cycle R-HSA-9013149	22/178	1.56E-11	2.08E-09	7.082256675	176.23311	ARHGAP9;VAV3;NCF1;NCF2;DOCK8;NCF4;WAS;CYBB;ARAP3;CYBA;ARHGAP27;ARHGAP25;VAV1;ARHGAP45;ARHGAP4;PREX1;FMNL1;ARHGAP30;ARHGDIB;NCKAP1L;DOCK2;EPHA2
Cell Surface Interactions At Vascular Wall R-HSA-202733	19/134	3.43585E-11	3.92669E-09	8.250702341	198.79383	CD84;ITGAM;FCER1G;SELPLG;ITGA4;ITGB2;F11R;L1CAM;ITGAL;PROCR;GRB14;SLC7A8;INPP5D;CD48;ITGA6;PTPN6;CD44;JAM2;PF4
Platelet Activation, Signaling And Aggregation R-HSA-76002	24/254	5.38678E-10	5.38678E-08	5.247476002	111.99112	VAV3;TOR4A;FCER1G;SYK;PRKCB;ANXA5;F2R;PLEK;ENDOD1;ARRB1;ARRB2;GNG11;VAV1;TUBA4A;PIK3CG;GNG2;CD109;PLCG2;RAC2;GNB4;LCP2;PTPN6;FERMT3;PF4
Immunoregulatory Interactions Between A Lymphoid And A non-Lymphoid Cell R-HSA-198933	16/123	4.49398E-09	3.99465E-07	7.413474115	142.49087	VCAM1;ITGA4;CD300A;ITGB2;TREM2;ITGAL;CD200R1;TYROBP;CD300LB;CD300LD;SIGLEC1;CD300LF;FCGR2B;B2M;CD33;LAIR1
GPVI-mediated Activation Cascade R-HSA-114604	9/32	1.0596E-08	8.4768E-07	19.1426087	351.51169	VAV3;FCER1G;SYK;PLCG2;RAC2;LCP2;PTPN6;PIK3CG;VAV1
DAP12 Interactions R-HSA-2172127	10/43	1.20341E-08	8.75209E-07	14.85380117	270.86678	VAV3;TYROBP;SYK;CLEC5A;CD300LB;PLCG2;BTK;TREM2;LCP2;B2M
RHO GTPases Activate NADPH Oxidases R-HSA-5668599	8/24	1.57726E-08	1.05151E-06	24.40773067	438.48467	PDZD2;NCF1;NCF2;PRKCB;NCF4;RAC2;CYBB;CYBA

Adaptive Immune System R-HSA-1280218	40/733	1.83952E-08	1.13201E-06	2.956080354	52.651274	NCF1;NCF2;NCF4;ITGB2;WAS;TREM2;ITGAL;CTSS;INPP5D;MRC1;AP1S2;PLCG2;CD300LB;BLNK;CD300LD;CD300LF;B2M;CTSD;CD33;LAIR1;CTSB;VCAM1;ITGA4;SYK;PRKCB;S100A1;CD300A;CYBB;CYBA;VAV1;CD200R1;PTPRC;CDC34;TYROBP;BTK;LCP2;PTPN6;SIGLEC1;FCGR2B;PIK3AP1
DAP12 Signaling R-HSA-2424491	8/28	6.20433E-08	3.54533E-06	19.52219451	323.97928	VAV3;TYROBP;SYK;PLCG2;BTK;TREM2;LCP2;B2M
Signaling By VEGF R-HSA-194138	13/102	1.62927E-07	8.68946E-06	7.193451368	112.43337	VAV3;NCF1;NCF2;PRKCB;FLT4;NCF4;CYBB;HSPB1;CYBA;VAV1;MAPK13;ELMO1;NCKAP1L
Cross-presentation Of Particulate Exogenous Antigens (Phagosomes) R-HSA-1236973	5/8	1.85802E-07	9.29009E-06	80.80858086	1252.4187	NCF1;NCF2;NCF4;CYBB;CYBA
RAC2 GTPase Cycle R-HSA-9013404	12/87	2.02639E-07	9.53596E-06	7.865390428	121.22013	VAV3;PREX1;NCF1;NCF2;NCF4;RAC2;CYBB;CYBA;NCKAP1L;DOCK2;VAV1;EPHA2
VEGFA-VEGFR2 Pathway R-HSA-4420097	12/93	4.26211E-07	1.89427E-05	7.2805299	106.79323	VAV3;NCF1;NCF2;PRKCB;NCF4;ELMO1;HSPB1;CYBB;CYBA;NCKAP1L;VAV1;MAPK13
GPCR Downstream Signaling R-HSA-388396	33/619	5.81558E-07	2.44867E-05	2.846402767	40.867383	CX3CR1;RGS17;ADCYAP1R1;RAMP3;C5AR1;ADM;ARRB1;HTR2A;ARRB2;ADRA1B;PIK3CG;CXCL16;HEBP1;PREX1;GNG2;RPS6KA2;RPS6KA1;C3AR1;NLRP3;CCR1;VAV3;ANXA1;PRKCB;F2R;GNG11;VAV1;CD200R1;PRKAR1B;BTK;GNB4;F2RL1;ACKR3;PF4
Semaphorin Interactions R-HSA-373755	10/64	6.4852E-07	2.59408E-05	9.067576348	129.20002	TYROBP;CD72;PTPRC;SEMA4D;SEMA6D;SEMA3A;DPYSL3;LIMK1;CRMP1;TREM2
Signaling By GPCR R-HSA-372790	35/689	8.05477E-07	3.06848E-05	2.709754043	38.022813	CX3CR1;RGS17;ADCYAP1R1;RAMP3;C5AR1;ADM;ARRB1;HTR2A;ARRB2;ADRA1B;PIK3CG;CXCL16;HEBP1;ADGRE1;PREX1;GNG2;RPS6KA2;RPS6KA1;C3AR1;NLRP3;CD55;CCR1;VAV3;ANXA1;PRKCB;F2R;GNG11;VAV1;CD200R1;PRKAR1B;BTK;GNB4;F2RL1;ACKR3;PF4
Leishmania Infection R-HSA-9658195	19/247	8.67691E-07	3.15524E-05	4.137393162	57.747375	VAV3;ENTPD1;ADCYAP1R1;RAMP3;SYK;WAS;ADM;CYBA;GNG11;VAV1;GNG2;PRKAR1B;C3AR1;BTK;PLCG2;ELMO1;GNB4;NLRP3;NCKAP1L
Toll-like Receptor Cascades R-HSA-168898	15/162	1.25273E-06	4.35731E-05	5.035740184	68.436662	MAP2K3;ITGAM;S100A1;CD180;ITGB2;NLRC5;CTSS;DUSP6;RPS6KA2;RPS6KA1;BTK;PLCG2;TLR8;TLR7;CTSB

Other Semaphorin Interactions R-HSA-416700	6/19	1.52662E-06	5.08874E-05	22.42183623	300.28339	CD72;PTPRC;TYROBP;SEMA4D;SEMA6D;TREM2
Antigen Activates B Cell Receptor Leading To Second Messenger Generation R-HSA-983695	7/31	2.44489E-06	7.66298E-05	14.19662106	183.44181	SYK;BLNK;PLCG2;BTK;PTPN6;PIK3AP1;VAV1
